# Rational design and biological validation of EZH2/PD-L1 bifunctional inhibitors for colorectal cancer immunotherapy

**DOI:** 10.3389/fimmu.2026.1886069

**Published:** 2026-07-30

**Authors:** Bingsheng Guan, Binbin Cheng, Hongqiao Li

**Affiliations:** 1 Department of Gastrointestinal Surgery, First Affiliated Hospital of Jinan University, Guangzhou, China; 2 Hubei Polytechnic University, Huangshi, Hubei, China; 3 Huangshi Central Hospital, Huangshi, China

**Keywords:** bifunctional inhibitors, cancer immunotherapy, colorectal cancer (CRC), EZH2, PD−1/PD−L1

## Abstract

**Objectives:**

This study aims to identify novel small-molecule inhibitors that target both PD-L1 and EZH2 to enhance colorectal cancer immunotherapy.

**Methods:**

A combination of computer-aided molecular simulation, homogeneous time-resolved fluorescence (HTRF) assays, biolayer interferometry (BLI) assays, microscale thermophoresis (MST), and MTase-Glo methyltransferase assays was used to identify compounds with dual-targeting potential.

**Results:**

Compound PE-1 exhibited potent inhibitory activity against the PD-1/PD-L1 interaction (IC_50_ = 48.1 nM) and showed strong EZH2 methyltransferase inhibitory effects, with an IC_50_ of 101.2 nM. BLI and MST further confirmed that PE-1 can effectively bind to both PD-L1 and EZH2 at the molecular level, supporting its bifunctional targeting capability. Importantly, compound PE-1 demonstrated significant activity in a PD-1/PD-L1 NFAT reporter bioassay, dose-dependently enhancing luciferase activity (EC_50_ = 0.61 μM), indicating effective blockade of the PD-1/PD-L1 pathway and reactivation of T-cell NFAT signaling. Notably, PE-1 demonstrated favorable *in vivo* pharmacokinetic properties, including a satisfactory oral bioavailability of 58.4%. In syngeneic tumor models, oral administration of PE-1 elicited substantial anti-tumor efficacy, with tumor growth inhibition (TGI) reaching 69.9% in CT26 tumors.

**Conclusions:**

PE-1 demonstrates dual-target inhibitory activity against the PD-1/PD-L1 immune checkpoint and EZH2, underscoring its potential as a lead compound for the development of next-generation bifunctional anticancer agents.

## 1 Introduction

PD-1/PD-L1-directed immunotherapy has revolutionized oncological care by reactivating endogenous T-cell-mediated immunity to eliminate malignant cells (1, 2). However, clinical experience has uncovered two major limitations: a significant proportion of patients manifest primary resistance, exhibiting no initial response, whereas others initially respond but subsequently develop acquired resistance, culminating in disease progression (3, 4). As a result, objective response rates (ORRs) for PD-1/PD-L1 monotherapies remain modest, typically 15%-25% across solid tumors, highlighting a persistent therapeutic gap. These clinical findings highlight a key biological insight: blockade of the PD-1/PD-L1 pathway alone is typically insufficient to overcome the multi-layered and overlapping immunosuppressive mechanisms within the tumor microenvironment (TME), thereby limiting the magnitude of anti-tumor immunity (5, 6). Therefore, identifying and validating novel immunomodulatory targets is critical to broaden and amplify the clinical benefits of immuno-oncology therapies. Such targets necessitate favorable safety profiles, potent single-agent or combinatorial activity, and mechanistic synergy with PD-1/PD-L1 inhibition (2, 7).

**Figure 1:**
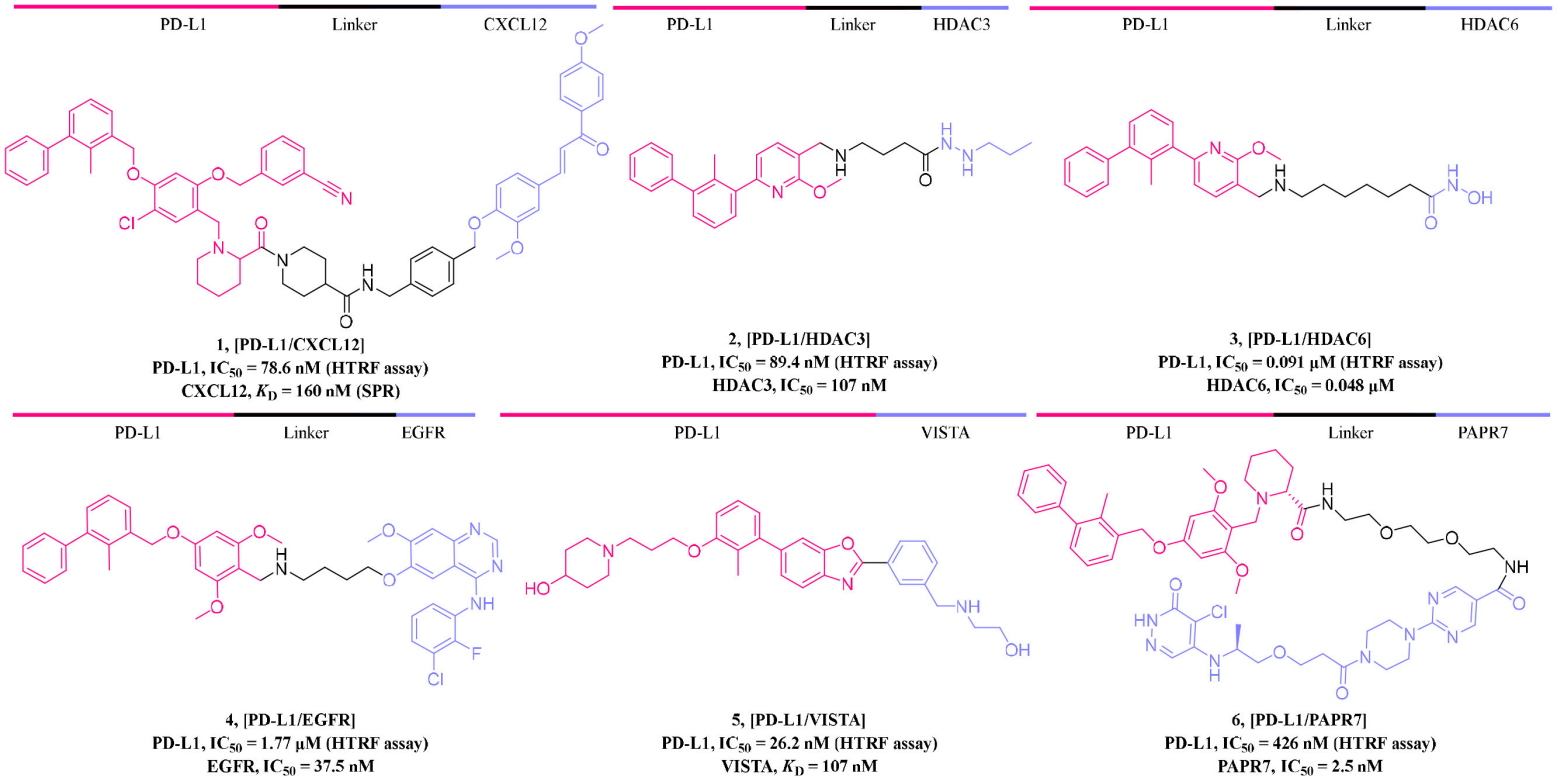
Representative chemical structures of bifunctional inhibitors focused on PD-L1.

Building on our prior work, we systematically interrogated a focused panel of PD-L1-centered dual-target inhibitors, including agents that co-target PD-L1 with NAMPT or VISTA (8, 9). These investigations conclusively demonstrated that dual-targeting strategies, rationally designed to exploit complementary signaling pathways governing tumorigenesis and immune suppression, substantially enhance anti-tumor efficacy, surpassing conventional single-target monotherapies in preclinical evaluations (10). Furthermore, corroborating evidence from previous research has reinforced this paradigm: multiple series of bispecific agents co-targeting PD-L1 with HDAC3, EGFR, VISTA, or PARP7 have been reported (3, 8, 9, 11–14). These collective findings underscore the feasibility of rationally engineering sophisticated PD-L1-focused bifunctional inhibitors, wherein structural integration of distinct pharmacophores enables concurrent modulation of key oncogenic and immune-regulatory pathways to overcome therapeutic resistance (**Figure 1**) (15–18).

EZH2 is a key catalytic component of the polycomb repressive complex 2 (PRC2) and functions as an essential histone methyltransferase (19–21). It exerts epigenetic regulatory functions by catalyzing the tri-methylation of histone H3 at lysine 27 (H3K27me3). This modification facilitates transcriptional silencing of target genes involved in cell cycle progression, differentiation, and immune-response regulation (22, 23). Notably, EZH2 has emerged as a critical regulator of anti-tumor immunity and TME. Its dysregulation, encompassing both overexpression and gain-of-function mutations, is intimately linked with the progression of diverse malignancies. Mechanistically, EZH2-mediated H3K27me3 modification directly inhibits the expression of immune-related genes, fostering an immunosuppressive environment within the TME.

**Figure 2:**
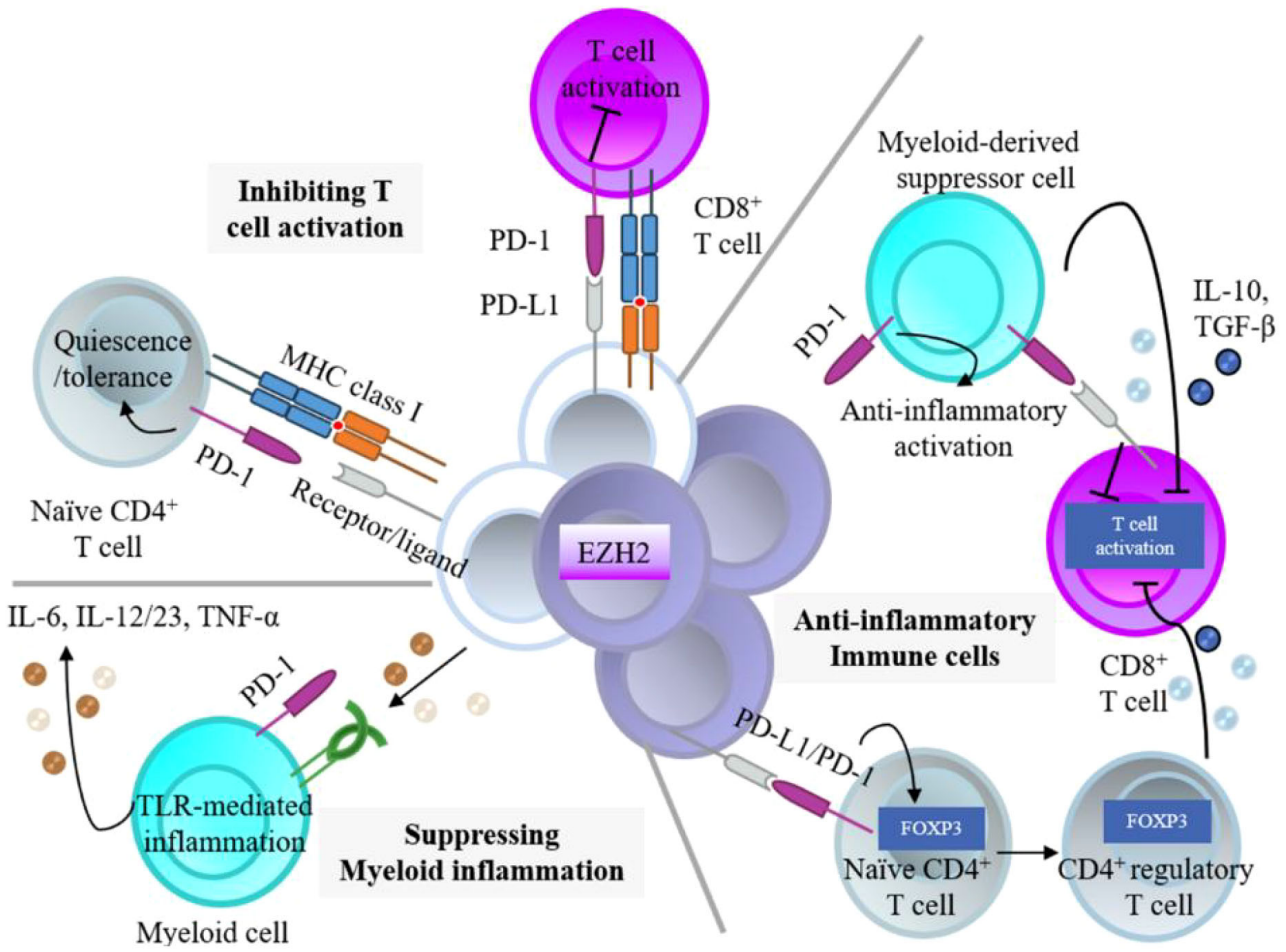
The multifaceted roles of EZH2 in regulating the TME are noteworthy. EZH2 exerts numerous immunosuppressive effects within the TME by directly impairing the anti-tumor effector functions of T cells, suppressing inflammatory activation in myeloid-derived immune cells, and expanding immunosuppressive cell populations. Collectively, these actions significantly undermine effective anti-tumor immune responses.

It compromises effector function and CD8^+^ cytotoxic T cell infiltration, promotes the polarization of pro-tumorigenic M2 macrophages, and expands immunosuppressive subsets, including Tregs and myeloid-derived suppressor cells (MDSCs). Conversely, pharmacological inhibition or genetic ablation of EZH2 has been shown to restore the infiltration and activation of CD8^+^ T cells in prostate and colorectal cancer models, thereby reversing immunosuppression. Moreover, tumors characterized by elevated EZH2 activity or stability frequently exhibit intrinsic resistance to PD-1/PD-L1 immunotherapy (24–26). The EZH2-driven immunosuppressive tumor microenvironment engenders a “cold tumor” phenotype that is refractory to immune-checkpoint blockade. Such interaction between EZH2-mediated epigenetic regulation and immune evasion underscores the rationale for co-targeting EZH2 and the PD-1/PD-L1 pathways. Such an approach seeks to surmount immunotherapy resistance and enhance anti-tumor immune responses. Collectively, these findings accentuate EZH2 as a multifunctional oncogenic driver and a critical immune modulator, underscoring its potential as a therapeutic target for combinatorial immunotherapeutic strategies in cancer management (**Figure 2**).

**Figure 3:**
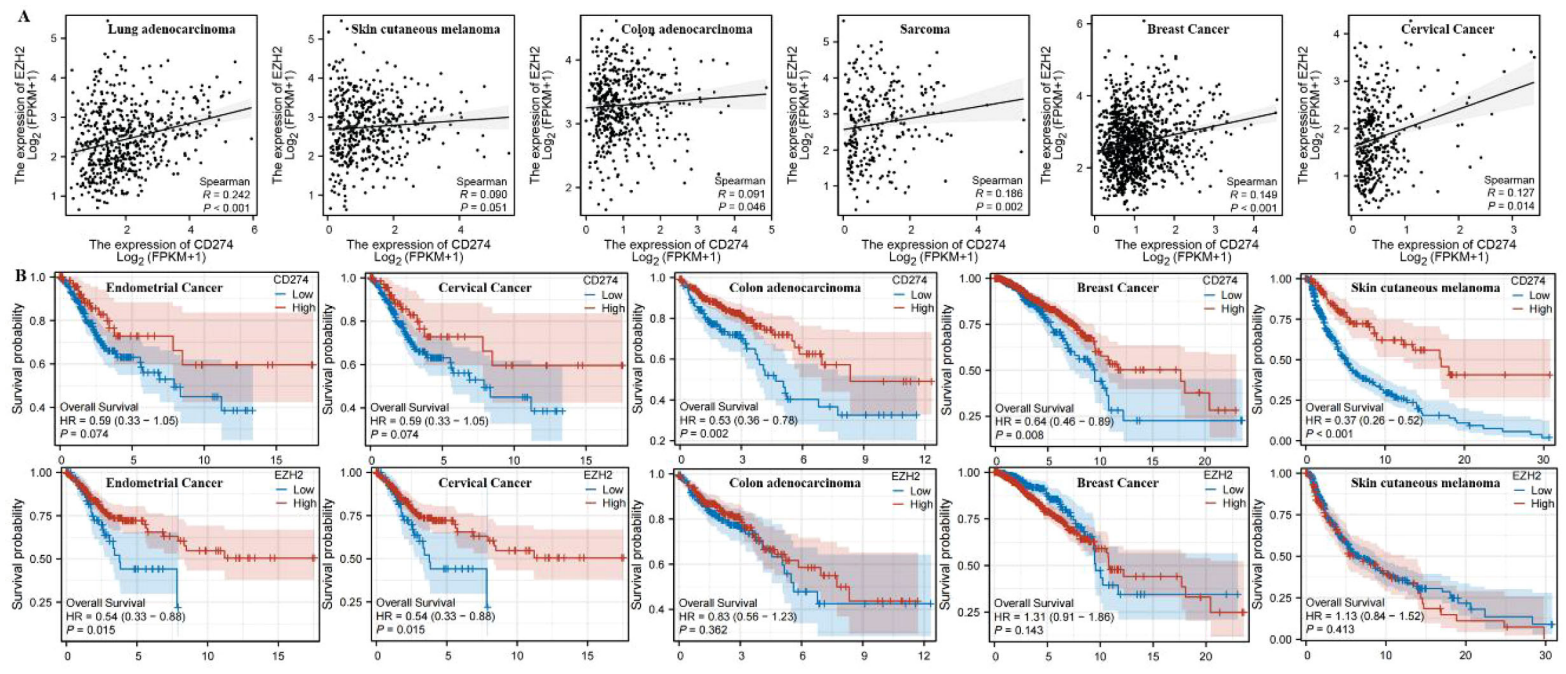
**(A)** Correlation analysis of PD-L1 and EZH2 expression across diverse tumor types. **(B)** Survival curves stratified by PD-L1 and EZH2 gene expression levels in multiple tumor cohorts.

To delineate the biological interplay between PD-L1 and EZH2, we leveraged The Cancer Genome Atlas (TCGA) datasets to perform targeted correlation analyses focusing on CD274 (gene encoding PD-L1) and EZH2. Our results revealed concurrent upregulation of EZH2 and CD274 expression across a spectrum of tumor types, with notable enrichment in colon adenocarcinoma. A positive correlation was identified between EZH2 and CD274 expression levels, indicating potential functional crosstalk between these two molecules in malignant contexts (**Figure 3A**). Furthermore, survival analyses across large patient cohorts within the TCGA database demonstrated a significant association between elevated co-expression of CD274 and EZH2 and improved overall survival. Kaplan-Meier (K-M) survival curves clearly stratified patient outcomes between high and low expression of CD274 or EZH2. These findings underscore the prognostic relevance of CD274 and EZH2, validating their critical roles in modulating disease progression and shaping clinical outcomes for cancer patients (**Figure 3B**).

**Figure 4:**
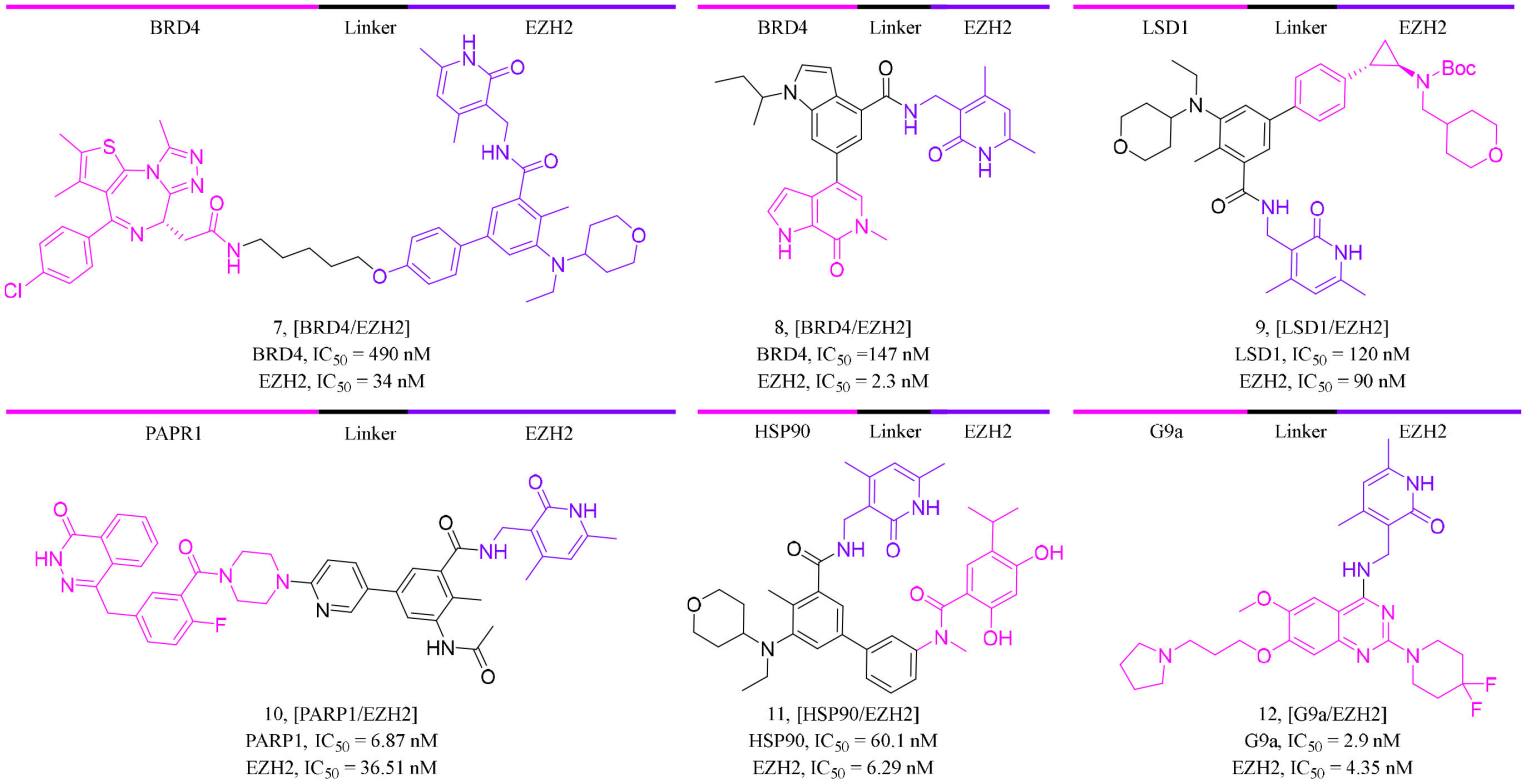
The representative rationally designed dual-target small-molecule inhibitors targeting EZH2.

To date, numerous EZH2-targeted bifunctional inhibitors have been reported, with most progressing through various phases of preclinical and clinical development (24, 25, 27, 28). This progress is rooted in EZH2’s well-documented functions in promoting oncogenesis and shaping an immunosuppressive TME. Its role is further corroborated by the frequent concurrent dysregulation with vital oncogenic and immune-regulatory pathways across various malignancies. Consequently, these bispecific agents are intentionally engineered to overcome key limitations associated with single-target therapies. Specifically, by simultaneously modulating complementary signaling pathways, they mitigate therapeutic resistance and suboptimal response rates that impede monotherapies, while also circumventing drug-drug interactions and heterogeneous pharmacokinetic discrepancies of traditional combination treatments. This approach reflects the growing consensus that synergistic dual-targeting strategies provide a more effective means of addressing the biological complexity of tumors and improving clinical outcomes (**Figure 4**).

Given the suboptimal clinical efficacy of PD-1/PD-L1 monotherapies and the well-documented mechanistic synergy between EZH2 and PD-L1 in orchestrating the immunosuppressive TME, the development of bifunctional inhibitors co-modulating the PD-L1/PD-1 and EZH2 pathways holds substantial promise for advancing cancer immunotherapy. Relative to conventional drug combination regimens, dual-target agents confer distinct advantages, including robust synergistic antitumor activity and more predictable pharmacokinetic/pharmacodynamic (PK/PD) profiles. These attributes effectively circumvent potential drug-drug interactions and inconsistent systemic exposure that may compromise therapeutic outcomes. Guided by the premise that PD-L1 and EZH2 are pivotal regulators of the immunosuppressive tumor microenvironment, we developed and synthesized a series of bifunctional small-molecule inhibitors that simultaneously target both PD-L1 and EZH2. The following sections provide a detailed account of their design, synthesis, and biological evaluation.

## 2 Materials and methods

### 2.1 Chemical general methods

All reagents and solvents were purchased from commercial suppliers and used without further purification unless otherwise specified. BMS-202 and Tazemetostat were purchased from *InvivoChem*. Reactions were tracked using Thin Layer Chromatography (TLC) on silica gel GF/UV 254. Flash column chromatography employed 300–400 mesh silica gel. NMR spectra (^1^H and ^13^C) were recorded on a Bruker AV-400 spectrometer with Tetramethylsilane (TMS) as the internal standard. Compound purity was confirmed to be over 95% through HPLC analysis.

### 2.2 Characterization and analytical test results of the target compounds

*N*-(2-(((2-methoxy-6-(2-methyl-[1,1’-biphenyl]-3-yl)pyridin-3-yl)methyl)amino)ethyl)-4,6-dimethyl-2-oxo-1,2-dihydropyridine-3-carboxamide (PE-1). ^1^H NMR (400 MHz, *d*_6_-DMSO) δ 9.24 (s, 1H), 7.84 (d, *J* = 7.1 Hz, 1H), 7.46 (m, 2H), 7.36 (m, 5H), 7.24 (d, *J* = 7.2 Hz, 1H), 7.11 (d, *J* = 7.3 Hz, 1H), 6.01 (s, 1H), 3.90 (s, 3H), 3.77 (s, 2H), 3.18 (s, 2H), 2.74 (d, *J* = 5.9 Hz, 2H), 2.33 (s, 3H), 2.17 (s, 6H). ^13^C NMR (101 MHz, *d*_6_-DMSO) δ 166.30, 162.68, 160.62, 155.67, 154.27, 146.24, 142.92, 142.04, 141.22, 138.12, 133.20, 129.57, 129.16, 128.63, 127.37, 125.93, 119.44, 117.39, 109.61, 53.53, 48.57, 46.73, 38.70, 21.67, 18.77, 18.62. MS m/z: calcd for C_30_H_32_N_4_O_3_ 496.24, found 497.35 [M + H]^+^. HPLC *t*_R_ 3.177 min, purity 98.51%.

3-((((2-methoxy-6-(2-methyl-[1,1’-biphenyl]-3-yl)pyridin-3-yl)methyl)amino)methyl)-4,6-dimethylpyridin-2(1*H*)-one (PE-2). ^1^H NMR (400 MHz, *d*_6_-DMSO) δ 7.94 (d, *J* = 7.4 Hz, 1H), 7.46 (t, *J* = 7.3 Hz, 2H), 7.41 – 7.31 (m, 5H), 7.25 (d, *J* = 7.1 Hz, 1H), 7.17 (d, *J* = 7.4 Hz, 1H), 5.97 (s, 1H), 4.12 (s, 2H), 3.94 (d, *J* = 8.3 Hz, 5H), 2.16 (d, *J* = 7.7 Hz, 9H), 1.91 (s, 1H). ^13^C NMR (101 MHz, *d*_6_-DMSO) δ 163.60, 160.90, 157.83, 151.90, 144.98, 143.00, 141.90, 140.79, 140.68, 133.19, 130.14, 129.55, 129.14, 128.65, 127.42, 126.02, 117.54, 117.18, 114.64, 108.63, 53.90, 45.65, 43.97, 19.28, 18.73, 18.65. MS m/z: calcd for C_28_H_29_N_3_O_2_ 439.22, found 440.30 [M + H]^+^. HPLC *t*_R_ 2.702 min, purity 96.90%.

*N*-(5-(1-(3-bromo-4-((2-methyl-[1,1’-biphenyl]-3-yl)methoxy)benzyl)piperidine-2-carboxamido)pentyl)-4,6-dimethyl-2-oxo-1,2-dihydropyridine-3-carboxamide (PE-3). ^1^H NMR (400 MHz, *d*_6_-DMSO) δ 11.90 (s, 1H), 9.20 (t, *J* = 5.3 Hz, 1H), 7.84 (t, *J* = 5.7 Hz, 1H), 7.59 (s, 1H), 7.53 (d, *J* = 7.5 Hz, 1H), 7.46 (t, *J* = 7.5 Hz, 2H), 7.39 (d, *J* = 7.1 Hz, 1H), 7.30 (m, 4H), 7.22 (dd, *J* = 12.5, 8.0 Hz, 2H), 5.98 (s, 1H), 5.22 (s, 2H), 3.64 (d, *J* = 13.2 Hz, 1H), 3.14 (m, 3H), 3.04 (d, *J* = 13.1 Hz, 2H), 2.75 (d, *J* = 11.4 Hz, 1H), 2.67 (m, 1H), 2.31 (s, 3H), 2.22 (s, 3H), 2.16 (s, 3H), 1.88 (t, *J* = 10.7 Hz, 1H), 1.67 (dd, *J* = 24.8, 12.5 Hz, 2H), 1.54 (d, *J* = 12.2 Hz, 2H), 1.44 (m, 3H), 1.36 (d, *J* = 11.8 Hz, 1H), 1.28 (m, 4H).^13^C NMR (101 MHz, *d*_6_-DMSO) δ 173.44, 165.73, 162.75, 154.64, 153.87, 146.28, 142.52, 141.76, 135.76, 134.15, 133.55, 132.84, 129.98, 129.82, 129.48, 128.60, 127.82, 127.15, 125.82, 119.03, 113.98, 111.45, 109.57, 69.68, 67.69, 58.62, 51.32, 43.64, 38.78, 38.44, 29.06, 24.87, 24.36, 23.76, 21.72, 18.60, 16.36. MS m/z: calcd for C_40_H_47_BrN_4_O_4_ 726.27, found 727.50 [M + H]^+^. HPLC *t*_R_ 2.724 min, purity 99.12%.

1-(3-bromo-4-((2-methyl-[1,1’-biphenyl]-3-yl)methoxy)benzyl)-*N*-(7-(((4,6-dimethyl-2-oxo-1,2-dihydropyridin-3-yl)methyl)amino)-7-oxoheptyl)piperidine-2-carboxamide (PE-4). ^1^H NMR (400 MHz, *d*_6_-DMSO) δ 11.46 (s, 1H), 7.83 (t, *J* = 5.3 Hz, 1H), 7.64 (d, *J* = 4.6 Hz, 1H), 7.60 (s, 1H), 7.53 (d, *J* = 7.5 Hz, 1H), 7.46 (t, *J* = 7.3 Hz, 2H), 7.38 (t, *J* = 7.2 Hz, 1H), 7.31 (d, *J* = 7.9 Hz, 4H), 7.22 (dd, *J* = 13.9, 8.0 Hz, 2H), 5.80 (s, 1H), 5.22 (s, 2H), 4.06 (d, *J* = 4.8 Hz, 2H), 3.64 (d, *J* = 13.3 Hz, 1H), 3.14 (m, 1H), 3.02 (dd, *J* = 16.0, 8.3 Hz, 2H), 2.71 (dd, *J* = 28.4, 10.1 Hz, 2H), 2.23 (s, 3H), 2.09 (s, 6H), 2.01 (t, *J* = 7.2 Hz, 2H), 1.87 (t, *J* = 10.6 Hz, 1H), 1.68 (m, 2H), 1.52 (m, 2H), 1.39 (d, *J* = 7.3 Hz, 5H), 1.19 (s, 5H).^13^C NMR (101 MHz, *d*_6_-DMSO) δ 173.30, 172.18, 163.44, 153.84, 149.66, 143.06, 142.52, 141.71, 135.72, 134.15, 133.54, 129.57, 128.64, 127.90, 127.35, 125.91, 122.48, 114.01, 111.35, 107.74, 69.67, 67.80, 58.57, 51.42, 38.56, 35.44, 34.66, 30.18, 29.56, 28.79, 26.51, 25.72, 25.03, 23.59, 19.17, 18.62, 16.33. MS m/z: calcd for C_42_H_51_BrN_4_O_4_ 754.30, found 755.50 [M + H]^+^. HPLC *t*_R_ 1.601 min, purity 98.29%.

*N*-(2-(((2-methoxy-6-((2-methyl-[1,1’-biphenyl]-3-yl)methoxy)pyridin-3-yl)methyl)amino)ethyl)-4,6-dimethyl-2-oxo-1,2-dihydropyridine-3-carboxamide (PE-5). ^1^H NMR (400 MHz, *d*_6_-DMSO) δ 9.27 (s, 1H), 7.73 (d, *J* = 7.5 Hz, 1H), 7.32 (m, 9H), 6.45 (d, *J* = 7.5 Hz, 1H), 6.02 (s, 1H), 5.42 (s, 2H), 3.90 (s, 3H), 3.79 (s, 2H), 3.49 (s, 2H), 2.80 (s, 2H), 2.31 (s, 3H), 2.19 (d, *J* = 14.9 Hz, 6H).^13^C NMR (101 MHz, *d*_6_-DMSO) δ 172.45, 166.45, 162.74, 154.45, 146.50, 142.61, 142.55, 141.79, 136.24, 134.22, 129.93, 129.54, 128.64, 127.34, 125.87, 109.72, 101.40, 66.77, 53.86, 47.72, 45.60, 37.43, 21.61, 18.64, 16.31. MS m/z: calcd for C_31_H_34_N_4_O_4_ 526.25, found 527.40 [M + H]^+^. HPLC *t*_R_ 3.185 min, purity 97.96%.

3-((4-chloro-5-((3-(2,3-dihydrobenzo[*b*] (1, 4)dioxin-6-yl)-2-methylbenzyl)oxy)-2-((((4,6-dimethyl-2-oxo-1,2-dihydropyridin-3-yl)methyl)amino)methyl)phenoxy)methyl)benzonitrile (PE-6). ^1^H NMR (400 MHz, *d*_6_-DMSO) δ 8.01 (s, 1H), 7.88 (d, *J* = 7.7 Hz, 1H), 7.84 (d, *J* = 7.6 Hz, 1H), 7.64 (t, *J* = 7.6 Hz, 1H), 7.56 (s, 1H), 7.45 (d, *J* = 7.2 Hz, 1H), 7.25 (t, *J* = 7.4 Hz, 1H), 7.18 (d, *J* = 7.5 Hz, 1H), 7.14 (s, 1H), 6.93 (d, *J* = 8.1 Hz, 1H), 6.80 – 6.71 (m, 2H), 5.95 (s, 1H), 5.33 (s, 2H), 5.27 (s, 2H), 4.28 (s, 4H), 4.06 (s, 2H), 3.85 (s, 2H), 2.25 (s, 3H), 2.14 (s, 3H), 2.09 (s, 3H), 1.91 (s, 1H). ^13^C NMR (101 MHz, *d*_6_-DMSO) δ 163.50, 156.59, 155.12, 151.86, 145.01, 143.38, 142.94, 142.10, 138.60, 135.23, 134.75, 134.50, 132.84, 132.24, 131.44, 130.20, 128.05, 125.90, 122.52, 119.11, 118.11, 117.23, 113.30, 111.96, 108.60, 100.59, 70.12, 69.49, 64.52, 45.16, 43.63, 19.19, 18.65, 16.30. MS m/z: calcd for C_39_H_36_ClN_3_O_5_ 661.23, found 662.30 [M + H]^+^. HPLC *t*_R_ 3.087 min, purity 96.17%.

3-((4-chloro-2-((((4,6-dimethyl-2-oxo-1,2-dihydropyridin-3-yl)methyl)amino)methyl)-5-((2-methyl-[1,1’-biphenyl]-3-yl)methoxy)phenoxy)methyl)benzonitrile (PE-7). ^1^H NMR (400 MHz, *d*_6_-DMSO) δ 8.00 (s, 1H), 7.86 (dd, *J* = 12.7, 7.9 Hz, 2H), 7.63 (t, *J* = 7.7 Hz, 1H), 7.54 (s, 1H), 7.46 (d, *J* = 7.7 Hz, 3H), 7.40 (d, *J* = 7.2 Hz, 1H), 7.34 – 7.27 (m, 3H), 7.22 (d, *J* = 7.3 Hz, 1H), 7.13 (s, 1H), 5.94 (s, 1H), 5.30 (d, *J* = 11.1 Hz, 4H), 4.00 (s, 2H), 3.80 (s, 2H), 2.24 (s, 3H), 2.13 (s, 3H), 2.08 (s, 3H), 1.91 (s, 2H). ^13^C NMR (101 MHz, *d*_6_-DMSO) δ 163.54, 156.47, 154.85, 144.66, 142.64, 141.65, 138.68, 135.34, 134.40, 132.81, 132.22, 131.92, 131.41, 130.21, 129.56, 128.68, 128.28, 127.41, 125.99, 119.10, 113.31, 111.96, 108.56, 100.65, 70.10, 69.45, 45.39, 43.76, 19.16, 18.63, 16.27. MS m/z: calcd for C_37_H_34_ClN_3_O_3_ 603.22, found 604.30 [M + H]^+^. HPLC *t*_R_ 3.194 min, purity 95.43%.

3-((((2-methoxy-6-((2-methyl-[1,1’-biphenyl]-3-yl)methoxy)pyridin-3-yl)methyl)amino)methyl)-4,6-dimethylpyridin-2(1*H*)-one (PE-8). ^1^H NMR (400 MHz, *d*_6_-DMSO) δ 7.80 (d, *J* = 8.0 Hz, 1H), 7.45 (t, *J* = 6.9 Hz, 3H), 7.39 (d, *J* = 7.1 Hz, 1H), 7.30 (d, *J* = 7.3 Hz, 2H), 7.26 (d, *J* = 7.5 Hz, 1H), 7.18 (d, *J* = 7.4 Hz, 1H), 6.48 (d, *J* = 8.0 Hz, 1H), 5.96 (s, 1H), 5.43 (s, 2H), 3.97 (s, 2H), 3.93 (s, 3H), 3.84 (s, 2H), 2.22 (s, 3H), 2.15 (s, 6H), 1.91 (s, 1H). ^13^C NMR (101 MHz, *d*_6_-DMSO) δ 163.51, 162.55, 160.73, 143.68, 142.58, 141.77, 136.08, 134.27, 130.00, 129.54, 128.65, 127.36, 125.89, 108.52, 101.65, 66.82, 53.94, 45.00, 43.37, 19.33, 18.65, 16.33. MS m/z: calcd for C_29_H_31_N_3_O_3_ 469.23, found 470.30 [M + H]^+^. HPLC *t*_R_ 2.926 min, purity 95.69%.

2-((5-chloro-2-((3-cyanobenzyl)oxy)-4-((2-methyl-[1,1’-biphenyl]-3-yl)methoxy)benzyl)amino)-*N*-((4,6-dimethyl-2-oxo-1,2-dihydropyridin-3-yl)methyl)-2-methylpropanamide (PE-9). ^1^H NMR (400 MHz, *d*_6_-DMSO) δ 11.43 (s, 1H), 7.93 (s, 1H), 7.80 (d, *J* = 7.5 Hz, 3H), 7.61 (d, *J* = 7.7 Hz, 1H), 7.46 (d, *J* = 7.5 Hz, 3H), 7.39 (s, 1H), 7.31 (d, *J* = 7.6 Hz, 3H), 7.26 (d, *J* = 7.5 Hz, 1H), 7.20 (d, *J* = 7.6 Hz, 1H), 7.05 (s, 1H), 5.78 (s, 1H), 5.30 (s, 2H), 5.24 (s, 2H), 4.12 (d, *J* = 4.4 Hz, 2H), 2.23 (s, 3H), 2.10 (s, 3H), 2.06 (s, 3H), 1.21 (d, *J* = 19.0 Hz, 6H). ^13^C NMR (101 MHz, *d*_6_-DMSO) δ 163.52, 143.21, 142.63, 141.73, 135.58, 134.41, 132.58, 132.07, 131.19, 130.16, 129.61, 128.72, 128.28, 127.45, 125.98, 119.10, 113.32, 111.90, 107.87, 100.98, 70.07, 69.14, 29.47, 25.88, 19.23, 18.63, 16.30. MS m/z: calcd for C_41_H_41_ClN_4_O_4_ 688.28, found 689.30 [M + H]^+^. HPLC *t*_R_ 3.299 min, purity 97.93%.

1-(3-bromo-4-((2-methyl-[1,1’-biphenyl]-3-yl)methoxy)benzyl)-*N*-((4,6-dimethyl-2-oxo-1,2-dihydropyridin-3-yl)methyl)piperidine-2-carboxamide (PE-10). ^1^H NMR (400 MHz, *d*_6_-DMSO) δ 11.54 (s, 1H), 7.67 (s, 1H), 7.53 (d, *J* = 7.4 Hz, 1H), 7.49 – 7.43 (m, 3H), 7.39 (d, *J* = 7.1 Hz, 1H), 7.35 – 7.27 (m, 4H), 7.22 (t, *J* = 8.5 Hz, 3H), 5.81 (s, 1H), 5.22 (s, 2H), 4.32 – 4.23 (m, 1H), 4.09 (d, *J* = 11.8 Hz, 1H), 3.57 (d, *J* = 12.1 Hz, 1H), 2.98 (d, *J* = 11.6 Hz, 1H), 2.69 (s, 2H), 2.23 (s, 3H), 2.14 (s, 3H), 2.08 (s, 3H), 1.87 (s, 1H), 1.72 (d, *J* = 5.3 Hz, 1H), 1.62 (d, *J* = 9.5 Hz, 1H), 1.52 – 1.44 (m, 2H), 1.32 (d, *J* = 7.0 Hz, 1H). ^13^C NMR (101 MHz, *d*_6_-DMSO) δ 163.59, 143.25, 142.53, 141.70, 135.68, 134.12, 130.00, 129.57, 128.65, 127.85, 127.37, 125.94, 113.99, 111.21, 107.85, 69.66, 58.64, 51.02, 34.72, 19.22, 18.58, 16.21. MS m/z: calcd for C_35_H_38_BrN_3_O_3_ 627.21, found 628.25 [M + H]^+^. HPLC *t*_R_ 3.039 min, purity 99.23%.

### 2.3 Molecular dynamics simulations

Target protein structures were obtained from the Protein Data Bank (PDB). Protein–ligand complexes were generated by molecular docking with AutoDock Vina. The resulting complexes were subjected to molecular dynamics simulations using the GROMACS 2019 software package and the CHARMM36 force field. Each system was placed in a triclinic simulation box with a minimum distance of 1.0 nm between the solute and the box boundary. The system was solvated with TIP3P water molecules, and counterions were added to neutralize the net charge and maintain a physiological ionic strength of 0.15 M. Periodic boundary conditions were applied in all three dimensions. Energy minimization was performed using the steepest descent algorithm to eliminate steric clashes and optimize the initial configuration. Equilibration was performed in two successive steps: 100 ps under the canonical (NVT) ensemble, followed by 100 ps under the isothermal–isobaric (NPT) ensemble. The temperature was maintained at 310.15 K using the Berendsen thermostat, and the pressure was held at 1 bar with the Berendsen barostat. Production MD simulations were run for 100 ns using the leap-frog integrator with a 2fs time step. Long-range electrostatic interactions were computed via the Particle Mesh Ewald (PME) method, and van der Waals interactions were treated with the Lennard-Jones potential. Covalent bonds were constrained using the LINCS algorithm. Trajectory analyses included root-mean-square deviation (RMSD), root-mean-square fluctuation (RMSF), radius of gyration (Rg), solvent-accessible surface area (SASA), hydrogen bond analysis, interaction energy calculation, and principal component analysis (PCA). The molecular mechanics/Poisson–Boltzmann surface area (MM/PBSA) method was employed to calculate binding free energies.

### 2.4 TCGA datasets analysis

Correlation analysis: Download gene expression data for specific tumors from TCGA. Filter out samples without clinical data or from tissue adjacent to tumors. Use Python’s scipy to compute the Pearson correlation, recording the correlation coefficient (corr) and *p*-value (treating *p* < 0.0001 as < 1e-4) in the EZH2-PD-L1 figure. Use scikit-learn for linear regression to fit the regression line. Use matplotlib to create scatter plots with the regression line. Survival analysis: Download relevant clinical data from TCGA. Utilize the lifelines module along with the minimum *p*-value method to identify the optimal cutoff for patient prognosis and plot the Kaplan-Meier (K-M) curve. The *p*-value is obtained via the log-rank test. Calculate the Hazard Ratio (HR) using univariate Cox regression; HR > 1 indicates a worse prognosis with higher gene expression, with each 1-unit increase raising the risk by the HR-fold.

### 2.5 *In vitro* PD-1/PD-L1 binding assay

96-well plates (Greiner; Cat#66PL96025) were used. The PD-1/PD-L1 binding assay kit was purchased from *Cisbio* (Cat#64PD1PEG). Compounds were dissolved into 20 mM stock solutions in 100% DMSO (Sigma; Cat#D8418) and then serially diluted from 10 μM in a 3-fold gradient using dilution buffer. Briefly, compounds were transferred to the assay plate in 100% DMSO, yielding a final DMSO concentration below 0.5% during the assay (20 µL final volume). 4 µL of Tag1-PD-L1, 4 µL of Tag-PD-1 solutions, and 2 µL of compound/antibody or diluent were added to the assay plate. The plate was incubated at room temperature (RT) for 15 minutes. Subsequently, 10 µL of pre-mixed anti-Tag1-Eu3+ and anti-Tag2-XL665 in detection buffer was added, followed by another 2-hour incubation at RT. Remove the plate sealer and read on an HTRF^®^-compatible reader. The plate was read on an Envision instrument (excitation 320 nm, emission at 620 nm and 665 nm). The emission Ratio (ER) was calculated as ER = Em665/Em620 × 10^4^. The inhibition rate (%) was determined by the formula: [(ER_vehicle_- ER_compound_)/(ER_vehicle_- ER_blank_)] × 100%.

### 2.6 *In vitro* assay for EZH2 enzymatic inhibition

EZH2 enzymatic activity was determined using the commercially available MTase-Glo™ Methyltransferase Assay Kit (*Promega*, Cat. No. V7601). Briefly, reactions were performed in white, opaque 384-well microplates. Each well was sequentially supplemented with 1× assay buffer, recombinant human EZH2-PRC2 complex, the methyl donor *S*-adenosylmethionine (SAM), and a histone H3-derived substrate peptide. The plate was gently mixed and briefly centrifuged to ensure all reagents settled to the bottom of each well. Tenfold serial dilutions of the test compounds were then added, with the final DMSO concentration kept below 0.5%. Blank control (without test compound), negative control (without EZH2), and positive control wells were included in parallel. The plate was sealed and incubated at 30°C in the dark for 60 min to allow EZH2-catalyzed methylation of the substrate peptide, which generates S-adenosylhomocysteine (SAH) as a byproduct. Following incubation, MTase-Glo™ reagent was added to convert SAH into adenosine diphosphate (ADP). After a room-temperature dark reaction, MTase-Glo™ detection solution was added to convert ADP into adenosine triphosphate (ATP), which drives a luciferase-mediated chemiluminescent reaction. Luminescence intensity was measured using a multi-mode microplate reader. The inhibition rate was calculated as: Inhibition rate (%) = (1 − Luminescence of test group/Luminescence of negative control group) × 100.

### 2.7 BLI binding kinetics assay

Human PD-L1 (hPD-L1), murine PD-L1 (mPD-L1), and human EZH2 (hEZH2) proteins were purchased from Sino Biological Inc. (catalog numbers 10084-H05H, 50010-M02H, and E396-30G, respectively). Each protein was dissolved in phosphate-buffered saline (PBS) at 100 μg/mL and biotinylated using EZ-Link NHS-LC-Biotin. Streptavidin (SA) biosensors were pre-wetted with PBS to establish a stable baseline, then the biotinylated protein was immobilized onto the SA biosensors in black-bottom 96-well plates (Corning, Cat. No. 3915). Test compounds were serially diluted in PBS supplemented with 0.1% DMSO and 0.02% Tween 20 to the desired concentrations, with a final volume of 200 μL per well. An equal volume of PBS containing 15% DMSO and 0.02% Tween 20 served as the vehicle control. The assay was performed in a cyclic manner consisting of four sequential steps: sensor loading (300 s), baseline stabilization (60 s), analyte association (120 s), and dissociation (120 s). Binding kinetics data were collected and analyzed using the Octet R8 Data Acquisition and Analysis Software (Sartorius, Göttingen, Germany).

### 2.8 Cell cycle analysis, apoptosis detection, and mitochondrial membrane potential assay

CT-26 cells were obtained from Wuhan Ponose Life Science and Technology Co., Ltd. Cells were seeded into 6-well plates at 5 × 10^5^ cells per well and incubated overnight to allow adherence. After 24 h of treatment with the test compound, three assays were performed. For mitochondrial membrane potential (ΔΨm) measurement, treated cells were stained with a JC-1 detection kit (KeyGEN, China) and analyzed by flow cytometry. For cell cycle analysis, cells were fixed and stained with propidium iodide (PI), and cell cycle distribution was determined using a BD FACSCanto II flow cytometer (USA). For apoptosis detection, cells were stained with an Annexin V-FITC/PI double-staining kit, and apoptotic populations were quantified by flow cytometry.

### 2.9 PD-1/PD-L1 NFAT reporter bioassay

CHO/PD-L1/TCR cells and PD-1/NFAT/Jurkat cells were purchased from Conradbio Co., Ltd. and used to evaluate the *in vitro* efficacy of PD-L1 inhibitors in a co-culture system. Briefly, CHO/PD-L1/TCR cells, which overexpress both the TCR ligand and PD-L1, and PD-1/NFAT/Jurkat cells, a modified Jurkat T-cell line stably expressing PD-1 and an NFAT promoter-driven luciferase reporter, were maintained in complete RPMI 1640 medium. Before the assay, CHO/PD-L1/TCR cells were seeded at 3.5 × 10^5^ cells/mL and incubated at 37°C with 5% CO_2_ for 24 h to allow adherence. On the day of the experiment, the culture supernatant was discarded, and the cells were treated with serially diluted test compounds. Plates were incubated at 37°C with 5% CO_2_ for 30 min, followed by the addition of PD-1/NFAT/Jurkat cells at 4.0 × 10^5^ cells/ml. After co-incubation for 5 h under the same conditions, plates were equilibrated at room temperature for 10 min, and 100 μL of Bio-Glo reagent was added to each well. Following a further 10 min incubation, luminescence was measured using a FlexStation 3 microplate reader (Molecular Devices). The half-maximal effective concentration (EC_50_) and maximum luminescence (RLUmax) were calculated by fitting the experimental data to the Hill equation.

### 2.10 Western blot analyses of H3K27me3

CT-26 cells (5 × 10^5^) were seeded in six-well plates and cultured for 24 hours, then treated with the compounds under investigation for 24 or 48 hours. Afterward, cells were washed with PBS and lysed in a buffer containing a protease inhibitor cocktail. Protein concentrations were determined by BCA assay, and samples were separated by SDS−PAGE and transferred to PVDF membranes. Membranes were blocked with 5% skim milk to reduce non-specific binding, then incubated overnight at 4°C with a monoclonal rabbit anti-H3K27me3 antibody (Cell Signaling Technology, #9733). The following day, secondary antibodies were applied at 25°C for 1 hour. Protein detection was performed using a multifunctional imaging analyzer with enhanced chemiluminescence (ECL).

### 2.11 Pharmacokinetic study in male Sprague-Dawley rats

This animal study was approved by the National Institutional Animal Care and Ethical Committee of Hubei Polytechnic University (Ethics Approval No. HBPU-IACUC-MED-2025-1615-1), and all operations complied with standard laboratory animal care specifications. Rats were raised under controlled conditions at 25°C, 50%–60% humidity, and a 12 h light-dark cycle, with unlimited access to food and water. Prior to experiments, animals were acclimatized for at least one week. Food was withheld for 12 h before sampling, while the water supply was not restricted. Trained researchers collected blood from rat tail veins using gentle techniques to reduce pain, in accordance with the 3Rs principles. Experimental male Sprague-Dawley rats (250–260 g) were purchased from CasGene Biotech. Co., Ltd. Compound PE-1 was given via three administration routes: oral gavage at 50 mg/kg, intravenous injection at 2 mg/kg, and intraperitoneal injection at 4 mg/kg. Then, 0.5 mL of blood was harvested at 0.0833, 0.25, 0.5, 1, 1.5, 2, 4, 6, 8, 12, 24, 36, and 48 h and stored in heparin-containing 1.5 mL polyethylene tubes. Sample detection was performed using an LC-MS/MS system consisting of a Shimadzu liquid chromatograph and an Applied Biosystems API 4000Q triple quadrupole mass spectrometer. Separation was performed on a C18 column (2.1 × 50 mm, 1.7 μm) at room temperature, with a flow rate of 0.4 mL/min and an injection volume of 5 μL. The binary mobile phase was 0.1% formic acid in water (A) and methanol (B). The gradient program was set as follows: 30% B initially, increased to 90% B from 1 min to 2 min, held for 1.5 min, then reduced to 5% B within 0.1 min. The whole chromatographic cycle lasted 5 min. Electrospray ionization in positive mode was adopted for mass spectrometry analysis. Key operating parameters were optimized: source temperature 550°C, spray voltage 4500 V, curtain gas 10 psi, auxiliary gases 1 and 2 at 55 psi each, and dwell time 200 ms.

### 2.12 *In vivo* efficacy study in a colon tumor model

This animal study was reviewed and approved by the National Institutional Animal Care and Ethical Committee (NIACEC) of Hubei Polytechnic University, under the ethical approval number HBPU-IACUC-MED-2025-1615-2. Animals were maintained under controlled laboratory conditions, with ambient temperature set at 25°C, relative humidity maintained between 50–60%, and a standard 12-hour light/dark cycle. All mice had unrestricted access to sterilized standard chow and drinking water throughout the study. Upon study completion, mice were humanely euthanized via cervical dislocation performed by trained personnel. Death was confirmed by continuous cessation of both heartbeat and respiration prior to any tissue collection. Female BALB/c mice aged 6–8 weeks were procured from CasGene Biotech. Co., Ltd. The total number of animals used was determined based on statistical power considerations and the experimental design, with strict adherence to the 3Rs (Replacement, Reduction, and Refinement) principles to minimize animal use and distress. Briefly, CT26 cells in logarithmic growth phase were harvested and resuspended in sterile PBS at a concentration of 1 × 10^6^ cells/mL. Each mouse received a subcutaneous inoculation of 100 µL of the cell suspension (containing 1 × 10^5^ viable CT26 cells) in the right flank, following established protocols for murine tumor transplantation models. When the tumor volume (TV) reached 50–100 mm^3^, the mice were randomly assigned to the vehicle control (n = 6) or treatment group (n = 6). PE-1 was dissolved in 20% PEG-300, 5% DMSO, and 75% saline solution. Tumor dimensions were measured using a calibrated electronic digital caliper, and tumor volume was calculated from the measured length and width. Tumor Growth Inhibition (TGI) was computed using the formula: TGI (%) = [1 − Wt/Wv] × 100%, where Wt and Wv represent the mean tumor weight of the treatment group and the vehicle control group, respectively. The harvested organs (Heart, liver, spleen, lung, and kidney) were fixed in 4% paraformaldehyde, routinely processed for paraffin, stained with hematoxylin and eosin (H&E), and imaged under a microscope.

### 2.13 Flow cytometry

Mouse organs were collected, tumor cells were mechanically isolated using a 40µm cell strainer, and erythrocytes were lysed with ACK lysis buffer (Beyotime), followed by PBS wash, centrifugation, and PBS resuspension. The cells were then stained in the dark for 30 min at 25°C. After being washed twice with PBS, cells from each group were analyzed by flow cytometry, and the data were analyzed using FlowJo (Tree Star v7.6.1). Cells were stained at 4°C for 30 min and flow cytometry (BD, USA) was performed using antibodies against the following targets (CD3, CD4, CD8) and isotype controls, all provided by BioLegend company: FITC anti-mouse CD3 Antibody (Cat No:100204, LOT: B249620), APC anti-mouse CD8a Antibody (Cat No:100711, LOT: B280032), Rat IgG2b K Isotype Control PE (Cat#:400608, Lot: B156144).

### 2.14 Statistical analysis

Data are presented as mean ± SD, *P < 0.05, **P < 0.01, ***P < 0.001, ****P < 0.0001, One-way analysis of variance (ANOVA).

## 3 Results

### 3.1 Rational design

**Figure 5:**
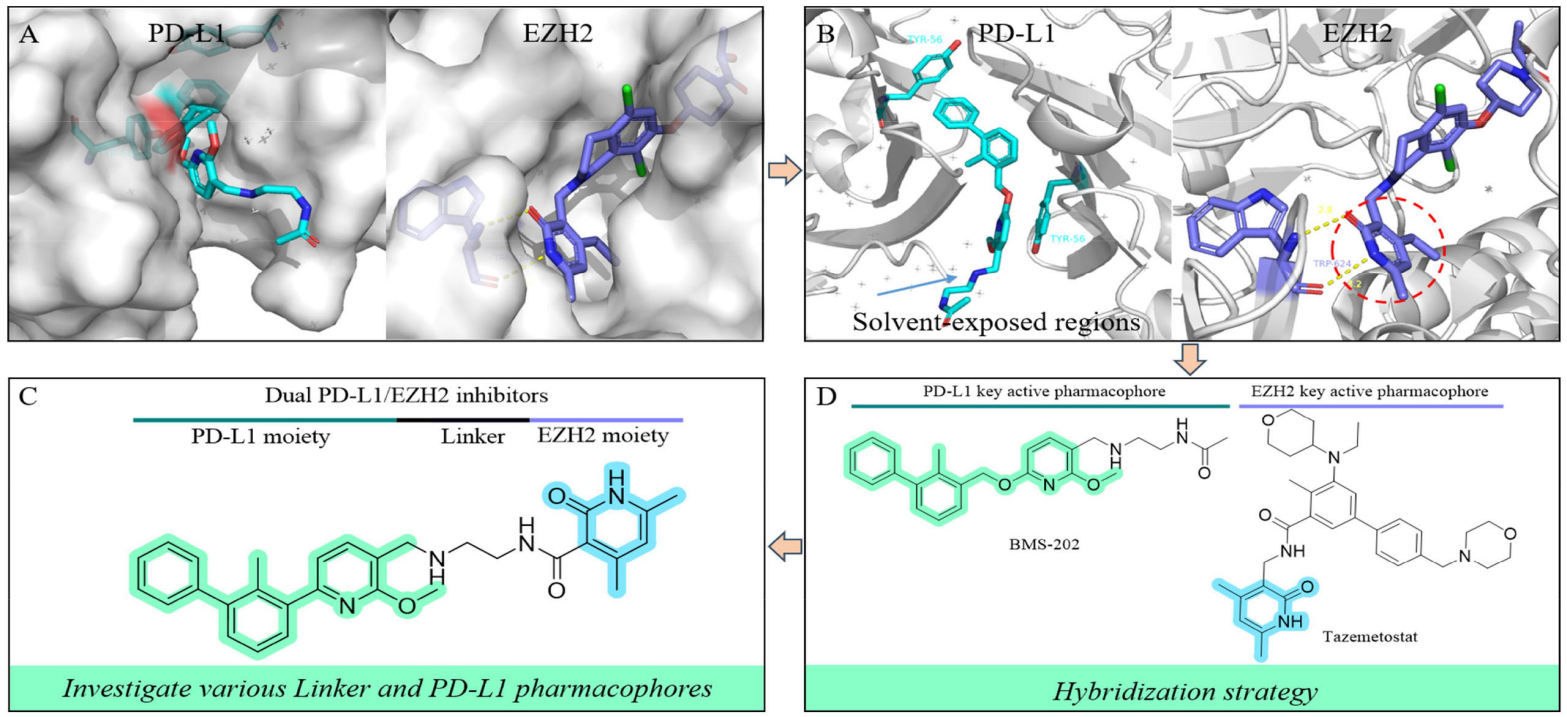
Rational design of dual-targeting PD-L1/EZH2 small-molecule inhibitors. **(A, B)** Computational docking models of BMS-202 in complex with PD-L1 (PDB: 5J89) and of an EZH2 inhibitor in complex with EZH2 (PDB: 5IJ7); **(C, D)** Molecular hybridization strategies for constructing dual-targeting PD-L1/EZH2 inhibitors.

To develop dual-target EZH2/PD-L1 inhibitors, we used a structure-guided, rational design approach informed by thorough analysis of EZH2/PD-L1 ligand-binding interactions. As shown in **Figures 5A, B**, molecular docking analysis revealed that the biphenyl scaffold of a prototypical PD-L1 inhibitor (BMS-202) fits into the hydrophobic cleft created by PD-L1 homodimerization through π-π stacking and hydrogen bonds. Structural analysis also identified a flexible side chain that extends into nearby hydrophilic cavities. Examination of the EZH2 co-crystal structure revealed that the pyridone group constitutes the essential pharmacophore for anchoring to the key catalytic domain of EZH2. This fragment stabilizes the EZH2 complex by forming two hydrogen bonds with TRP624. Drawing on these structural insights, we implemented a structure-driven molecular hybridization approach and conjugated well-validated EZH2 inhibitory motifs, such as the pyridone fragment, to solvent-accessible termini of the PD-L1-binding core scaffold. The molecules that were produced simultaneously blocked the PD-L1/PD-1 interaction and inhibited EZH2’s catalytic activity (**Figures 5C, D**). SAR investigations further confirmed that both linker flexibility and the characteristics of the PD-L1-binding moiety are essential for dual-target effectiveness, as explained below.

### 3.2 Chemical synthesis

**Scheme 1:**
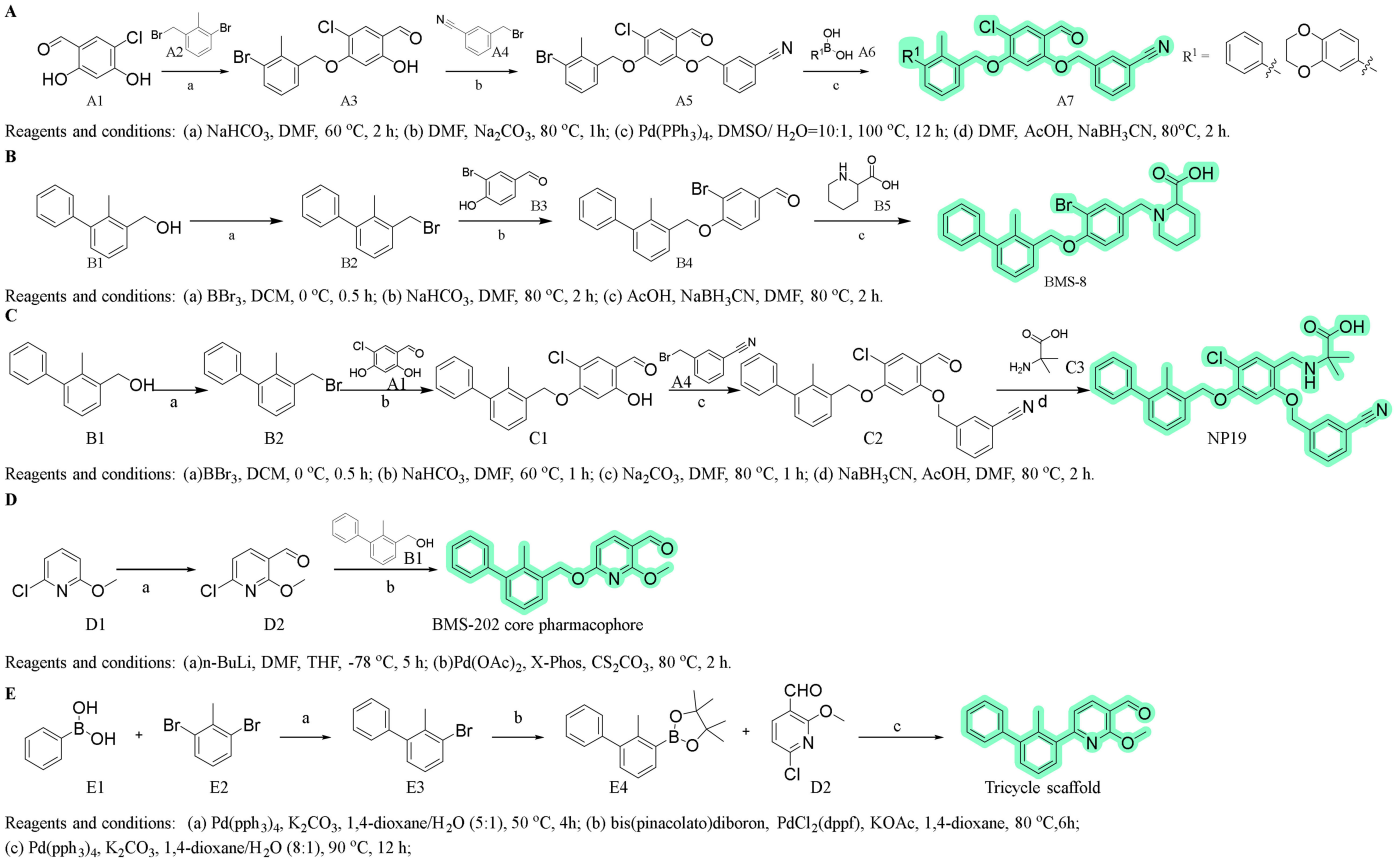
Synthetic routes for various PD-L1 binding warheads. **(A)** Synthetic route for intermediate A7; **(B)** Synthetic route for BMS-8; **(C)** Synthetic route for NP19; **(D)** Synthetic route for BMS-202 core pharmacophore; **(E)** Synthetic route for tricyclic scaffold.

**Scheme 2:**
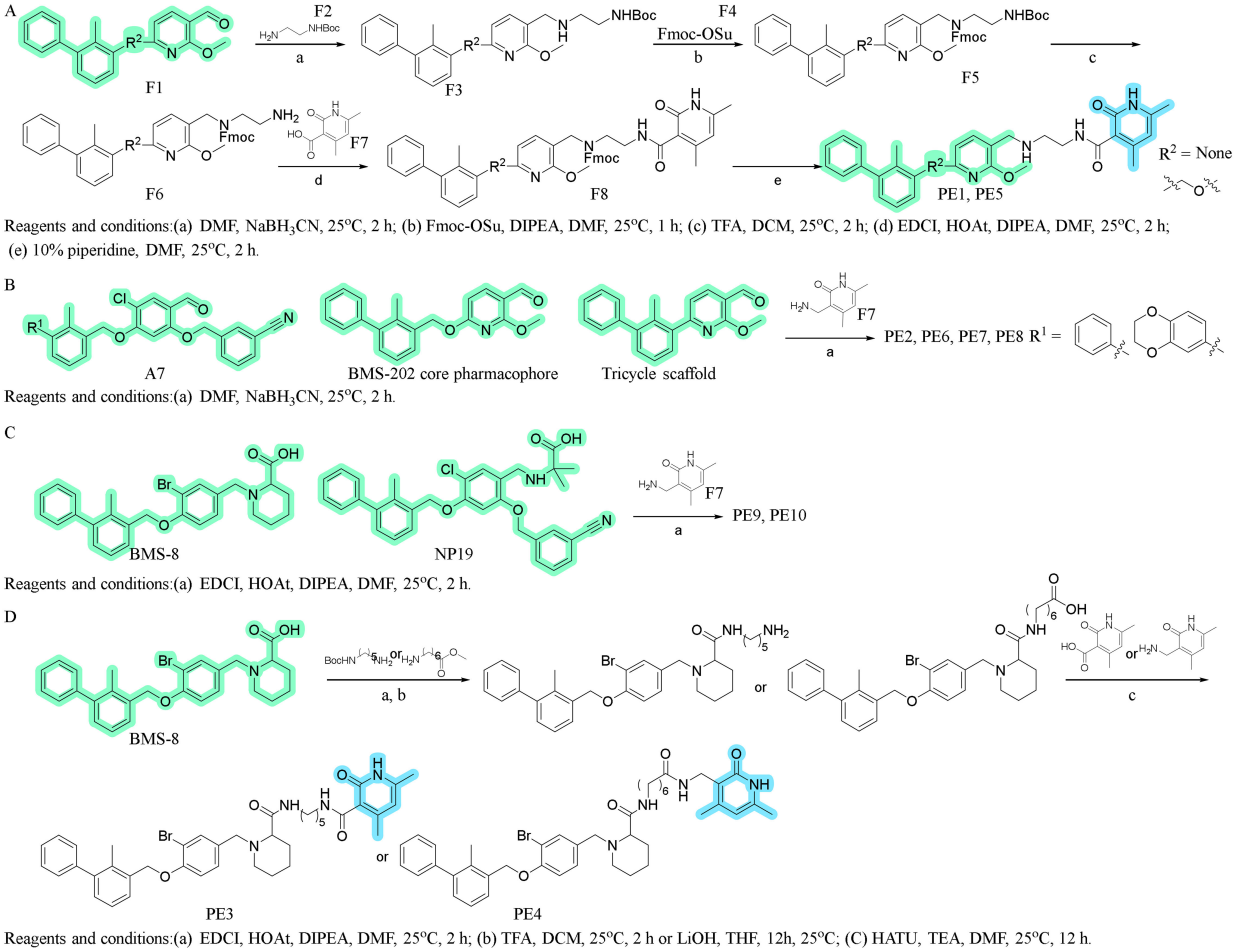
Synthetic routes for EZH2/PD-L1 bifunctional inhibitors PE1–PE10. **(A)** Synthesis of PE1 and PE5; **(B)** Synthesis of PE2, PE6, PE7 and PE8; **(C)** Synthesis of PE9 and PE10; **(D)** Synthesis of PE3 and PE4.

The synthetic routes toward the target compounds PE1–10 are outlined in **Schemes 1** and **2**. The identity and purity of the target compounds were rigorously confirmed by ¹H NMRspectroscopy; complementary characterization by ^13^C NMR, mass spectrometry (MS), and HPLCdemonstrated structural integrity and ≥95% chemical purity, meeting all predefined qualitycriteria for subsequent biological evaluation. **Scheme 1A–E** shows the synthesis of PD-L1 inhibitors with diverse chemical skeletons, including BMS-8,BMS-202, and NP19, which were synthesized via previously reported routes. The PD-L1 tricyclescaffold was taken as an illustrative example to demonstrate the synthesis process of these PD-L1warheads. As depicted in **Scheme 1E**, E3 was first synthesized from commercially available E1 and E2 using the Suzuki-Miyaura coupling reaction. Then, the intermediate E3 was converted into E5 through the Miyaura coupling reaction. Finally, a palladium-mediated cross-coupling between E4 and D2 produced the PD-L1 tricycle scaffold.

The chemical synthesis of PE1–10 is depicted in **Schemes 2A–D**. For instance, F1 was converted to the intermediate F3 via reductive amination with sodium triacetoxyborohydride. Additionally, compound F3 was protected with an Fmoc group to yield F5, and then the Boc protecting group was removed to afford F6. Next, F6 was treated with key EZH2 pharmacophores to generate intermediate F8, and finally, the Fmoc group was deprotected to yield either PE1 or PE5.

### 3.3 Dual-target inhibitors that target both EZH2 and PD-L1 demonstrate strong bifunctional inhibition (target level)

To establish the SAR of PD-L1/EZH2 dual-targeting inhibitors, we systematically evaluated how variations in the PD-L1 binding moiety, linker architecture, and EZH2 pharmacophore influence both PD-L1/PD-1 and EZH2 inhibitory activity (IC_50_), with quantitative data summarized in **Table 1**. The PD-L1 binding moiety emerged as the primary determinant of dual-target activity. Among all synthesized compounds, only PE-1 and PE-5, both bearing a biphenyl-based PD-L1 pharmacophore, exhibited measurable activity against either target. PE-1 uniquely displayed nanomolar potency against both targets: IC_50_ = 48.1 nM for PD-1/PD-L1 inhibition and IC_50_ = 101.2 nM for EZH2 inhibition. In contrast, PE-5 retained moderate EZH2 affinity (IC_50_ = 182.5 nM) but showed no detectable PD-L1/PD-1 inhibition (IC_50_ > 100 nM). All other analogs, including PE-2 to PE-4 and PE-6 to PE-10, which incorporate alternative PD-L1-based scaffolds, were inactive against both targets, with IC_50_ values exceeding 100 nM for PD-L1/PD-1 inhibition and 200 nM for EZH2 inhibition.

**Table 1:** *In vitro* PD-L1/PD-1 and EZH2 inhibitory activities of compound PE1-10.

ID	PD-L1 moiety	Linker	EZH2 moiety	IC_50_ (nM)	
				PD-l/ PD-L1	EZH2
PE-1	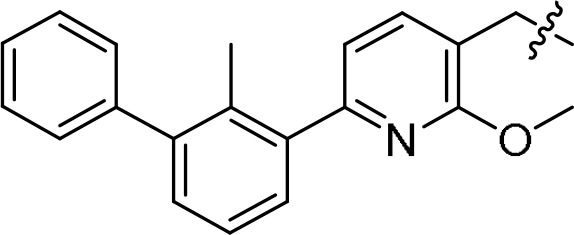	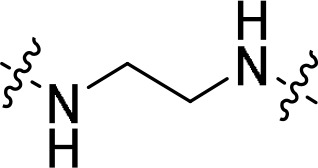	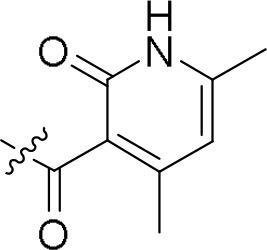	48.1 ± 2.0	101.2 ± 3.2
PE-2	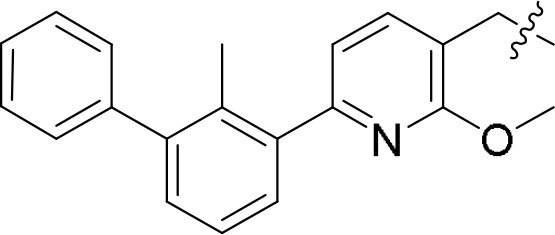	\	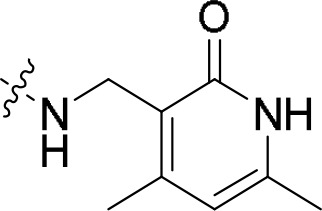	> 100	> 200
PE-3	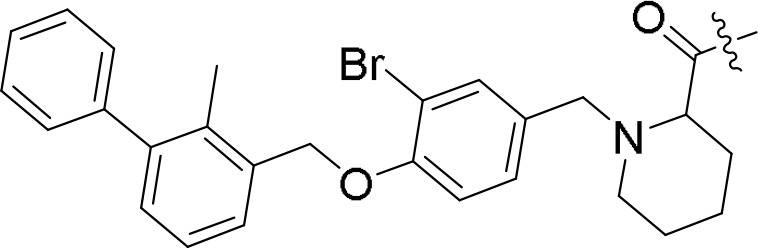	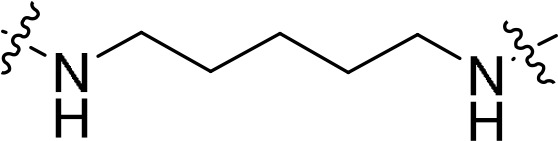	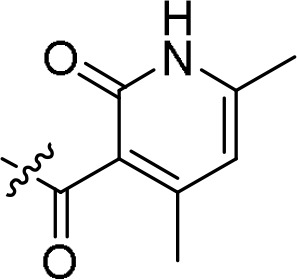	> 100	> 200
PE-4	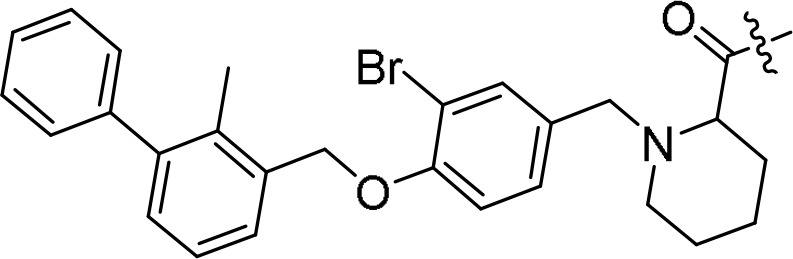	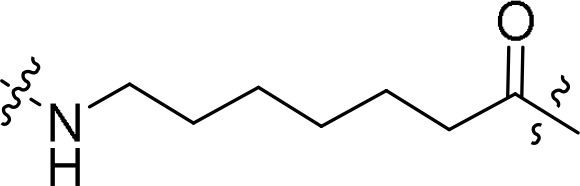	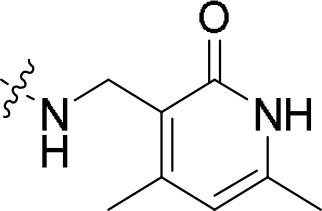	> 100	> 200
PE-5	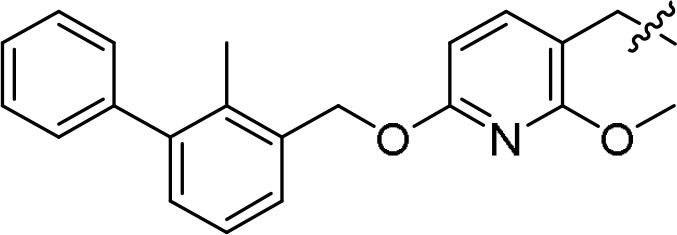	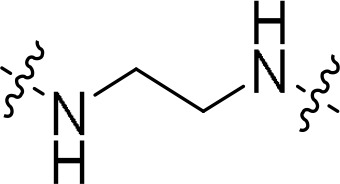	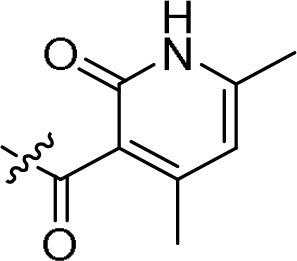	> 100	182.5 ± 5.2
PE-6	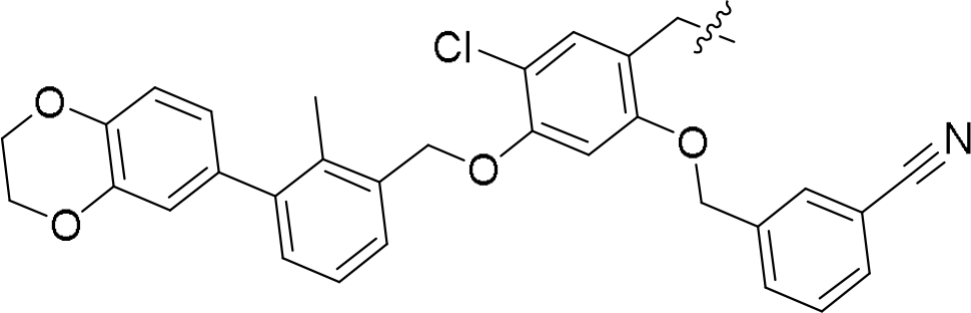	\	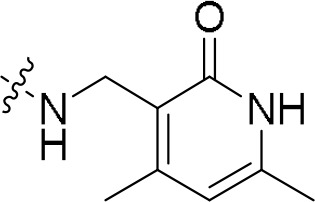	> 100	> 200
PE-7	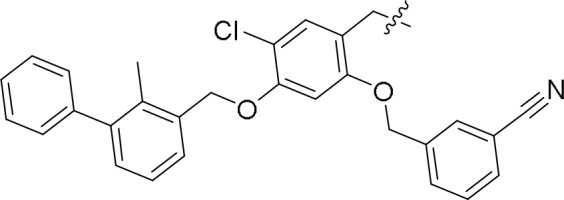	\	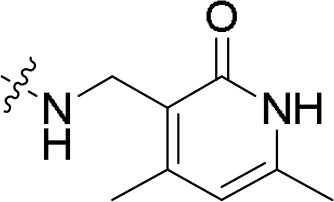	> 100	> 200
PE-8	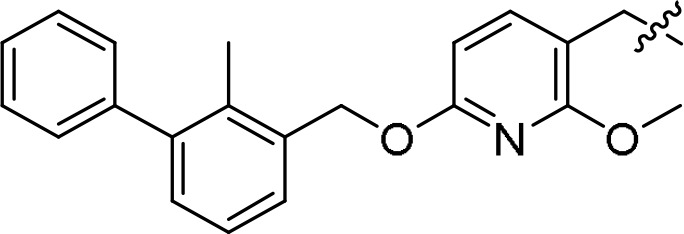	\	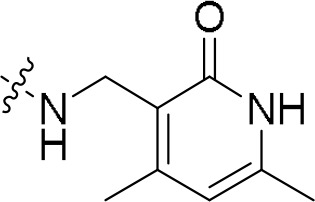	> 100	> 200
PE-9	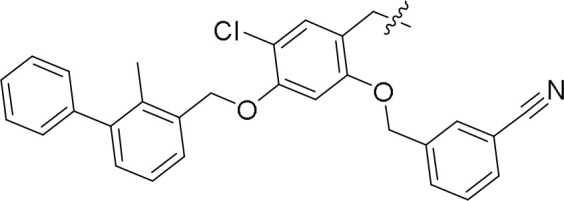	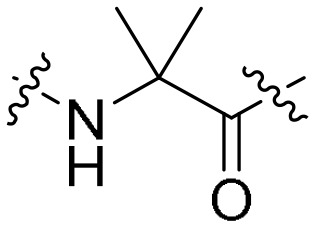	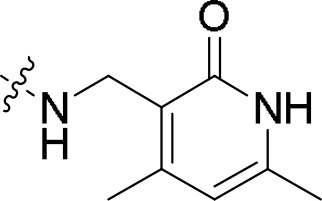	> 100	> 200
PE-10	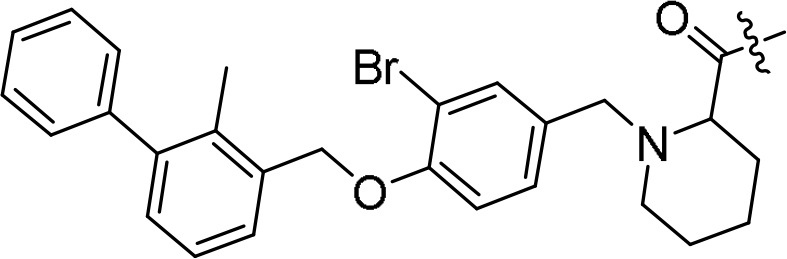	\	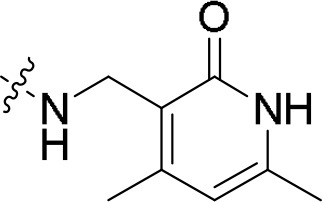	> 100	> 200
BMS-202	\	\	\	61 ± 1.9	\
Tazemetostat	\	\	\	\	0.72 ± 0.11

The linker design proved equally indispensable for facilitating dual-target engagement. The alkylamide linker in PE-1 provided optimal spatial separation and conformational compatibility, enabling concurrent targeting of PD-L1 and EZH2 while maintaining a potent dual-target profile. Conversely, linker-free analogs (PE-2, PE-6–PE-8, PE-10) demonstrated no detectable activity against either target, thereby confirming the absolute necessity of a covalent linkage across pharmacophores. Furthermore, even when present, suboptimal linkers diminished efficacy: PE-3 and PE-4, which feature extended alkyl chains, failed to attain dual-target inhibition, emphasizing that linker length and flexibility must be meticulously calibrated to accommodate the distinct binding geometries of PD-L1 and EZH2. The pyridone-based EZH2 pharmacophore was essential but not sufficient for EZH2 binding. Its activity was entirely dependent on conjugation to an active biphenyl-based PD-L1 moiety through a structurally appropriate alkylamide linker. When combined with inactive PD-L1 scaffolds (e.g., BMS-8 or NP19) or incompatible linkers, the pyridone fragment alone did not confer any measurable EZH2 inhibition, demonstrating that effective EZH2 engagement necessitates a synergistic, context-dependent presentation within the dual-pharmacophore architecture.

Notably, a direct potency comparison with the selective EZH2 inhibitor tazemetostat revealed a marked reduction in PE-1’s EZH2 inhibitory activity. Tazemetostat exhibits an IC_50_ of 0.72 nM against EZH2. This compound is approximately 140-fold more potent than PE-1, which shows an IC_50_ value of 101.2 nM. Although PE-1’s IC_50_ values against PD-L1 and EZH2 are within the same order of magnitude, its EZH2-suppressive efficacy remains substantially lower than that of clinically approved single-target EZH2 inhibitors. This potency deficit arises inherently from the structural compromises required for dual-target engagement. Incorporation of the PD-L1 binding biphenyl scaffold alters the global molecular conformation, thereby perturbing the optimal spatial alignment between the pyridone pharmacophore and the EZH2 catalytic pocket. Furthermore, the flexible alkylamide linker in PE-1 likely promotes conformational heterogeneity, reducing the population of bioactive conformers capable of deep insertion into the narrow, geometrically constrained methyltransferase active site of EZH2. Concurrently, the hydrophobic biphenyl moiety essential for PD-L1 recognition introduces steric bulk that may induce subtle but functionally relevant clashes with residues lining the EZH2 catalytic cleft. Rational structure-based optimization holds promise for narrowing this potency gap while retaining robust PD-L1 binding. Such optimization strategies include modulating linker rigidity, strategically substituting on the biphenyl ring to balance PD-L1 affinity and EZH2 accessibility, and iteratively refining the pyridone core to enhance shape complementarity with the EZH2 pocket.

Collectively, this structure-activity-relationship (SAR) investigation delineates three essential structural prerequisites governing potent PD-L1/EZH2 dual-target inhibition: (i) a biphenyl-based pharmacophore enables robust and specific target engagement with PD-L1; (ii) an alkylamide linker of defined chain length and conformational rigidity, sterically compatible binding to both targets; and (iii) precise spatial coordination and geometric matching between the two pharmacophores, as evidenced by the low overall hit rate (<10%), which substantiates the meticulous and rational design of our molecular framework and unequivocally identifies PE-1 as the leading dual-target inhibitor.

### 3.4 PE-1 showed strong binding affinities to hEZH2 and h/mPD-L1

**Figure 6:**
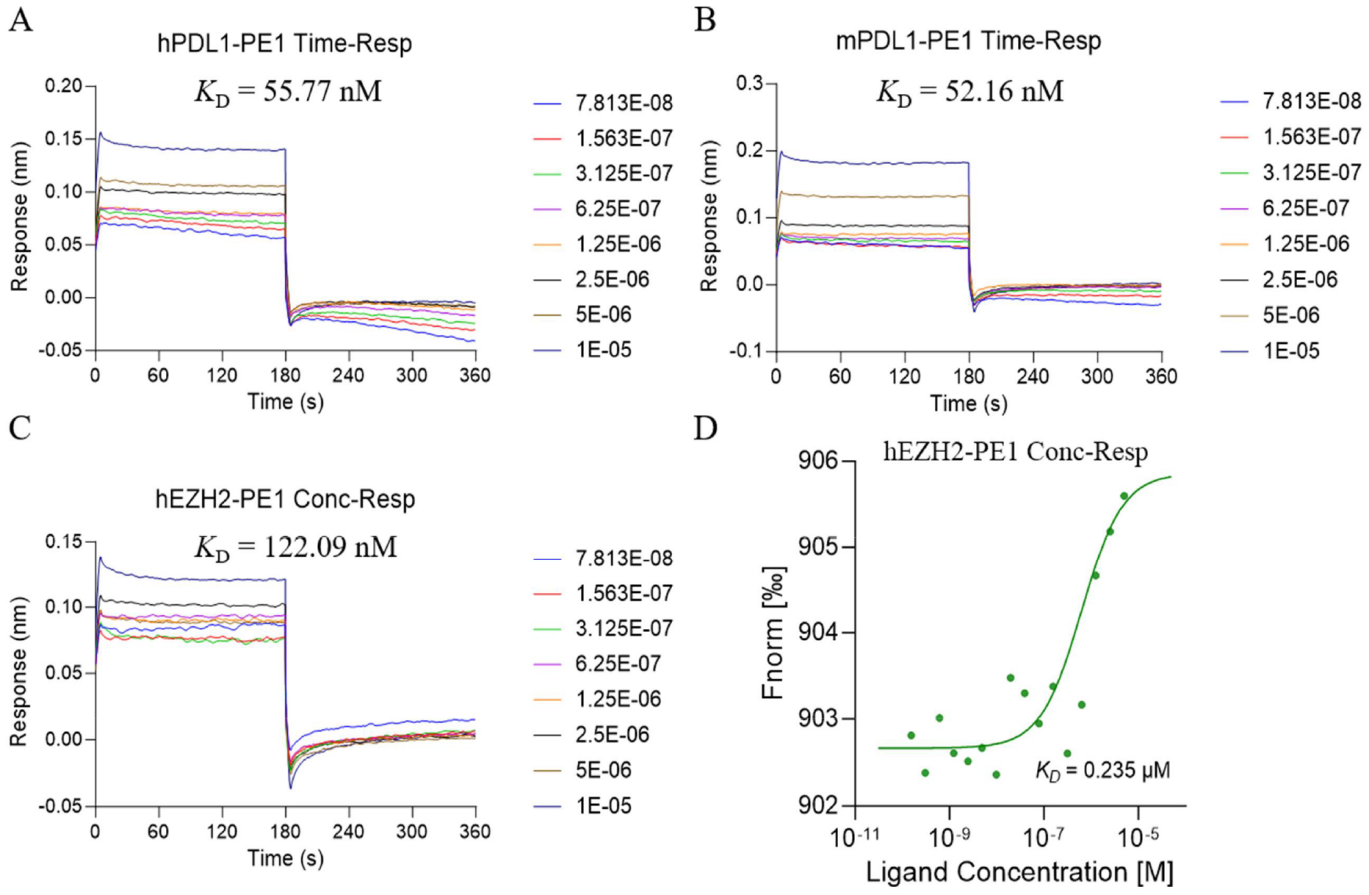
**(A–C)** Binding affinities of PE-1 to human PD-L1 (hPD-L1), murine PD-L1 (mPD-L1), and human EZH2 (hEZH2) were determined through biolayer interferometry (BLI) assays; **(D)** The fitted binding curve of PE-1 with hEZH2 was measured via MST assay.

After confirming PE-1’s dual-target inhibitory activity biochemically, we assessed its binding affinities for murine (m) and human (h) PD-L1, as well as hEZH2, using BLI and MST. As shown in **Figures 6A, B**, compound PE-1 displayed comparable binding affinity for both hPD-L1 and mPD-L1, with *K*_D_ values of 55.77 nM and 52.16 nM, respectively. To further investigate whether PE-1 exhibits cross-reactivity with hEZH2, we used BLI to quantify its binding affinity to hEZH2. As shown in **Figure 6C**, PE-1 bound to hEZH2 in a concentration-dependent manner, yielding a *K*_D_ of 122.09 nM. The substantial binding affinity of PE-1 for hEZH2 was corroborated by MST experiments. As shown in **Figure 6D**, PE-1 demonstrated potent binding to hEZH2 (*K*_D_ = 0.235 μM). Collectively, these results unequivocally indicate that PE-1 binds to both h/mPD-L1 and hEZH2 with considerable affinity.

### 3.5 Molecular dynamics simulations and molecular modeling of PE-1 with EZH2

**Figure 7:**
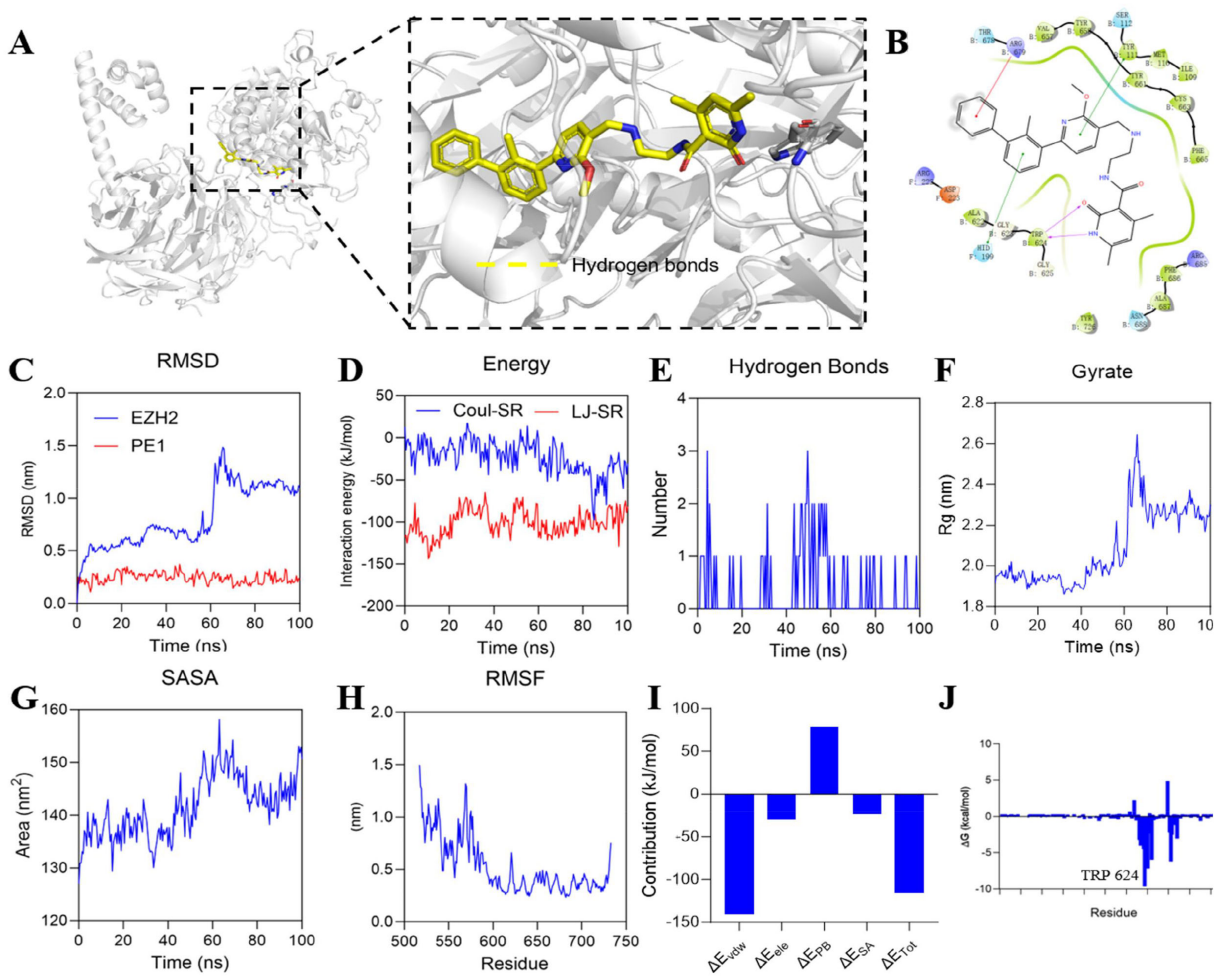
**(A)** Molecular docking model of PE-1 bound to the active site of EZH2 (PDB: 5IJ7); **(B)** Predicted binding model between PE-1 and EZH2 (PDB: 5IJ7); **(C)** RMSD of the EZH2-ligand complex system during molecular dynamics simulations; **(D)** Interaction energy of the EZH2-PE-1 complex; **(E)** Hydrogen bond interactions between PE-1 and EZH2 over the simulation period; **(F)** Radius of gyration (Gyrate) of EZH2 in the EZH2-PE-1 complex (reflecting protein conformational stability); **(G)** SASA of the EZH2-PE-1 complex; **(H)** RMSF of EZH2 residues (indicating residue flexibility in the presence of PE-1); **(I)** MM/PBSA-based binding free energy calculation results of the EZH2-PE-1 complex; **(J)** Total energy contribution of each EZH2 residue to the binding interaction with PE-1.

To characterize the docking mode and thermodynamic stability of PE-1, a bifunctional inhibitor of PD-L1 and EZH2, we performed molecular docking into the EZH2 catalytic domain using its high-resolution crystal structure (PDB ID: 5IJ7). Docking revealed that PE-1 occupies the S-adenosylmethionine (SAM)-binding pocket with high shape complementarity, yielding a favorable predicted binding energy of −6.9 kcal/mol - indicative of a secure starting binding pose (**Figure 7A**). As shown in **Figure 7B**, the biphenyl moiety engages in face-to-face π-π stacking with HID199 and TYR111, respectively, while the pyridone pharmacophore forms two key directional hydrogen bonds with TRP624.

To assess the dynamic stability of the PE-1–EZH2 complex under physiological conditions, we conducted 100 ns all-atom molecular dynamics (MD) simulations. Trajectory analysis demonstrated that the protein-ligand complex maintained structural integrity throughout the simulation: the root-mean-square deviation (RMSD) plateaued at 0.2-1.0 nm after ~60 ns of equilibration and remained stable thereafter (**Figure 7C**), indicating no large-scale conformational rearrangement upon ligand binding. Interaction energy decomposition (**Figure 7D**) revealed substantial contributions from both electrostatic (Coul-SR: 0 to −50 kJ/mol) and van der Waals (LJ-SR: consistently negative) terms. The total interaction energy remains strongly negative -consistent with synergistic stabilization mediated by hydrogen bonding and hydrophobic packing. Hydrogen-bond occupancy analysis (**Figure 7E**) showed that PE-1 maintained an average of ~2 persistent hydrogen bonds, predominantly involving the pyridone carbonyl and N-H groups, supporting specific, sustained target engagement rather than transient association.

To evaluate whether PE-1 binding elicits global conformational rearrangements in EZH2, we quantitatively characterized the solvent-accessible surface area (SASA) and radius of gyration (Rg). Rg values remained tightly constrained between 2.0 and 2.4 nm (**Figure 7F**), and SASA fluctuated narrowly within 135–150 nm^2^ (**Figure 7G**). Collectively, these observations attest to the retention of a compact, folded tertiary structure devoid of unfolding or aberrant solvent exposure. Root-mean-square fluctuation (RMSF) analysis (**Figure 7H**) further confirmed localized rigidity: residues within the binding pocket, including TRP624 and ARG679, exhibited low flexibility (RMSF < 1.0 nm), reinforcing the structural preorganization required for high-affinity inhibition.

Finally, to quantify binding affinity, we applied the MM/PBSA method to 1,000 evenly sampled frames from the production phase of the MD trajectory. The determined binding free energy (ΔG) amounted to −23.352 kcal/mol, reflecting highly favorable and thermodynamically spontaneous complex formation (**Figure 7I**). This value quantitatively aligns with the docking score and dynamic stability metrics, providing a rigorous thermodynamic basis for the experimentally observed pronounced inhibition of PE-1 by EZH2. Per-residue energy decomposition (**Figure 7J**) identified TRP624 as the dominant energetic contributor (−9.8 kcal/mol), followed by ARG679 (−6.7 kcal/mol). These findings directly validate the key interactions observed in docking and MD, establishing a consistent, multi-level structural rationale for PE-1’s dual-target efficacy.

**Figure 8:**
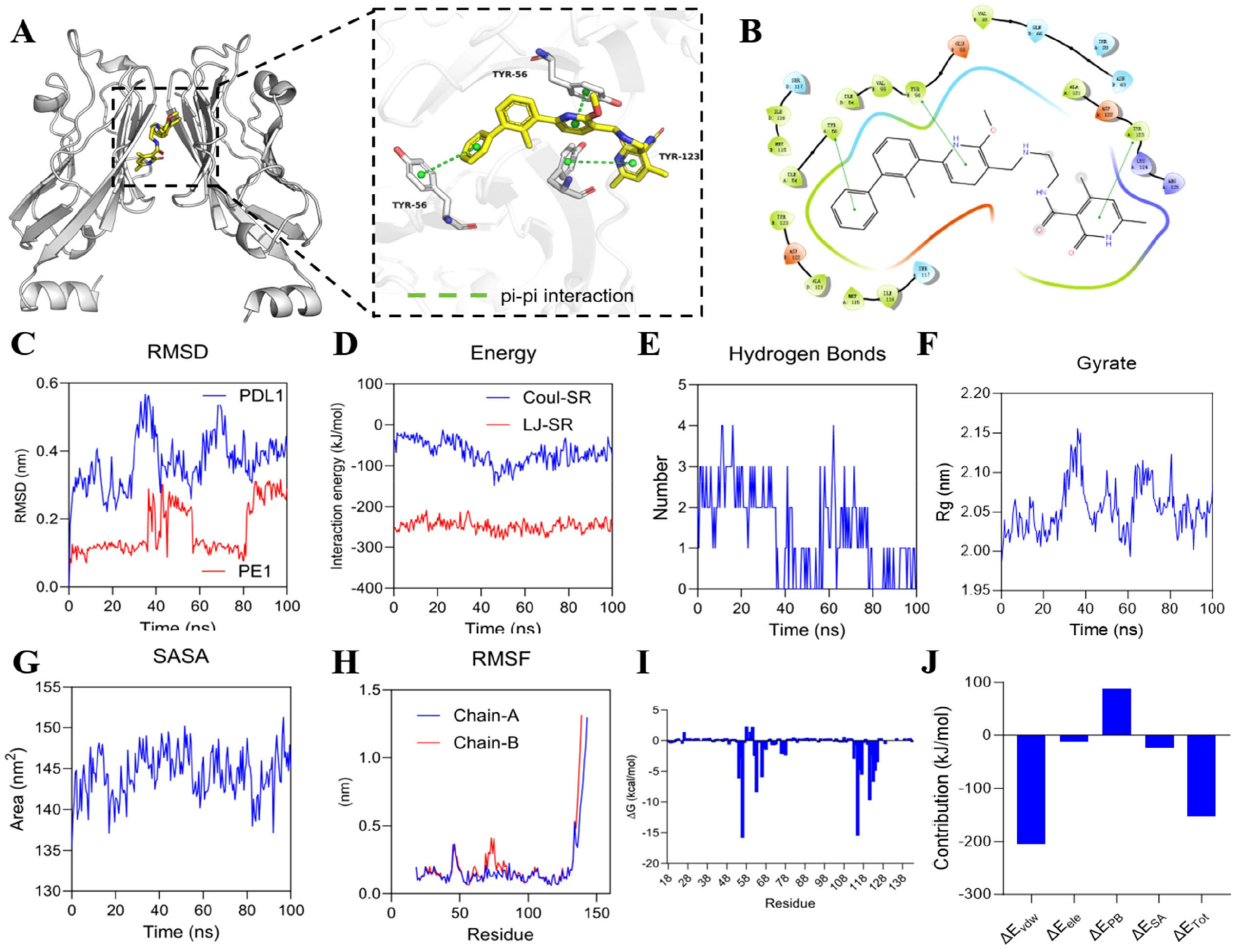
**(A)** Molecular docking model of PE-1 bound to the active site of PD-L1 (PDB: 5J89); **(B)** Predicted binding model between PE-1 and PD-L1 (PDB: 5J89); **(C)** RMSD of the PD-L1-ligand complex system during molecular dynamics simulations; **(D)** Interaction energy of the PD-L1-PE-1 complex; **(E)** Hydrogen bond interactions between PE-1 and PD-L1 over the simulation period; **(F)** Radius of gyration (Gyrate) of PD-L1 in the PD-L1-PE-1 complex, reflecting protein conformational stability; **(G)** SASA of the PD-L1-PE-1 complex; **(H)** RMSF of PD-L1 residues, indicating residue flexibility in the presence of PE-1; **(I)** Binding free energy calculation results of the PD-L1-PE-1 complex using the MM/PBSA method; **(J)** Energy contribution of each PD-L1 dimer residue to the binding interaction with PE-1.

To gain deeper insight into the binding interactions between PE-1 and the PD-L1 dimer, we used the PD-L1 crystal structure (PDB ID: 5J89) as the docking template. The docking results revealed that the hydrophobic cleft at the interface between PE-1 and the PD-L1 dimer is tightly bound, with a favorable binding energy of -6.5 kcal/mol, indicating the thermodynamic stability of the initial conformation. As depicted in **Figure 8A**, the overall binding conformation of PE-1 within the PD-L1 dimer is shown, with an inset highlighting critical π-π stacking interactions between the biphenyl group of PE-1 and the TYR56 residues of both PD-L1 chains, as well as hydrogen bonds formed with adjacent polar residues. **Figure 8B** further illustrates the comprehensive two-dimensional interaction network, confirming the spatial compatibility of the interface between the compound PE-1 and the PD-L1 dimer, as well as the specific molecular contacts that facilitate this interaction.

Next, we performed 100 ns molecular dynamics simulations to evaluate the long-term stability of the PE-1-PD-L1 complex and analyzed the key dynamic parameters of the resulting trajectories. As shown in **Figure 8C**, the RMSD of the PD-L1 dimer (blue curve) remained stable within the range of 0.3-0.6 nm throughout the simulation. The RMSD of PE-1 (red curve) stabilized between 0.2 and 0.4 nm after the initial equilibration. This steady RMSD indicates the complex maintains structural integrity, showing that PE-1 binding does not cause significant conformational changes at the PD-L1 dimer interface. The interaction energy breakdown (**Figure 8D**) also showed that both van der Waals (LJ-SR) and electrostatic (Coul-SR) interactions play substantial roles in ligand binding. The van der Waals energy consistently stayed negative, fluctuating between -200 and -300 kcal/mol, while Coulombic energy ranged from -50 to -150 kcal/mol. The strongly negative total interaction energy indicates synergistic stability between these two forces, consistent with the specific hydrogen-bonding and hydrophobic contacts identified in the docking analysis.

We next examined hydrogen bond dynamics to characterize the persistence of specific interactions. As illustrated in **Figure 8E**, the quantity of hydrogen bonds between PE-1 and PD-L1 remained consistently within the range of 1 to 4 during the simulation, with an average of approximately 3. This observation sustained a specific binding model rather than transient or non-specific associations. To assess how PE-1 binding influences the overall structure of PD-L1, we analyzed the Rg and SASA. **Figure 8F** illustrates that in the PE-1-bound state, the PD-L1 dimer’s Rg value slightly fluctuated between approximately 1.95 and 2.15 nm, indicating it maintained a compact structure without unfolding or collapsing. Likewise, the SASA values (**Figure 8G**) stayed steady around 135–155 nm^2^, implying limited exposure of hydrophobic areas and minimal solvent interaction, which supports stable ligand binding.

In line with these findings, RMSF analysis indicated that the interface residues of the PD-L1 dimer exhibit low flexibility, especially within the binding pocket (e.g., residues ~40–60), with RMSF values ranging from ~0.0 to 0.5 nm (**Figure 8H**). This reduced local mobility underscores the rigidity of the binding interface, a crucial factor in strong inhibitory potency. Furthermore, we used the MM/PBSA method on representative MD structures to evaluate PE-1’s binding affinity to PD-L1 by calculating the binding free energy. As illustrated in **Figure 8I**, the calculated binding free energy (ΔG) of the PE-1-PD-L1 complex is -35.072 kcal/mol, indicating spontaneous, high-affinity binding, aligning with the favorable docking scores and stable MD simulations. Residue-wise energy decomposition (**Figure 8J**) pinpointed key residues, particularly TYR56 and TYR123, which contribute significantly to the binding energy. This supports the interaction patterns observed in docking and highlights their crucial role in maintaining the stability of the PE-1-PD-L1 complex. Overall, these findings offer a mechanistic explanation for PE-1’s strong PD-1/PD-L1 inhibitory activity observed in biochemical tests.

### 3.6 Cell-based PD-1/PD-L1 blockade and functional activity assessment of PE-1

**Figure 9:**
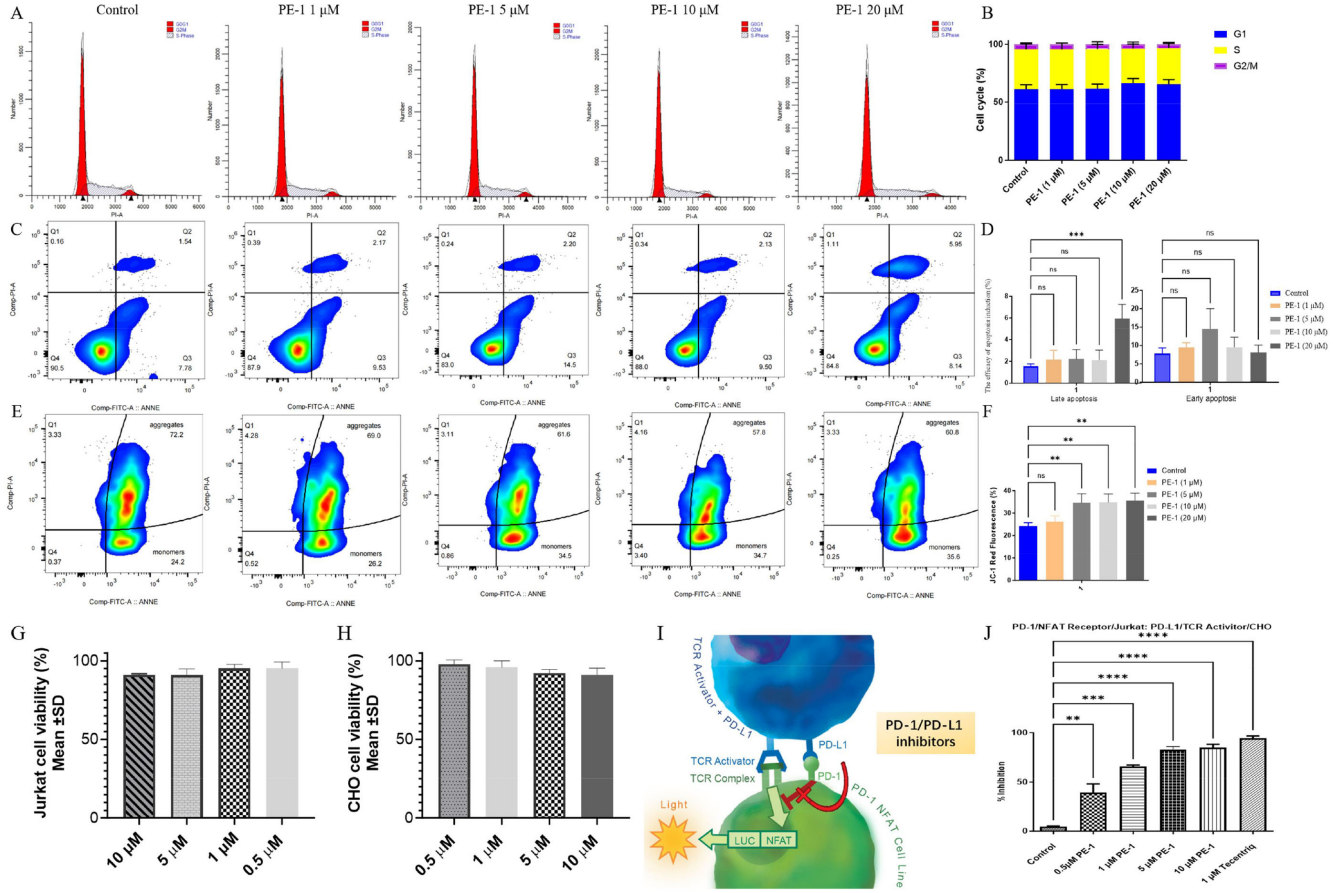
**(A, B)** Effects of PE-1 on cell cycle progression in colon cancer CT-26 cells; **(C, D)** Apoptosis-inducing activity of PE-1 in CT-26 cells; **(E, F)** Impact of PE-1 on mitochondrial membrane potential in CT-26 cells; **(G, H)** Antiproliferative activity of PE-1 against Jurkat T cells and PD-L1-expressing CHO cells, respectively; **(I)** Schematic diagram of the PD-1/PD-L1 NFAT reporter gene assay; **(J)** Functional activity of PE-1 in the PD-L1/PD-1 NFAT reporter model. *P < 0.05, **P < 0.01, ***P <0.001, ****P <0.0001, ns = not significant: P>0.05.

To evaluate the cellular efficacy of PE-1, we first assessed its direct effects on colon cancer cell biology using flow cytometry. Specifically, we analyzed the cell-cycle distribution of murine colorectal carcinoma CT26 cells exposed to PE-1 (1–20 μM) for 24 hours. As demonstrated in **Figures 9A, B**, the PE-1 treatment did not significantly alter the proportions of cells in the G0/G1, S, or G2/M phases compared with the vehicle control, indicating no cell-cycle arrest at pharmacologically relevant concentrations. We next evaluated PE-1-induced apoptosis via Annexin V/PI staining. As depicted in **Figures 9C, D**, only the highest concentration tested (20 μM) elicited a statistically significant increase in late apoptotic cells, whereas lower concentrations showed no discernible pro-apoptotic effect.

To investigate the mechanistic basis of high-dose apoptosis, mitochondrial membrane potential (MMP) was measured utilizing JC-1 dye. As demonstrated in **Figures 9E, F**, PE-1 decreased the red/green fluorescence intensity ratio in a manner proportional to concentration, indicating progressive dissipation of MMP. This finding substantiates mitochondrial dysfunction as a pivotal factor in late-stage apoptosis at high concentrations.

We then evaluated the immunomodulatory activity of PE-1 in Jurkat T cells co-cultured with CHO-K1 cells stably expressing human PD-L1 using a validated NFAT-luciferase reporter assay, which is a physiologically relevant cell-based model of PD-L1/PD-1 interaction. To exclude confounding cytotoxicity, we concurrently quantified the viability of both Jurkat and PD-L1-expressing CHO-K1 cells after 48 h of PE-1 treatment (0.5–10 μM). As shown in **Figures 9G, H**, PE-1 exhibited minimal cytotoxicity, confirming that observed functional effects in the reporter assay reflect specific PD-1/PD-L1 blockade rather than nonspecific cell death. As illustrated in **Figures 9I, J**, PE-1 dose-dependently increased luciferase activity (EC_50_ = 0.61 μM), demonstrating functional antagonism of the PD-L1/PD-1 axis and consequent reactivation of T-cell NFAT signaling. Collectively, these data establish PE-1 as a potent and functionally active small-molecule PD-L1/PD-1 inhibitor in cellular systems, supporting its further preclinical development as a novel checkpoint modulator.

### 3.7 Cell-based assays confirm EZH2 inhibitory activity of PE-1

**Figure 10:**
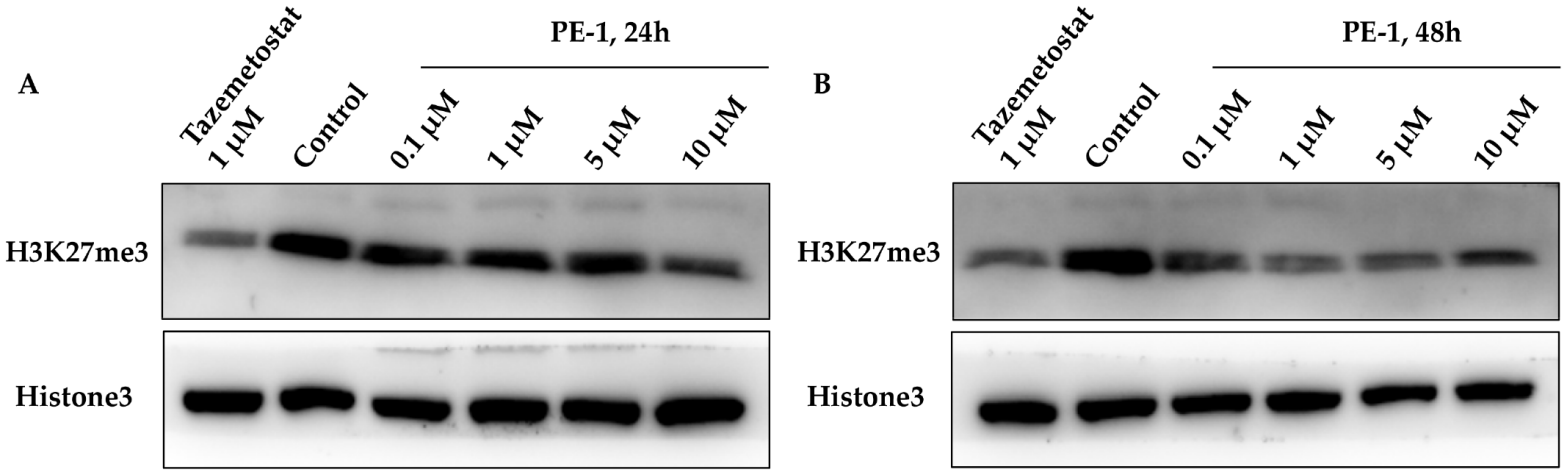
Western blot analysis of H3K27me3 levels in CT26 cells. Cells were treated with different concentrations of PE-1 or 1 μM tazemetostat for 24 h **(A)** and 48 h **(B)**. Total histone H3 was used as the loading control.

To validate intracellular target engagement of PE-1 with EZH2, we performed Western blotting to quantify cellular H3K27me3. This marker serves as the canonical functional readout of endogenous EZH2 methyltransferase activity, and total histone H3 was used as the loading control. CT26 cells were treated with serial dilutions of PE-1 at concentrations of 0.1, 1, 5, and 10 μM for 24 or 48 h. As a positive control, 1 μM tazemetostat, a clinically validated and selective EZH2 inhibitor, was tested at the same concentrations and incubation time points for direct comparison. Densitometric analysis normalized to total histone H3 revealed that all tested concentrations of PE-1 significantly reduced H3K27me3 levels relative to the untreated control group. At 24 h, H3K27me3 levels in cells treated with 0.1, 1, 5, and 10 μM PE-1 were 81.9%, 65.1%, 64.2%, and 50.3% of the control group, respectively. Under identical experimental conditions, 1 μM tazemetostat lowered H3K27me3 to 23.9% of the control level (**Figure 10A**). At 48 h, the corresponding values for the four PE-1 treatment groups were 70.1%, 41.6%, 63.1%, and 54.6%, while 1 μM tazemetostat reduced H3K27me3 to 31.2% of the control (**Figure 10B**). Notably, PE-1 at 1 μM exerted a suppressive effect on H3K27me3 comparable to that of tazemetostat at both time points. Although the dose-response relationship was not strictly linear across the tested concentration range, PE-1 consistently and significantly inhibited EZH2-mediated H3K27 trimethylation at all doses. Collectively, these cellular data confirm that PE-1 can effectively bind and inhibit EZH2 in CT26 cells. The results are consistent with findings from previous biochemical assays on purified EZH2 enzyme activity and direct target binding.

### 3.8 *In vivo* pharmacokinetic profile of PE-1

We systematically evaluated the pharmacokinetic (PK) profile of PE-1 in male Sprague-Dawley (SD) rats following single-dose administration via three routes: intravenous (I.V., 2 mg/kg), intraperitoneal (I.P., 4 mg/kg), and oral (P.O., 50 mg/kg) (**Table 2**). Blood samples were collected serially up to 48 h post-dose, and plasma concentrations were quantified using a validated LC-MS/MS method. Following I.V. administration, PE-1 exhibited moderate systemic clearance (CL = 0.97 L/h/kg) and a volume of distribution at steady state (Vz = 9.3 L/kg), indicating extensive tissue distribution beyond plasma. The terminal half-life (*t*_1/2_) was 6.7 h, with an AUC_(0-∞)_ of 2064.4 ng·h/mL and *C*_max_ of 1872.5 ng/mL, consistent with sustained systemic exposure and favorable drug retention. After IP dosing, PE-1 was rapidly and completely absorbed (*T*_max_ = 0.25 h), achieving an AUC_(0-∞)_ of 2231.2 ng·h/mL and *C*_max_ of 685.5 ng/mL. Oral administration yielded robust exposure: AUC_(0-∞)_ = 30173.1 ng·h/mL, *C*_max_ = 2488.4 ng/mL, and *T*_max_ = 1.1 h. Critically, the oral bioavailability relative to I.V. administration was 58.4%, markedly exceeding the typical threshold (>20-30%) for the oral bioavailability of small molecules. This high *F*% reflects efficient gastrointestinal absorption and low presystemic extraction, as evidenced by the fact that the mean residence time (MRT_(0−∞)_) after PO dosing was 9.4 h, and the terminal *t*_1/2_ was 6.1 h, indicating prolonged systemic presence without accumulation. Clearance (CL = 2.6 L/h/kg) and apparent volume of distribution (Vz = 22.6 L/kg) further confirm favorable disposition properties. Collectively, these data demonstrate that PE-1 possesses a potent and drug-like PK profile: low-to-moderate clearance, extensive distribution, predictable multi-route absorption, and most notably, high and reproducible oral bioavailability. Its pharmacokinetic properties meet key criteria for clinical advancement, positioning PE-1 as a promising candidate for oral immunotherapy development.

**Table 2:** Primary pharmacokinetic properties of PE-1 in SD rats (n = 3, mean ± SD).

Pharmacokinetic parameters	I.V. (2 mg/kg)	I.P. (4 mg/kg)	P.O. (50 mg/kg)
AUC_(0–t)_ (ng/mL·h)	2054.7 ± 113.2	2208.2 ± 136.9	29081.4 ± 3481.3
AUC_(0–∞)_ (ng/mL·h)	2064.4 ± 123.1	2231.2 ± 145.1	30173.1 ± 3590.5
MRT_(0–t)_ (h)	8.2 ± 1.0	5.7 ± 0.5	9.2 ± 1.3
MRT_(0–∞)_ (h)	8.5 ± 1.1	6.0 ± 0.6	9.4 ± 1.2
*t*_1/2_ (h)	6.7 ± 0.6	5.9 ± 0.5	6.1 ± 0.7
*T*_max_ (h)	0.083	0.25	1.1 ± 0.5
CL (L/h/kg)	0.9 ± 0.1	1.7 ± 0.7	2.6 ± 1.0
Vz (L/kg)	9.3 ± 0.4	9.4 ± 0.8	22.6 ± 5.1
*C*_max_ (ng/mL)	1872.5 ± 102.3	685.5 ± 90.1	2488.4 ± 156.1
*F* (%)			58.4

### 3.9 *In vivo* antitumor efficacy of PE-1 in a CT26 colon cancer model

**Figure 11:**
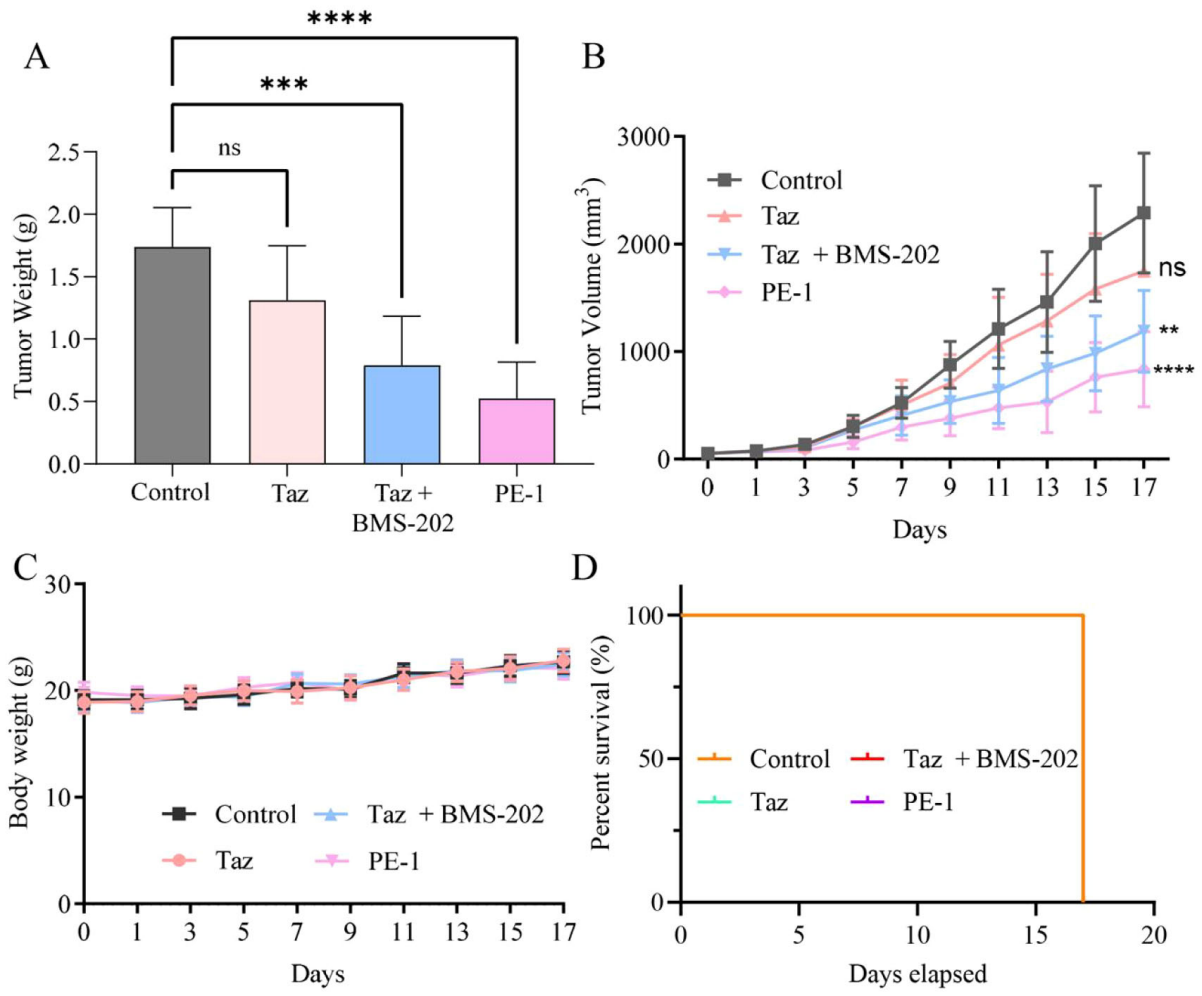
*In vivo* tumor-suppressing effects of PE-1. Mice were treated with the control group, PE-1 (50 mg/kg, p.o.× QD), Taz (50 mg/kg, p.o.× QD), and Taz (50 mg/kg, p.o.× QD) + BMS-202 (50 mg/kg, p.o.× QD) for 17 consecutive days. **(A)** weights of tumors; **(B)** changes in tumor volume; **(C)** body weight change of mice; **(D)** percent survival of mice. *P < 0.05, **P < 0.01, ***P <0.001, ****P <0.0001, ns = not significant: P>0.05.

Given the concurrent overexpression of EZH2 and PD-L1 in human colon adenocarcinoma and their established roles in promoting tumor cell proliferation and immune evasion, we evaluated the therapeutic potential of PE-1, a rationally designed dual-target inhibitor, in the immunocompetent CT26 murine colon cancer model. As shown in **Figures 11A**, PE-1, administered orally at 50 mg/kg (QD, days 1-17), induced a statistically significant reduction in final tumor weight compared with the vehicle control, resulting in 69.9% tumor growth inhibition (TGI). This activity significantly surpassed that of the selective EZH2 inhibitor tazemetostat (50 mg/kg, once daily; TGI = 24.6%) and the combination of tazemetostat (50 mg/kg) with the small-molecule PD-L1 inhibitor BMS-202 (50 mg/kg, once daily; TGI = 54.4%). Consistent with these endpoint measurements, longitudinal tumor volume monitoring (**Figure 11B**) revealed that PE-1 treatment durably suppressed tumor progression throughout the 17-day study period. By day 17, PE-1 achieved a TGI of 62.1% relative to vehicle, significantly outperforming both tazemetostat monotherapy (TGI = 22.6%) and the tazemetostat/BMS-202 combination (TGI = 46.1%). Notably, the PE-1 group exhibited a flattened tumor growth curve after day 7, suggesting sustained pharmacodynamic engagement. Importantly, no treatment-related body weight loss exceeding 10% was observed in any group (**Figure 11C**). Survival analysis (**Figure 11D**) further corroborated the superior efficacy: all mice treated with PE-1 remained alive through the full 17-day observation period. This confirms the favorable tolerability of PE-1 monotherapy at the efficacious dose, with a wide therapeutic index. Collectively, these data demonstrate that PE-1 exhibits superior efficacy, durable tumor control, a survival benefit, and a favorable tolerability profile, positioning it as a compelling preclinical candidate for the treatment of colon cancer in a setting with high unmet need in immuno-oncology.

**Figure 12:**
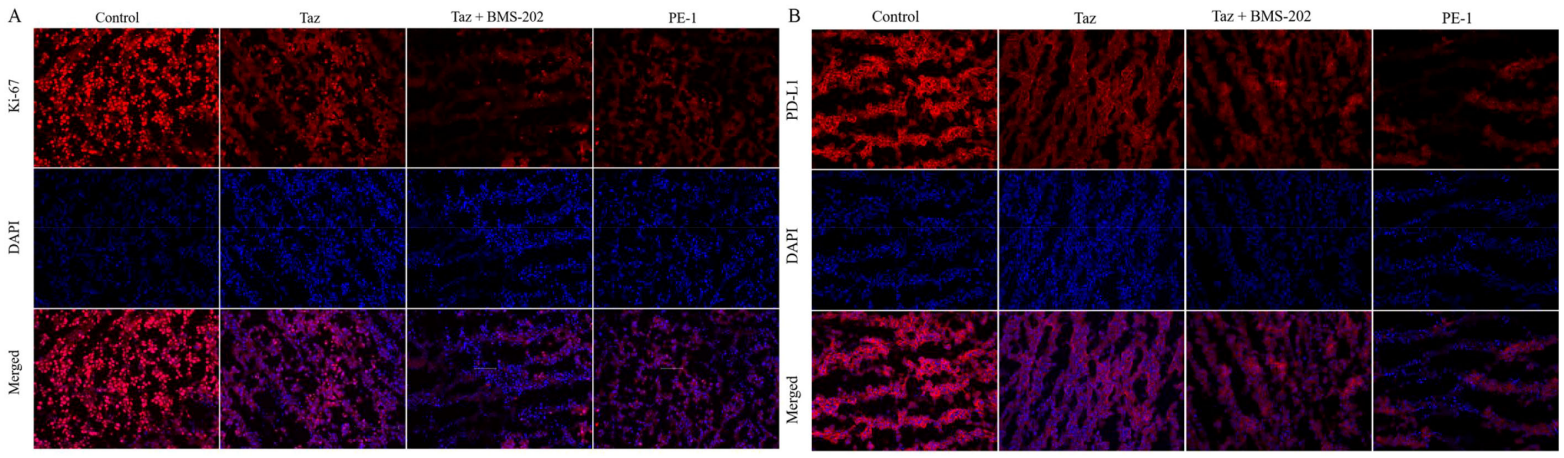
Immunofluorescence detection of Ki-67 **(A)** and PD-L1 **(B)** expression in CT26 tumor tissues from different treatment groups.

To better understand how PE-1 fights tumors, we performed biomarker staining on CT26 tumor tissues to characterize the expression profiles of key functional markers. The selected biomarkers encompass fundamental biological processes: Ki-67 (a standard marker of cell proliferation) and PD-L1 (a significant biomarker for predicting the therapeutic efficacy of immune checkpoint inhibitors). As depicted in **Figure 12A**, Ki-67 expression was significantly downregulated in PE-1-treated tumors compared with the solvent control and was superior to that of the reference compound tazemetostat. Notably, PD-L1 fluorescent signals were substantially reduced in PE-1 treatment groups, providing direct evidence that PE-1 effectively enhances tumor immune function (**Figure 12B**). Collectively, these results reveal that PE-1 suppresses tumor cell proliferation and downregulates PD-L1 expression in CT26 tumor tissues, thereby jointly restraining tumor growth and improving the tumor immune microenvironment, further highlighting the promising application prospects of PE-1 for tumor treatment.

**Figure 13:**
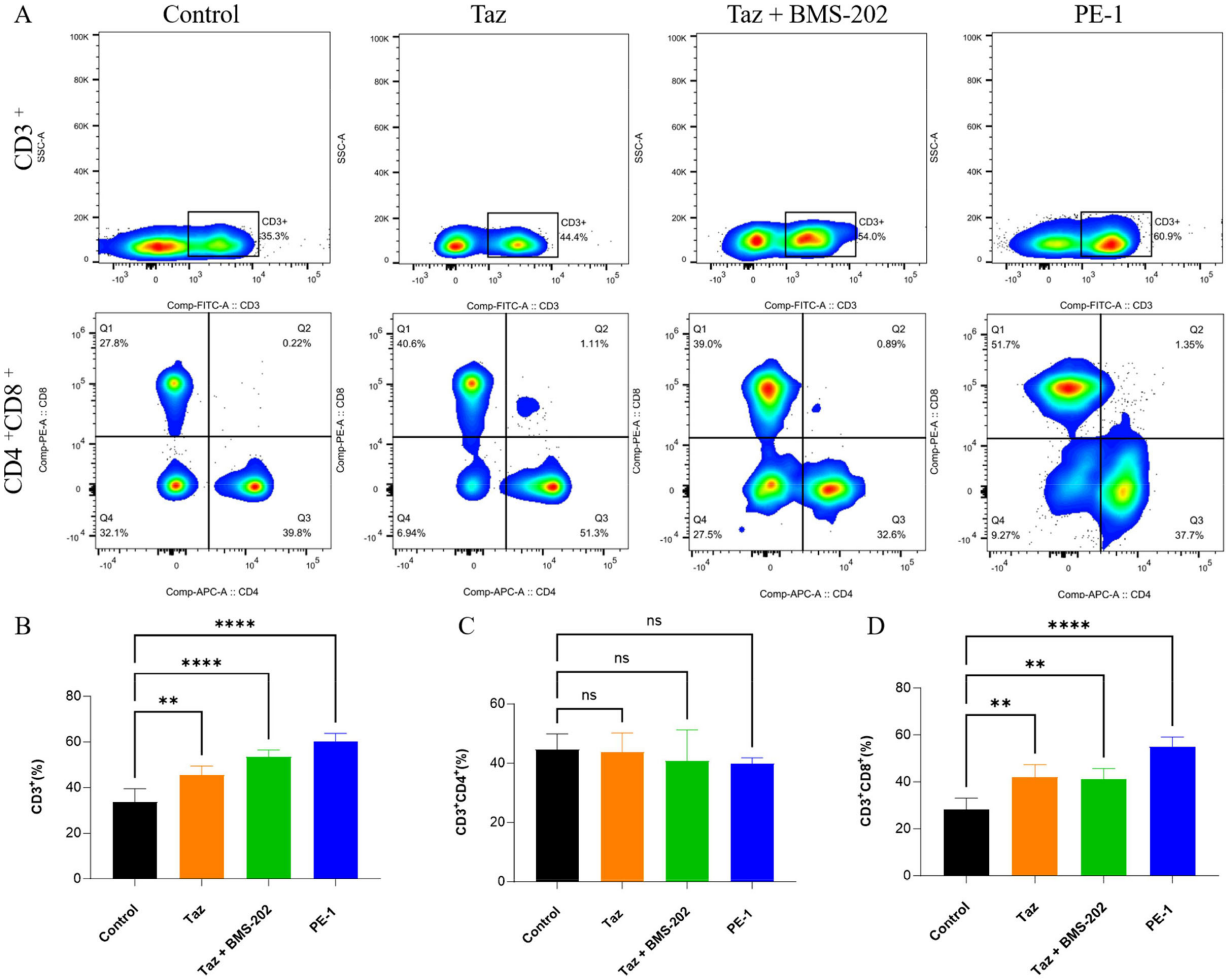
Flow cytometric analysis of intratumoral T lymphocyte subsets. **(A)** Representative flow cytometry dot plots showing the frequencies of CD3^+^ T cells and CD4^+^CD8^+^ double-positive T cells in tumor-infiltrating leukocytes from CT-26 tumor-bearing mice. Quantitative analysis of **(B)** CD3^+^, **(C)** CD4^+^, and **(D)** CD8^+^ T cell populations. Data are presented as mean ± SD, *P < 0.05, **P < 0.01, ***P < 0.001, ****P < 0.0001, one-way analysis of variance (ANOVA). ns = not significant: P>0.05.

To elucidate the immunomodulatory mechanism underlying the antitumor activity of PE-1, we performed flow cytometry to assess tumor-infiltrating lymphocyte (TIL) infiltration in the CT26 colon tumor microenvironment and cytokine profiling of tumor tissue homogenates. As shown in **Figures 13A, B**, flow cytometry analysis revealed that PE-1 treatment significantly increased the proportion of CD3^+^ T cells in tumor tissues. PE-1 monotherapy increased CD3^+^ T cell infiltration from 33.7% in the control group to 60.3%, which was better than Taz (45.4%) and the combination of Taz with BMS-202 group (53.4%). Among CD3^+^ T cells, the frequency of CD8^+^ cytotoxic T lymphocytes (CTLs) showed a more pronounced increase (**Figure 13D**). PE-1 monotherapy increased CD8^+^ T cell infiltration from 28.1% in controls to 55.0%, while the combination therapy increased it to 41.0%, indicating activation of effector T cell responses. In contrast, the proportion of CD4^+^ T cells remained unchanged across all treatment groups (**Figure 13C**), confirming the selective enrichment of cytotoxic CD8^+^ T cells. Collectively, these findings demonstrate that PE-1 remodels the tumor immune microenvironment by promoting the infiltration and activation of CD8^+^ cytotoxic T lymphocytes, supporting its translational potential as an immunotherapeutic agent for colorectal cancer.

**Figure 14:**
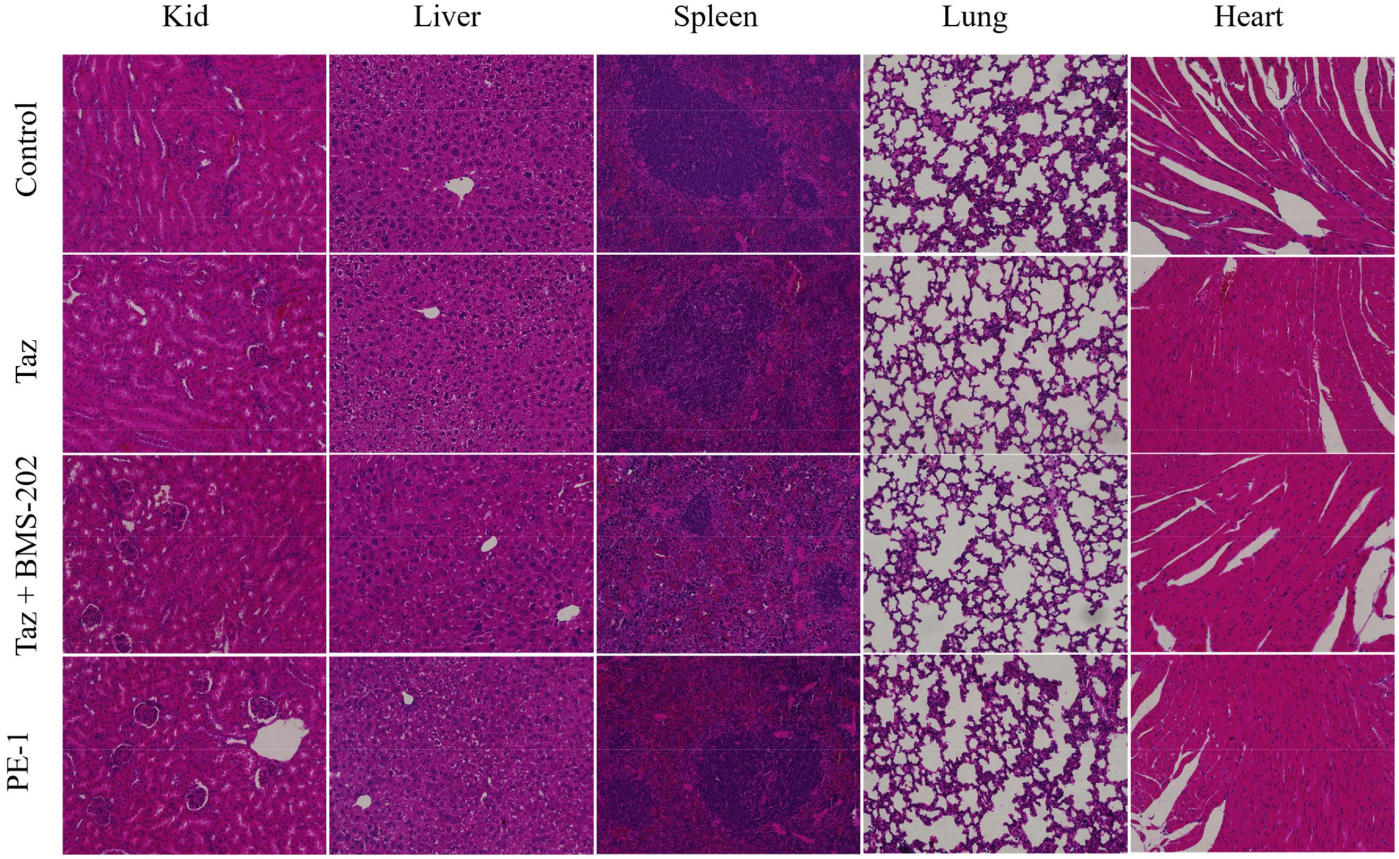
*In vivo* safety assessment of PE-1 in CT-26 tumor-bearing mice. H&E staining of heart, liver, and kidney tissue sections; no histopathological abnormalities were observed compared with vehicle-treated controls.

For clinical translation, a thorough *in vivo* safety assessment is essential. We examined PE-1’s safety profile using histopathological analyses, focusing on the liver, kidney, and heart—key organs vulnerable to off-target toxicity in clinical oncology. Organs from treated mice were collected and stained with hematoxylin and eosin (H&E) for histopathology. As shown in **Figure 14**, there was no evident tissue damage, structural abnormalities, or lesions in these organs from mice treated with PE-1, consistent with the vehicle control group. These findings confirm that PE-1 is well tolerated. By avoiding organ toxicities that often limit clinical progress, PE-1 overcomes a major barrier and shows promise as a safe candidate for moving from preclinical to clinical trials.

## 4 Discussion

First-generation immune checkpoint inhibitors, particularly monoclonal antibodies targeting the PD-1/PD-L1 axis, have emerged as foundational therapeutics in immuno-oncology. However, their clinical utility remains limited by modest overall response rates, typically below 20%, prompting substantial efforts to develop synergistic combination strategies. While co-administration of PD-L1 inhibitors with other antitumor agents has been extensively explored, such multi-drug regimens often lead to cumulative toxicity and poorly characterized pharmacokinetic/pharmacodynamic (PK/PD) interactions, thereby limiting their translational potential. Consequently, dual-target small molecules, single chemical entities that simultaneously modulate two disease-relevant pathways, have emerged as an innovative alternative. Compared with combination therapies, dual-target inhibitors offer inherent advantages: integrated pharmacology, predictable PK/PD profiles, improved therapeutic indices, and streamlined structure-activity relationship (SAR) optimization enabled by unified molecular frameworks. It is noteworthy that current small-molecule PD-L1 inhibitors are hindered by excessive lipophilicity, leading to suboptimal oral bioavailability and unfavorable pharmacokinetic profiles, which are significant obstacles to their clinical development and translation. Therefore, the rational design of orally bioavailable, dual-target inhibitors that engage PD-L1 and a functionally complementary target constitutes a strategic priority for advancing next-generation immuno-oncology therapeutics.

## 5 Conclusions

Herein, we report the rational design, synthesis, and characterization of a novel series of bifunctional EZH2/PD-L1 inhibitors optimized for oral delivery. Compound PE-1 emerged as the lead candidate, exhibiting nanomolar potency against both targets: IC_50_ = 48.1 nM against PD-L1 (determined by homogeneous time-resolved fluorescence, HTRF) and IC_50_ = 101.2 nM against EZH2 methyltransferase activity (measured via MTase-Glo assay). Binding affinity and stoichiometry were independently confirmed by BLI and MST, validating its dual-target engagement at the biophysical level. Molecular docking simulations have indicated that PE-1 binds with high complementarity to the dimerization interfaces of both PD-L1 and EZH2, structural features critical for functional inhibition, providing a mechanistic rationale for its dual efficacy. In syngeneic mouse models, oral administration of PE-1 elicited robust antitumor activity, achieving tumor growth inhibition (TGI) of 69.9% in CT26 colon carcinoma, without observable systemic toxicity. Collectively, these data establish PE-1 as a pharmacologically optimized dual PD-L1/EZH2 inhibitor with good preclinical efficacy, positioning it as a promising candidate for further clinical evaluation of dual-pathway immunotherapeutic regimens.

## Data Availability

Publicly available datasets were analyzed in this study. All raw and processed data supporting the conclusions of this manuscript are either presented within the article text, figures, and tables, or provided as **Supplementary Materials**. No additional data repositories or accession numbers were created for this study.
