# Supplementary material for: Rational design and biological validation of EZH2/PD-L1 bifunctional inhibitors for colorectal cancer immunotherapy
Source: Front Immunol. 2026 Jul 30;17:1886069. doi: 10.3389/fimmu.2026.1886069 (PMC13469651; doi:10.3389/fimmu.2026.1886069)
Supplement: Supplementary file 1 [file DataSheet1.pdf]

Supporting Information

Rational Design and Biological Validation of EZH2/PD-L1 Bi-functional Inhibitors for Colorectal Cancer Immunotherapy

Table of Contents

Copies of PE1-10 ^{1}H NMR, ^{13}C NMR, MS, and HPLC spectra ……………………………………………………………………S2

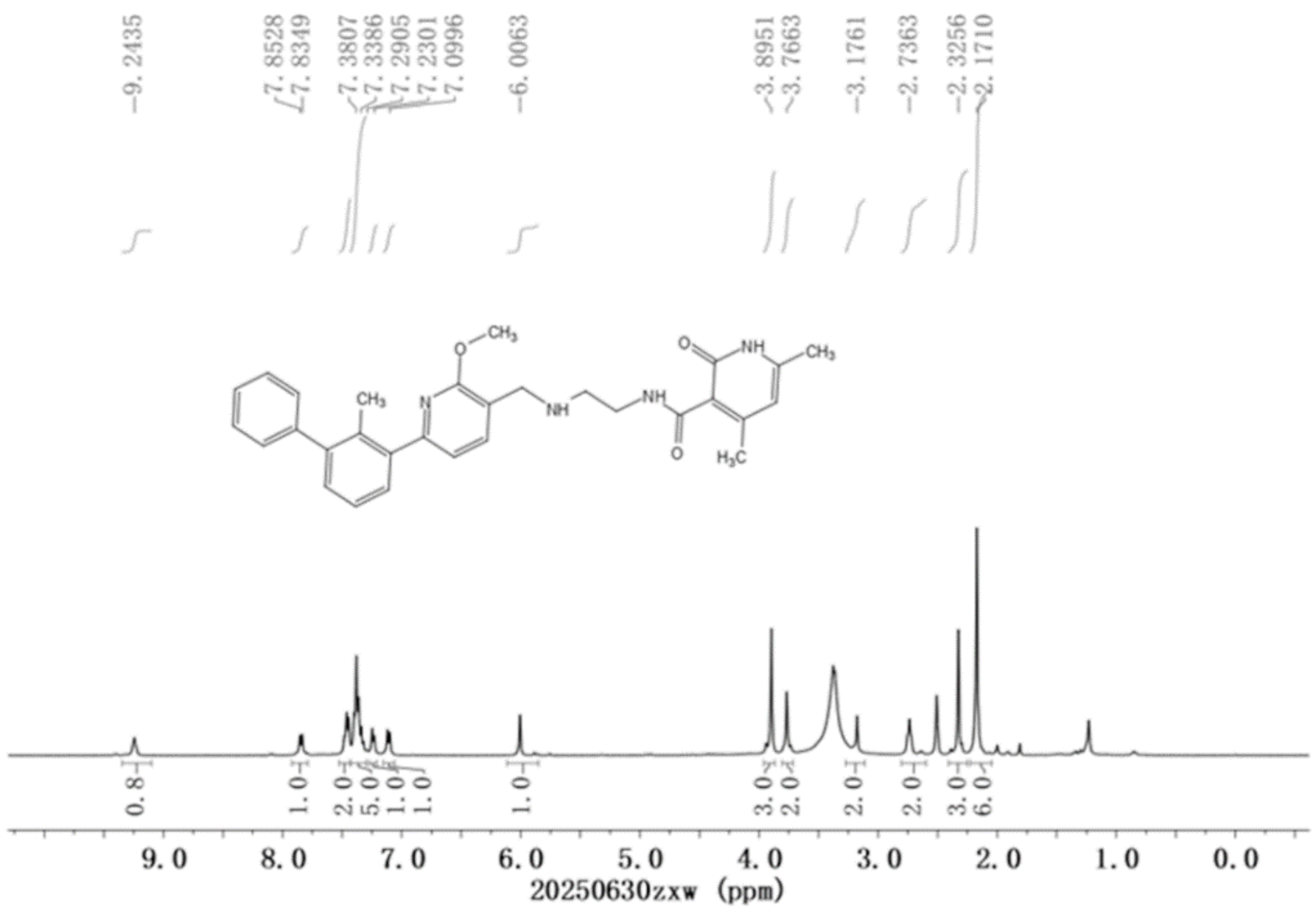
20250630zxw (ppm)

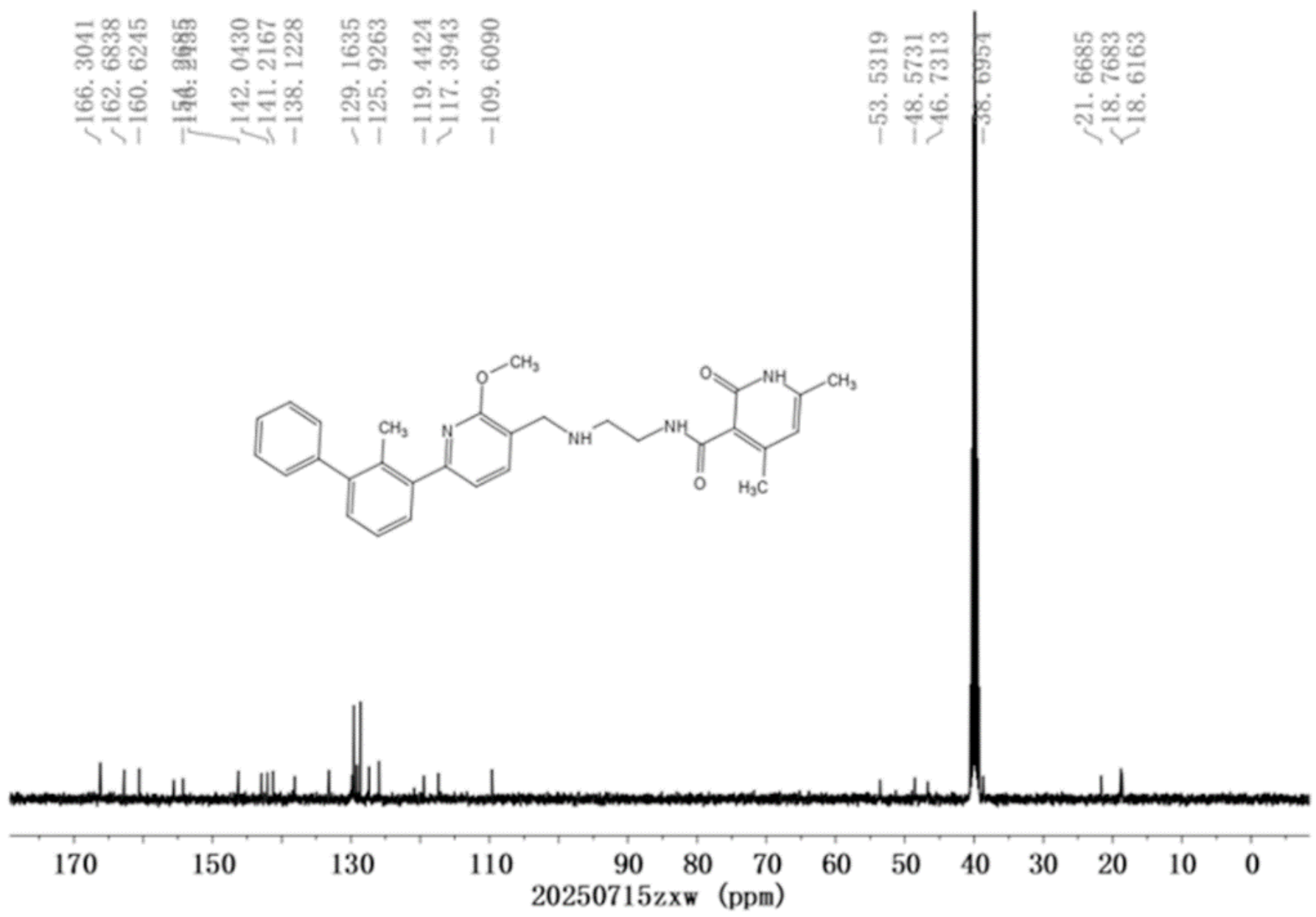
20250715zxw (ppm)

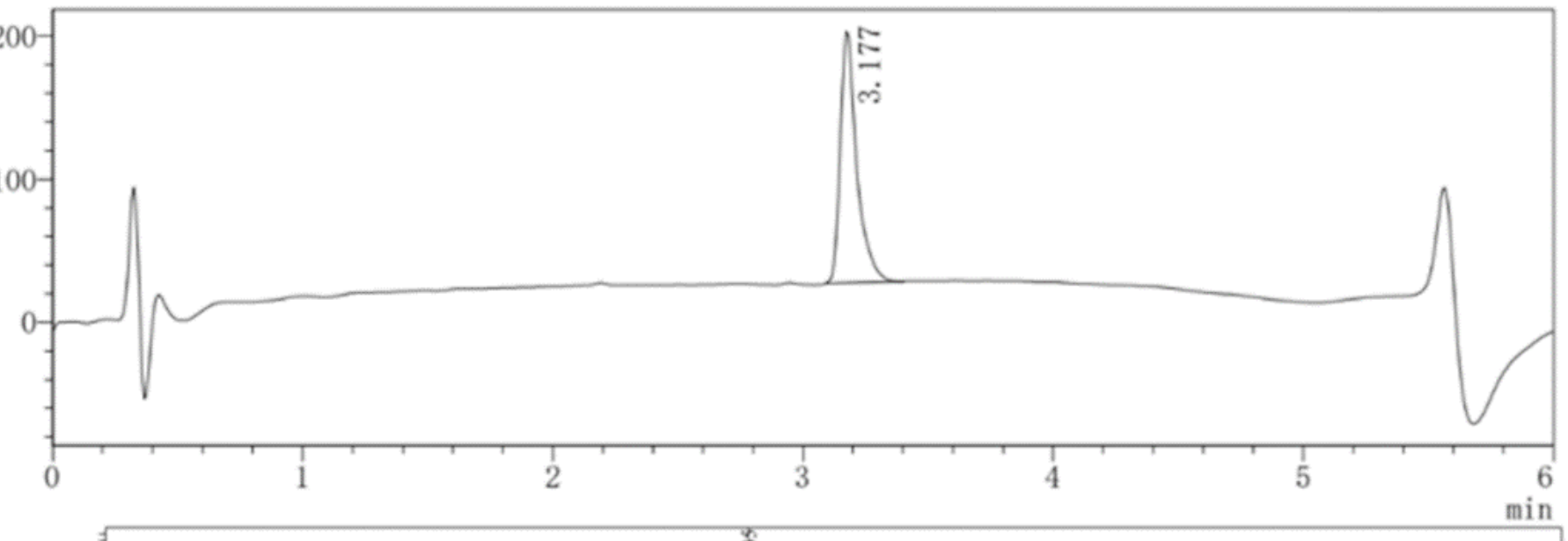
3.177
min

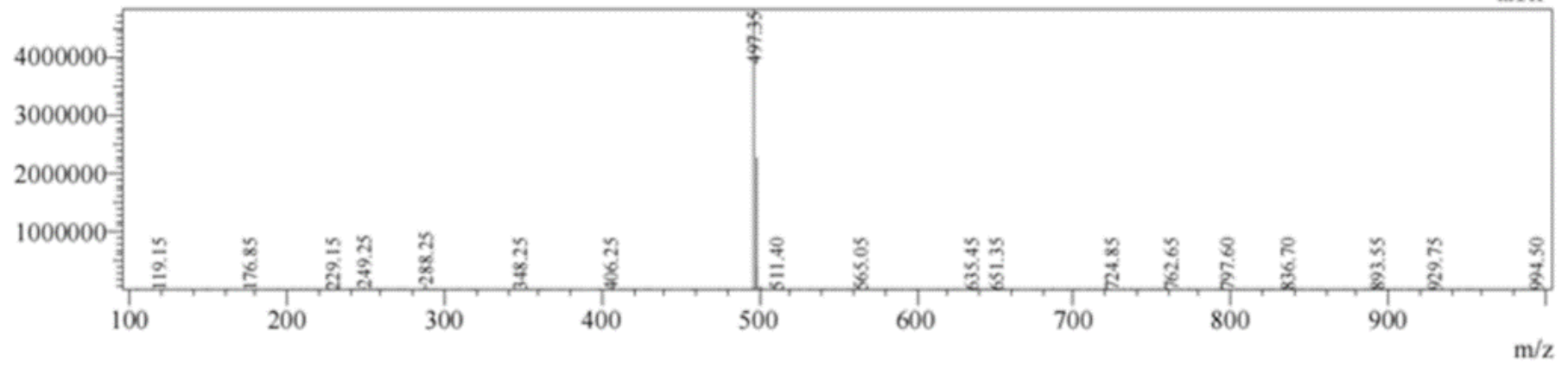
497.35
m/z

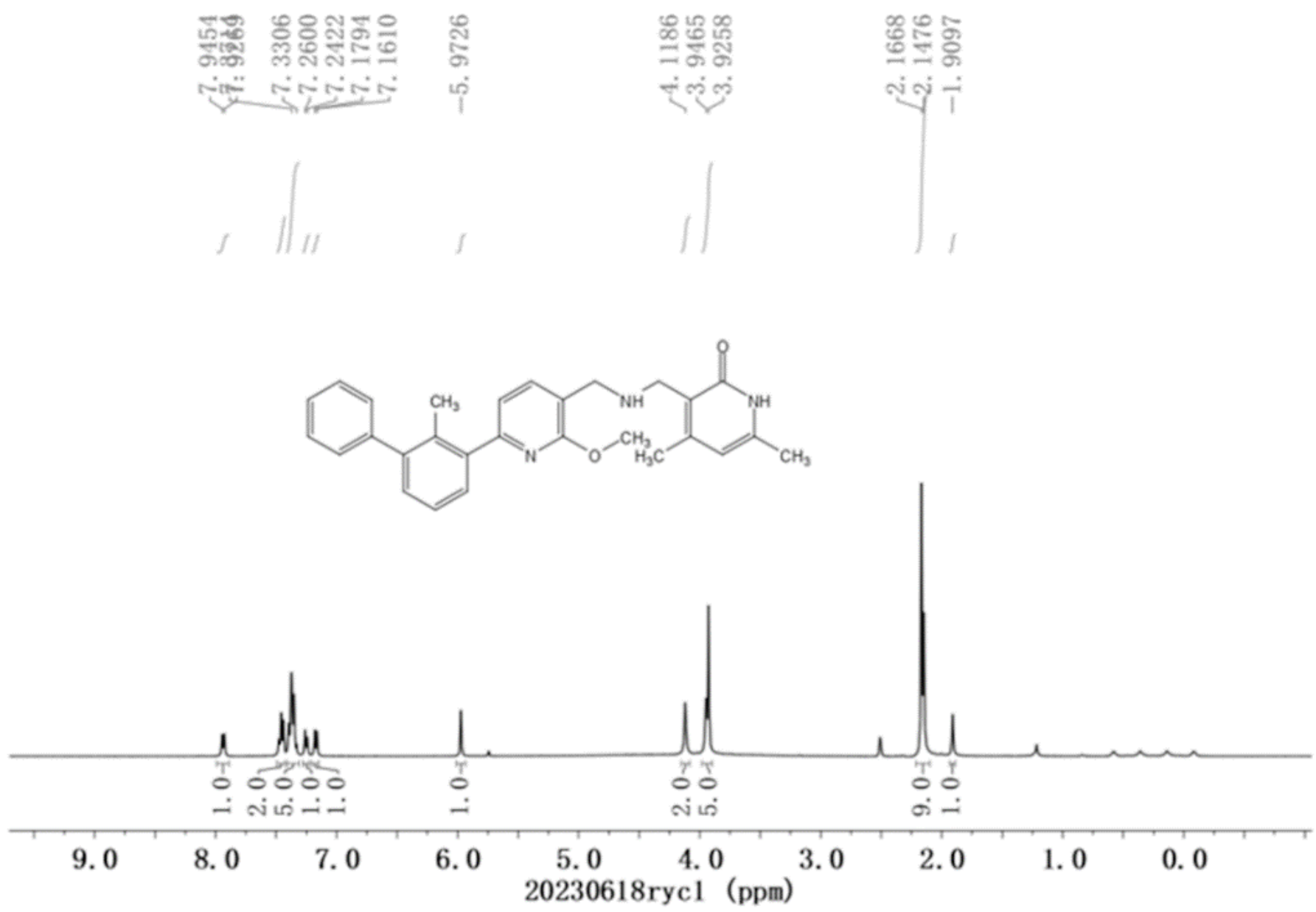

7.9454
7.3306
7.2600
7.2422
7.1794
7.1610
5.9726
4.1186
3.9465
3.9258
2.1668
2.1476
1.9097
CH3
NH
O
N
H3C
1.0
2.0
5.0
1.0
1.0
1.0
2.0
5.0
9.0
1.0
9.0
8.0
7.0
6.0
5.0
4.0
3.0
2.0
1.0
0.0
20230618ryc1 (ppm)

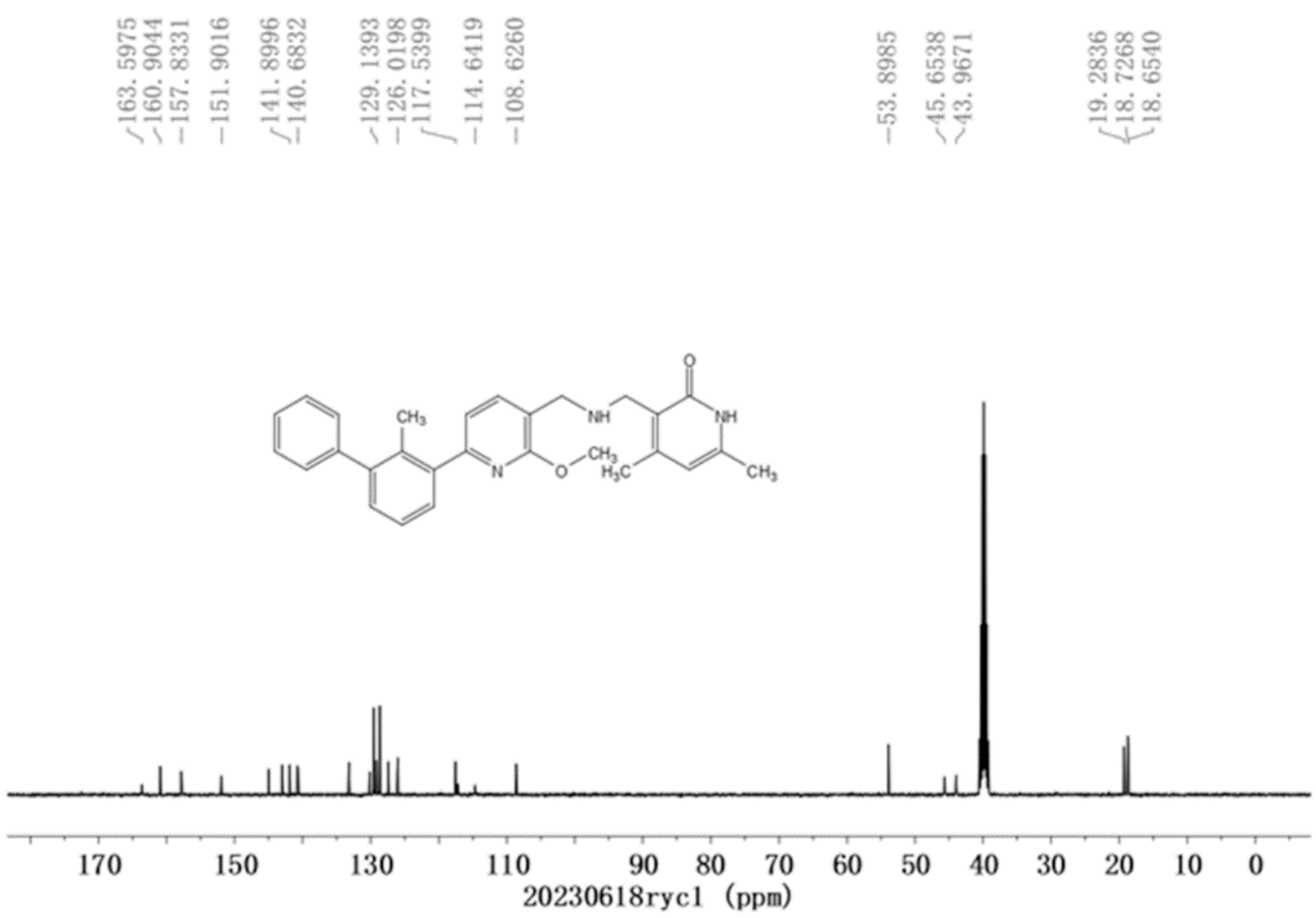

163.5975
160.9044
157.8331
151.9016
141.8996
140.6832
129.1393
126.0198
117.5399
114.6419
108.6260
53.8985
45.6538
43.9671
19.2836
18.7268
18.6540
CH3
NH
O
N
H3C
170
150
130
110
90
80
70
60
50
40
30
20
10
0
20230618ryc1 (ppm)

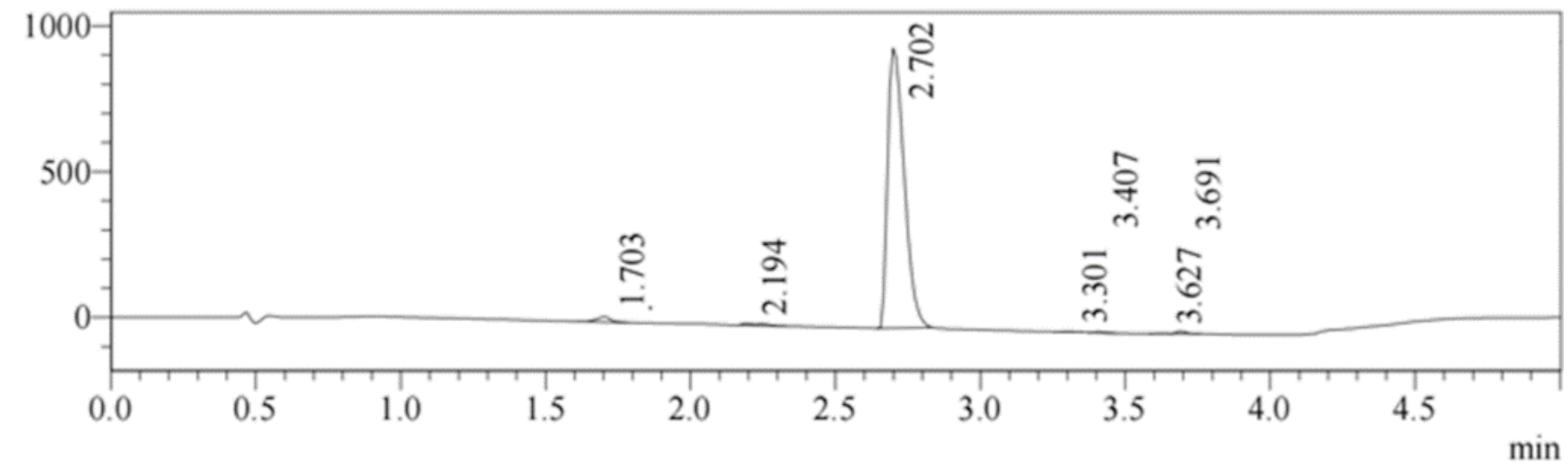

1000
500
0
2.702
1.703
2.194
3.301
3.407
3.627
3.691
0.0
0.5
1.0
1.5
2.0
2.5
3.0
3.5
4.0
4.5
min

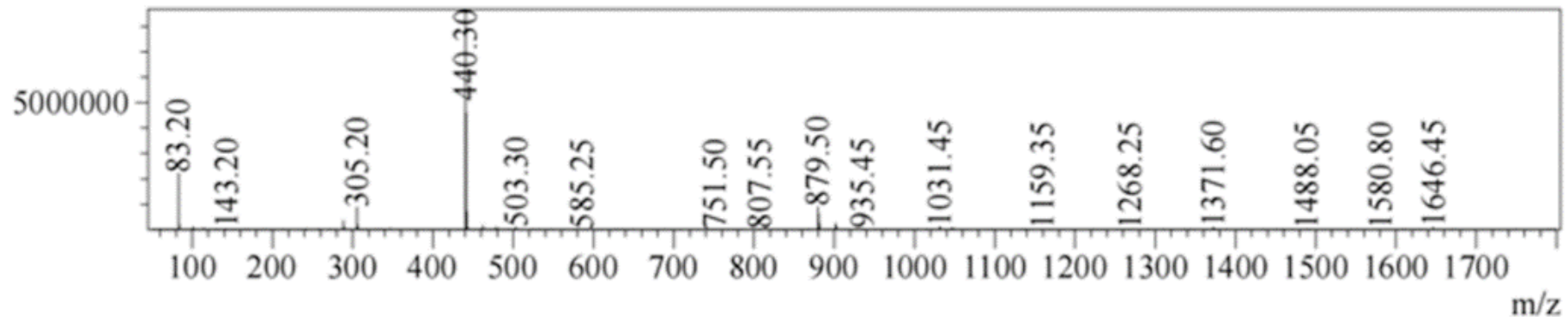

5000000
83.20
143.20
305.20
440.30
503.30
585.25
751.50
807.55
879.50
935.45
1031.45
1159.35
1268.25
1371.60
1488.05
1580.80
1646.45
100
200
300
400
500
600
700
800
900
1000
1100
1200
1300
1400
1500
1600
1700
m/z

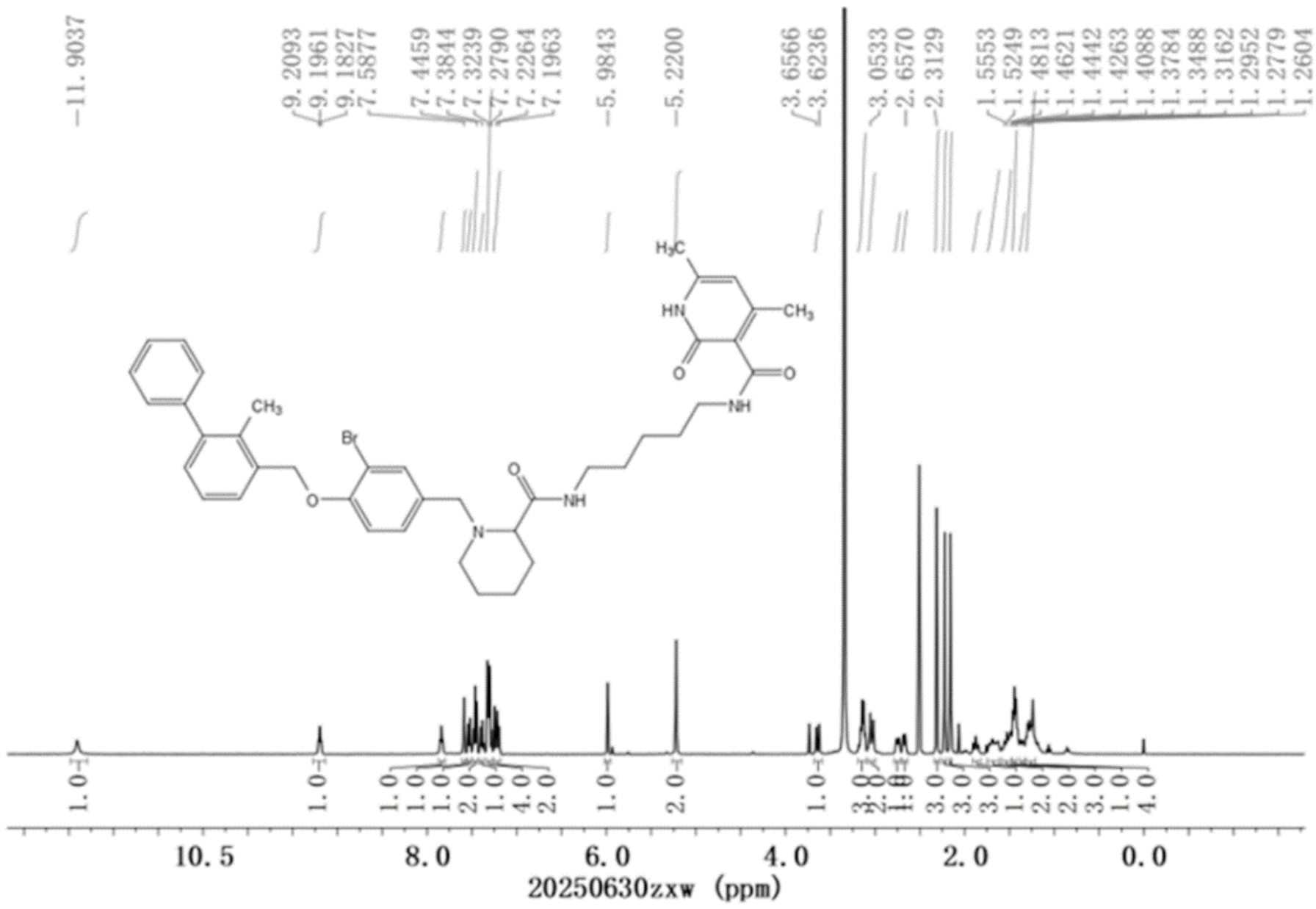
20250630zxw (ppm)

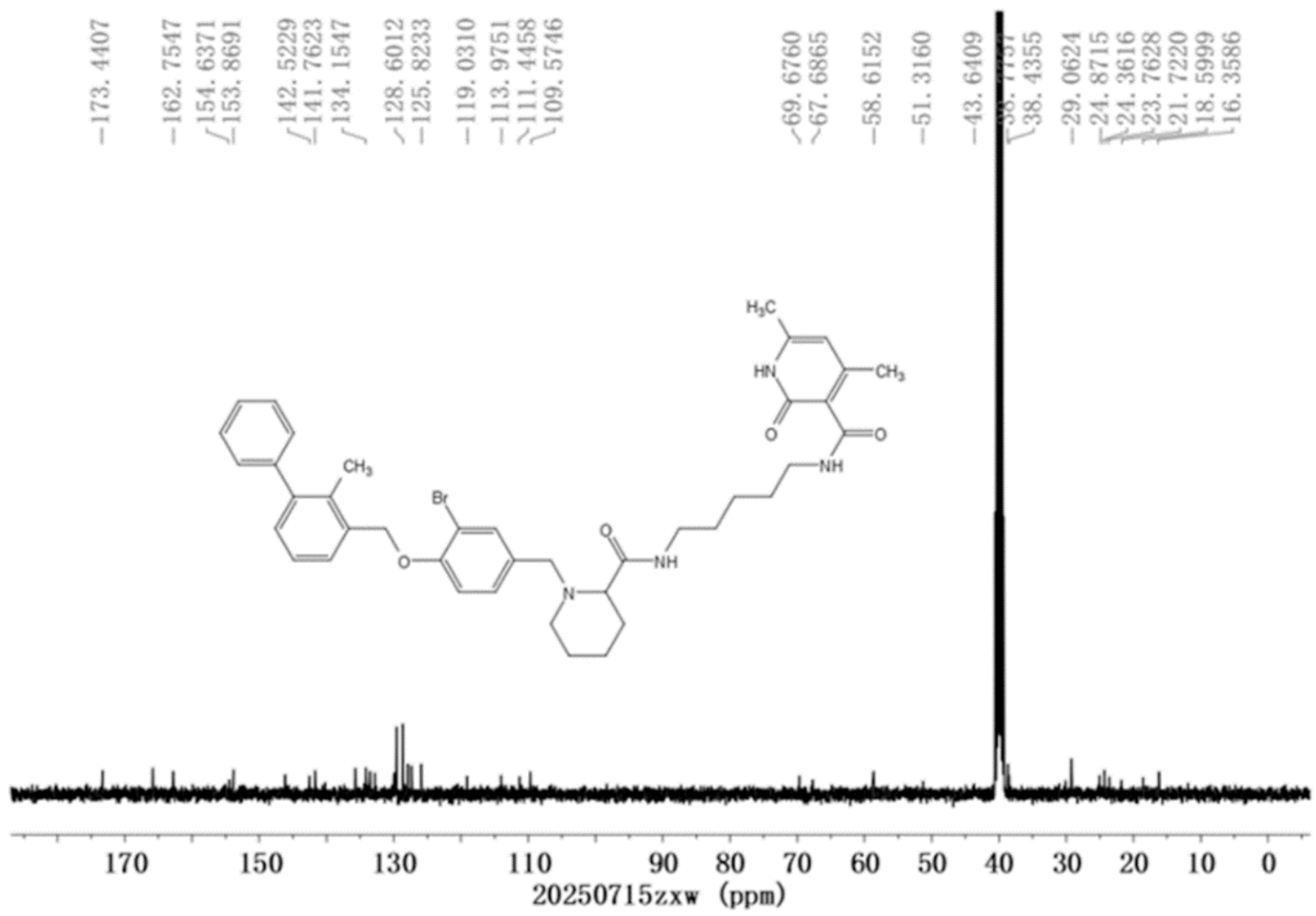
20250715zxw (ppm)

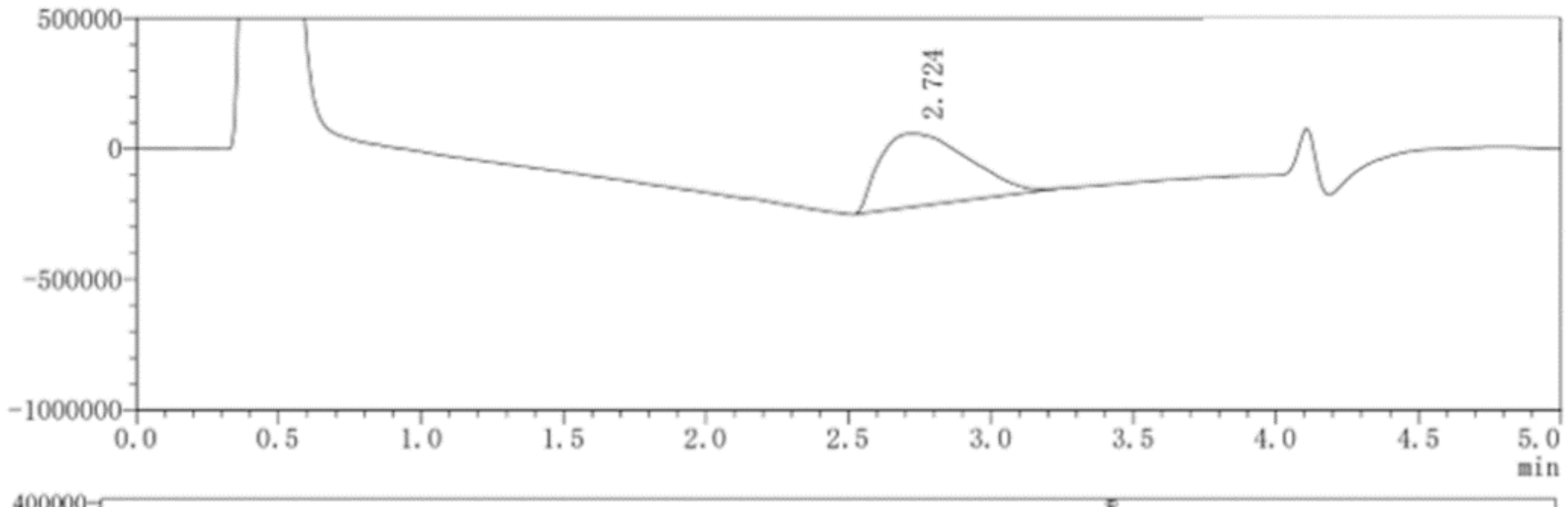

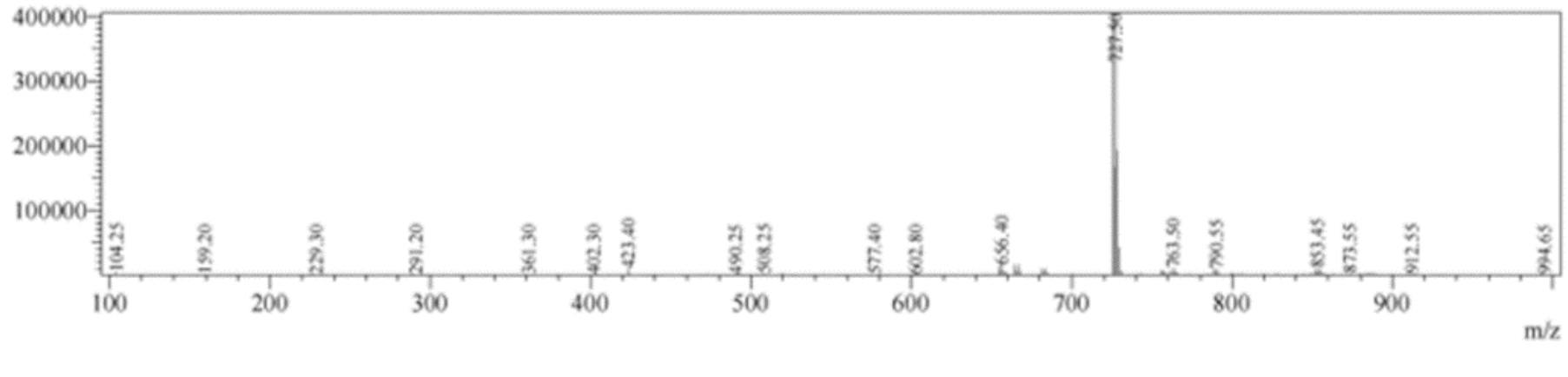

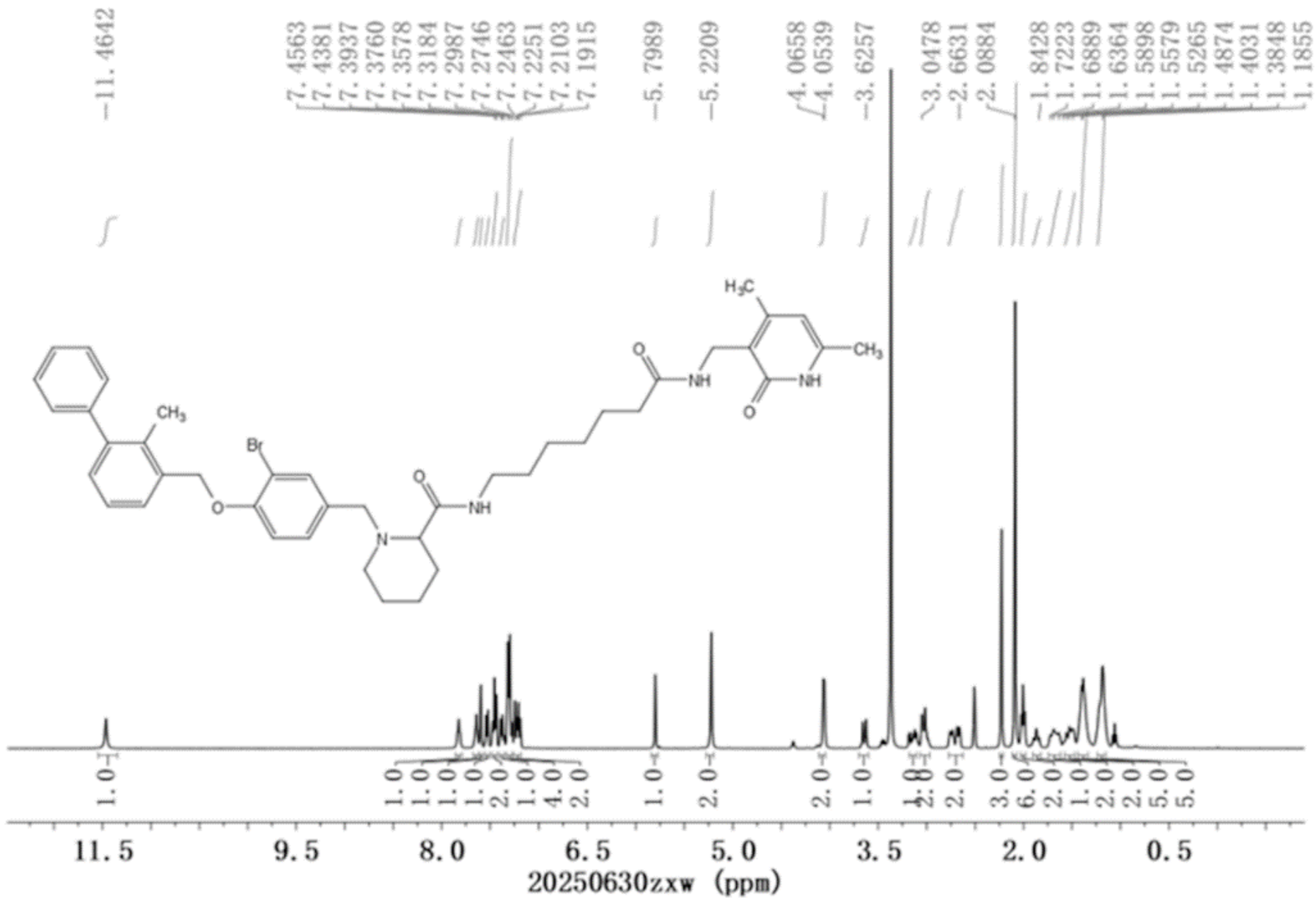
-11.4642
-5.7989
-5.2209
-3.6257
H3C
CH3
NH
O
Br
N
11.5 9.5 8.0 6.5 5.0 3.5 2.0 0.5
20250630zxw (ppm)

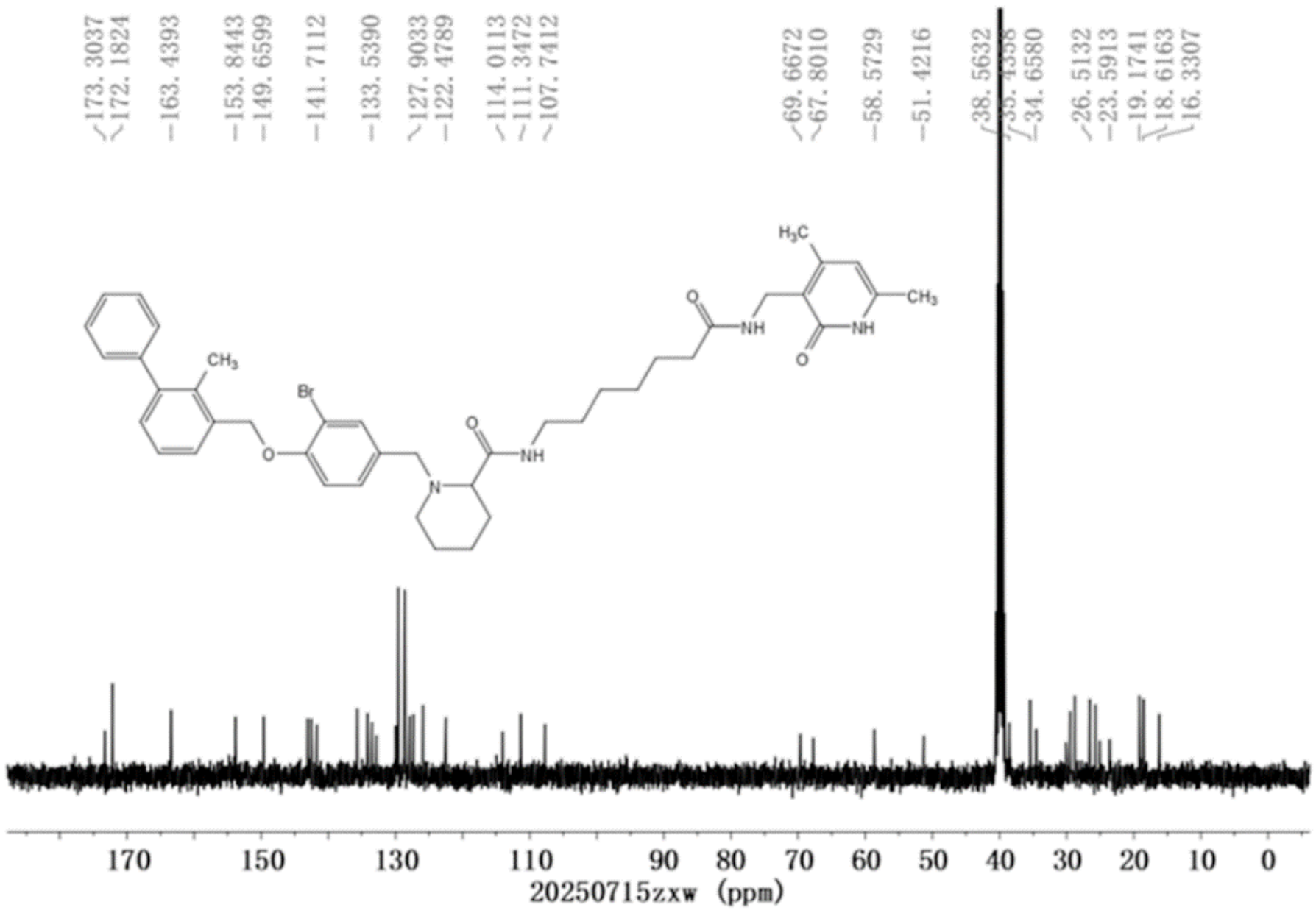
173.3037
172.1824
163.4393
153.8443
149.6599
141.7112
133.5390
127.9033
122.4789
114.0113
111.3472
107.7412
69.6672
67.8010
58.5729
51.4216
38.5632
34.6580
26.5132
23.5913
19.1741
18.6163
16.3307
170 150 130 110 90 80 70 60 50 40 30 20 10 0
20250715zxw (ppm)

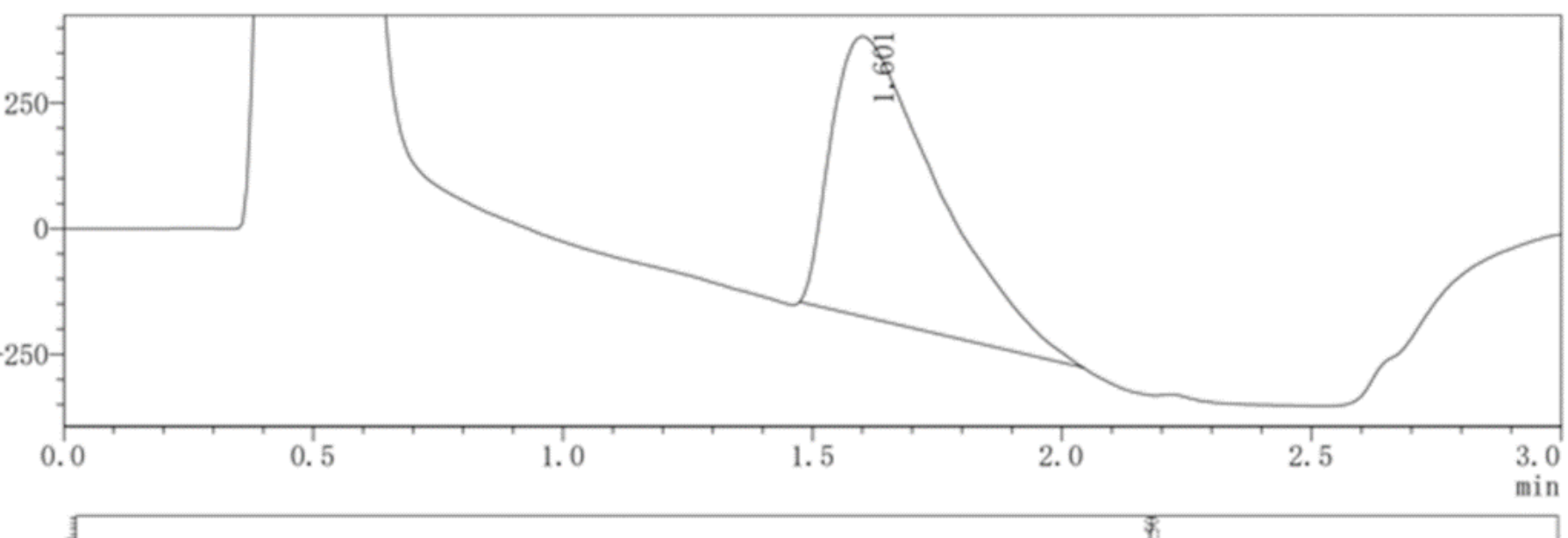
1.601
250
0
-250
0.0 0.5 1.0 1.5 2.0 2.5 3.0
min

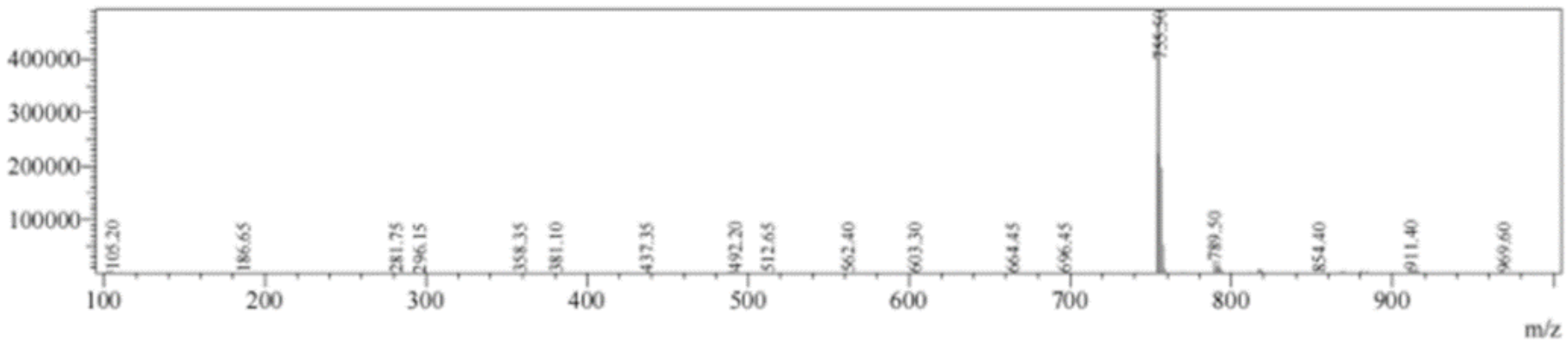
755.50
400000
300000
200000
100000
100 200 300 400 500 600 700 800 900
m/z

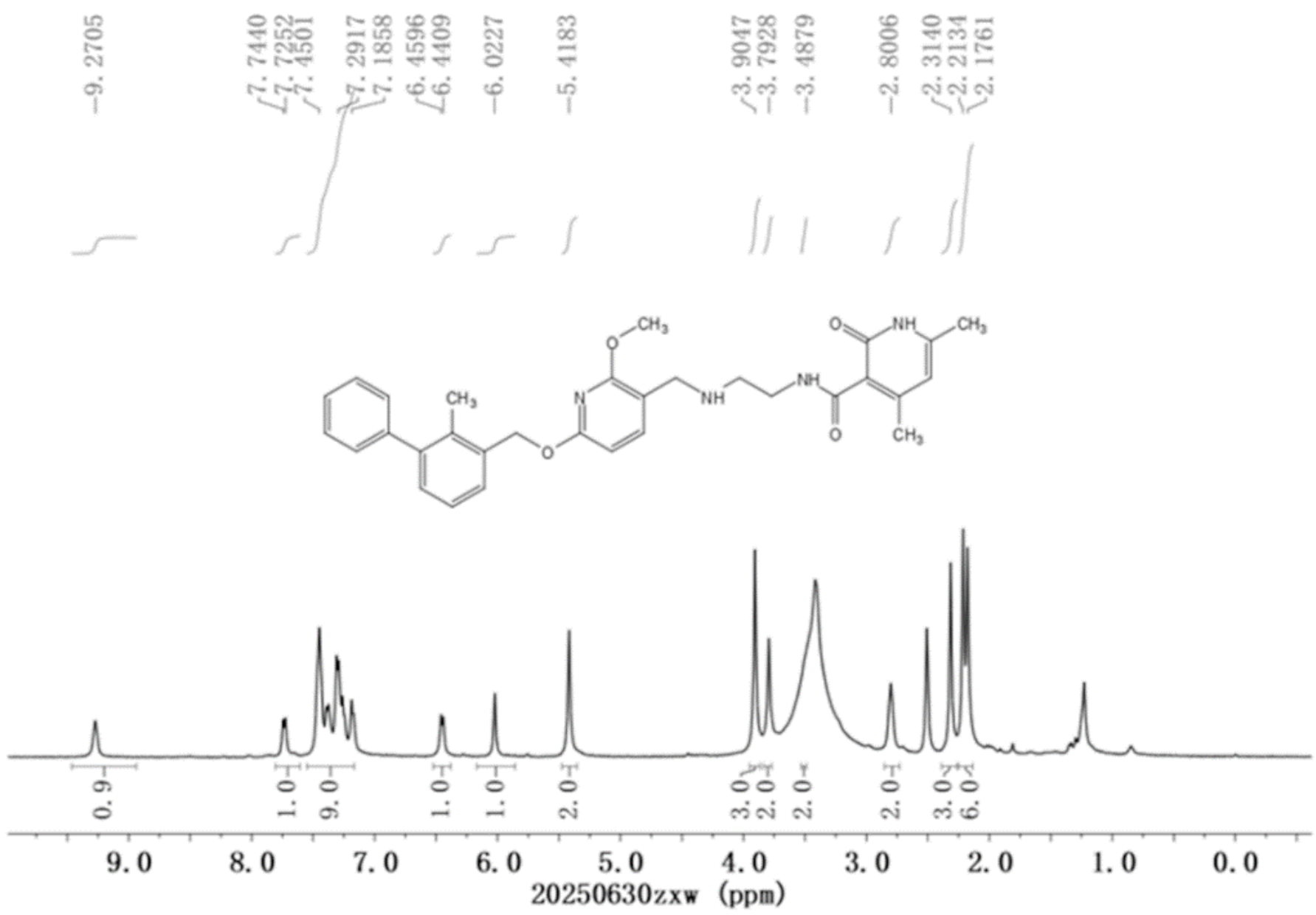
9.2705
7.7440
7.7252
7.4501
7.2917
7.1858
6.4596
6.4409
6.0227
5.4183
3.9047
3.7928
3.4879
2.8006
2.3140
2.2134
2.1761
CH3
N
O
NH
0.9
1.0
9.0
1.0
1.0
2.0
3.0
2.0
2.0
2.0
3.0
6.0
9.0 8.0 7.0 6.0 5.0 4.0 3.0 2.0 1.0 0.0
20250630zxw (ppm)

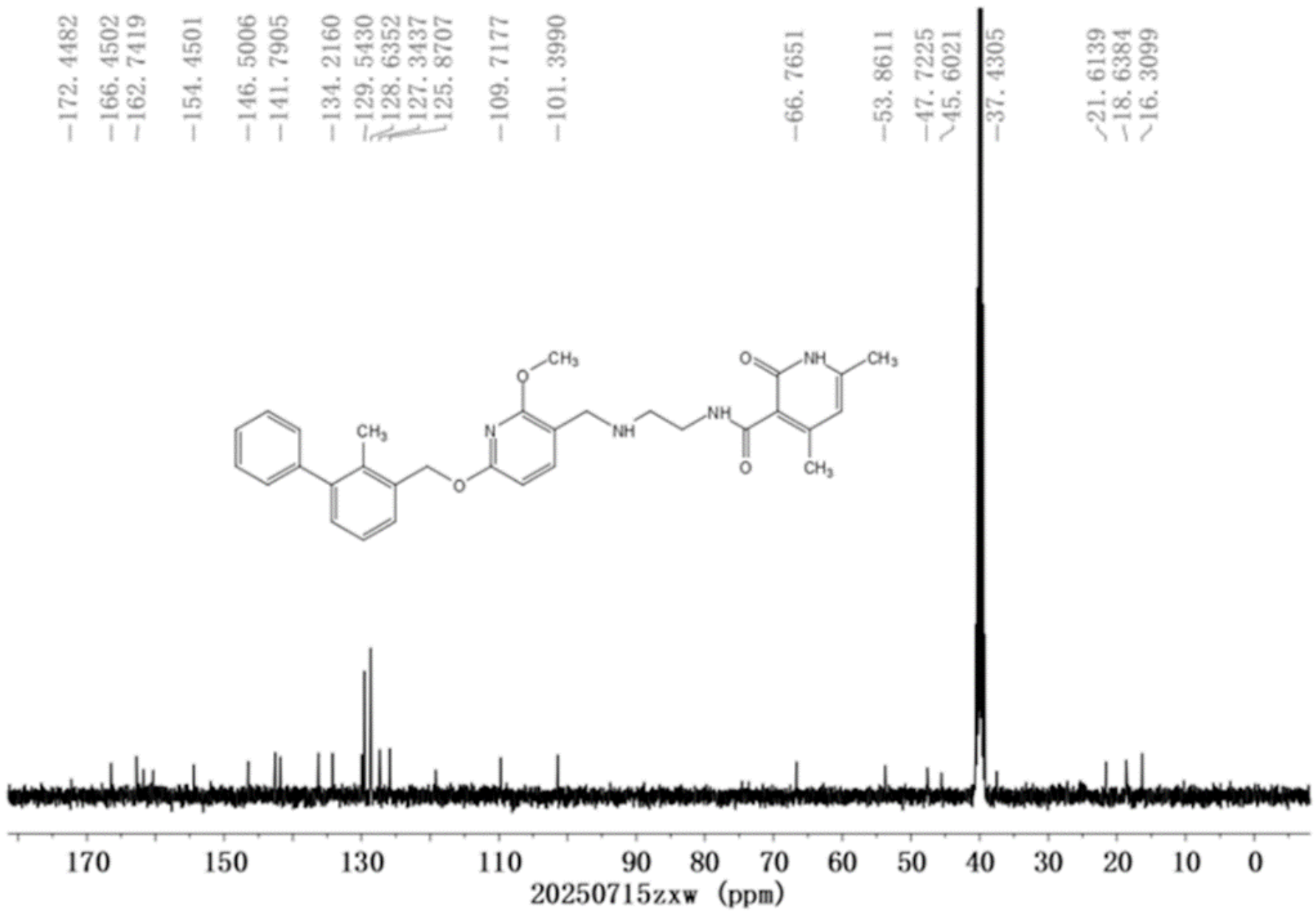
172.4482
166.4502
162.7419
154.4501
146.5006
141.7905
134.2160
129.5430
128.6352
127.3437
125.8707
109.7177
101.3990
66.7651
53.8611
47.7225
45.6021
37.4305
21.6139
18.6384
16.3099
170 150 130 110 90 80 70 60 50 40 30 20 10 0
20250715zxw (ppm)

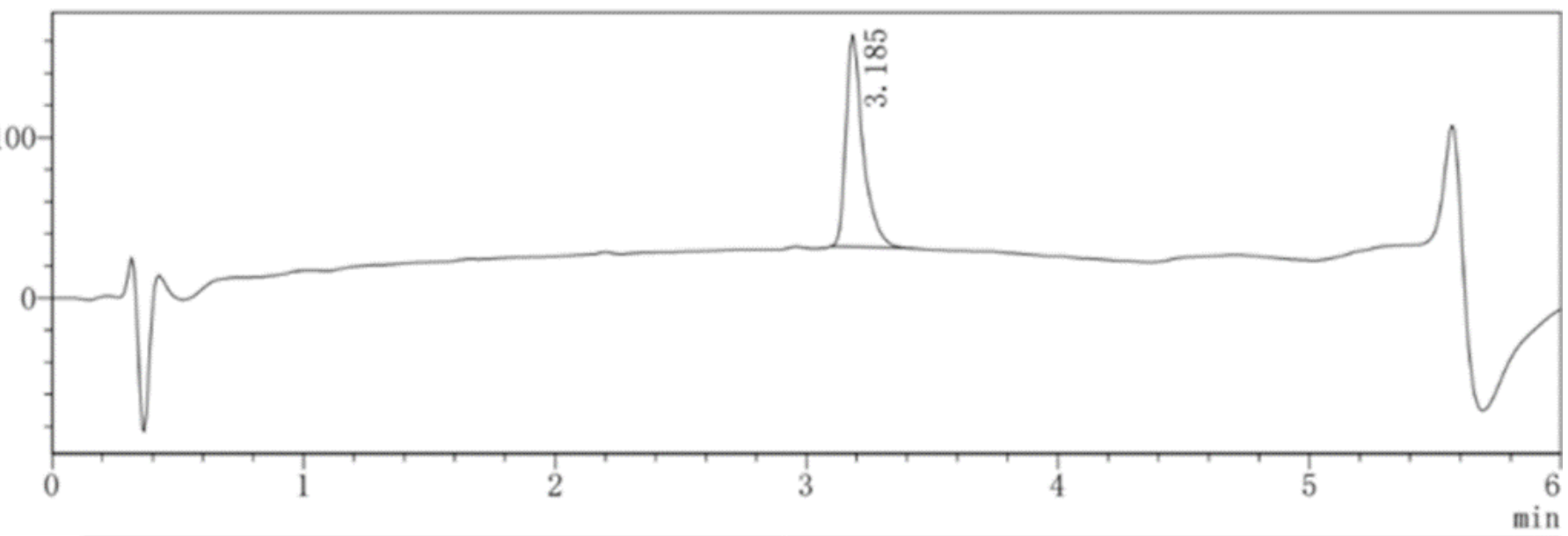
3.185
100
0
0 1 2 3 4 5 6
min

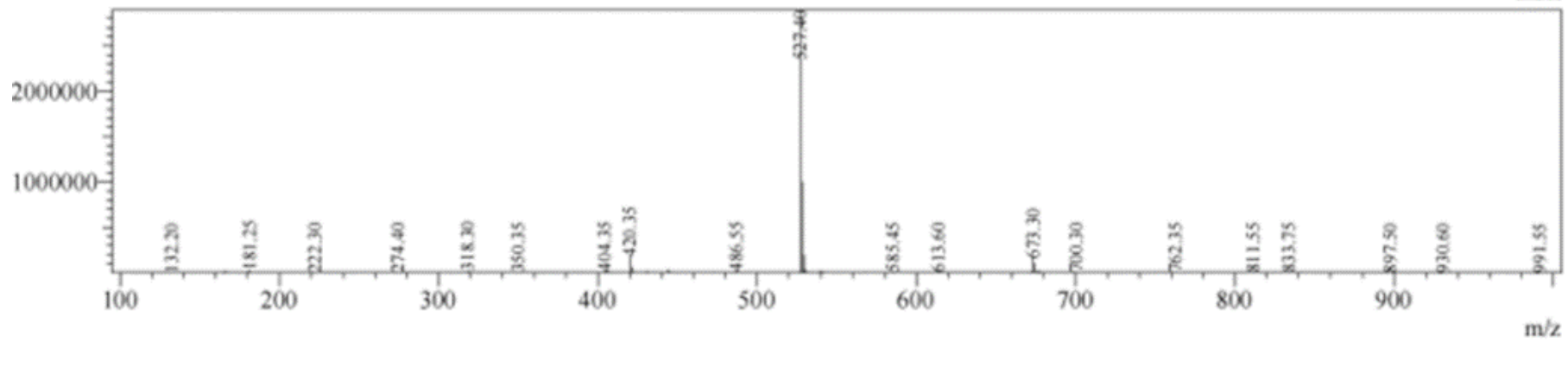
527.40
2000000
1000000
132.20
181.25
222.30
274.40
318.30
350.35
404.35
420.35
486.55
585.45
613.60
673.30
700.30
762.35
811.55
833.75
897.50
930.60
991.55
100 200 300 400 500 600 700 800 900
m/z

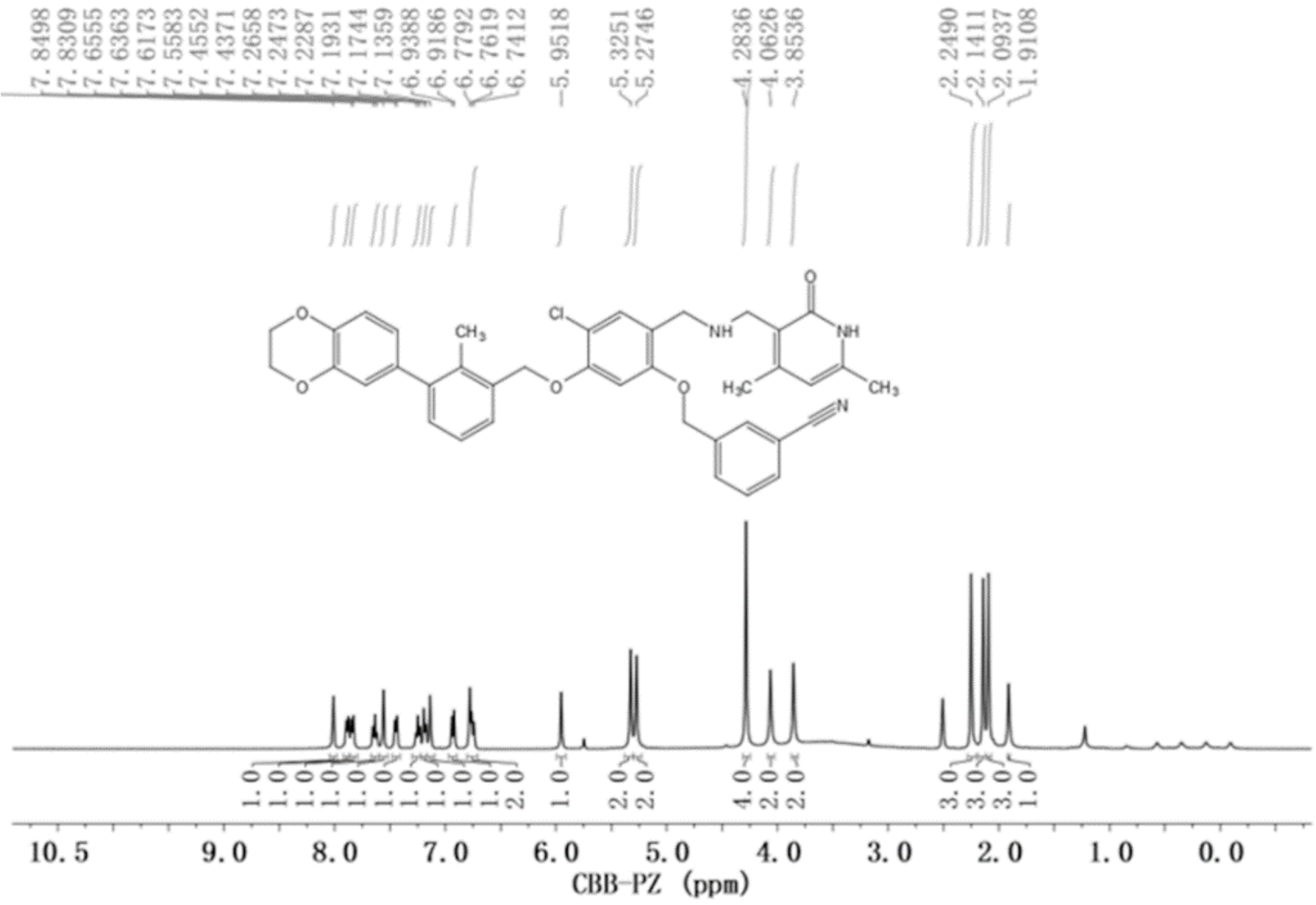
7.8498
7.8309
7.6555
7.6363
7.6173
7.5583
7.4552
7.4371
7.2658
7.2473
7.2287
7.1931
7.1744
7.1359
6.9388
6.9186
6.7792
6.7619
6.7412
5.9518
5.3251
5.2746
4.2836
4.0626
3.8536
2.2490
2.1411
2.0937
1.9108
Cl
CH3
NH
O
NH
H3C
CH3
N
O
O
O
O
1.0
1.0
1.0
1.0
1.0
1.0
1.0
1.0
1.0
1.0
2.0
1.0
2.0
2.0
4.0
2.0
2.0
3.0
3.0
3.0
1.0
10.5
9.0
8.0
7.0
6.0
5.0
4.0
3.0
2.0
1.0
0.0
CBB-PZ (ppm)

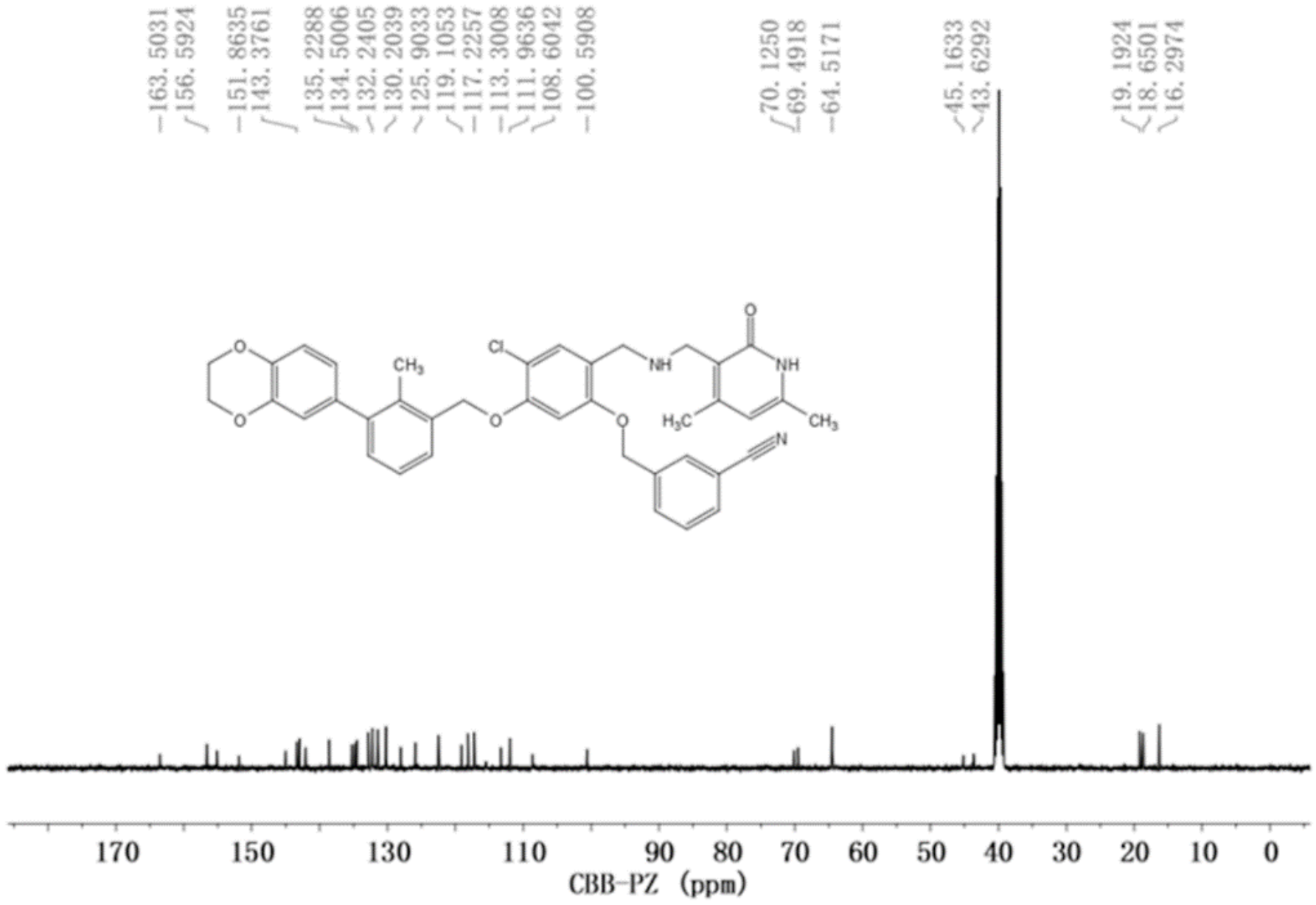
163.5031
156.5924
151.8635
143.3761
135.2288
134.5006
132.2405
130.2039
125.9033
119.1053
117.2257
113.3008
111.9636
108.6042
100.5908
70.1250
69.4918
64.5171
45.1633
43.6292
19.1924
18.6501
16.2974
Cl
CH3
NH
O
NH
H3C
CH3
N
170
150
130
110
90
80
70
60
50
40
30
20
10
0
CBB-PZ (ppm)

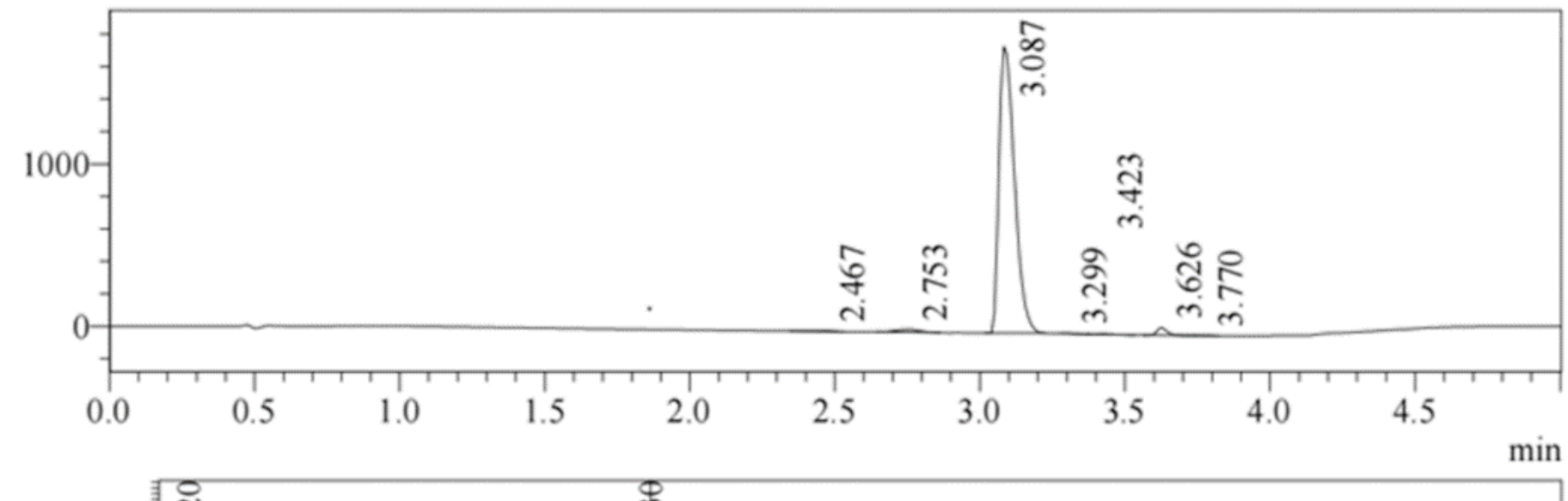
3.087
3.423
2.467
2.753
3.299
3.626
3.770
1000
0
0.0
0.5
1.0
1.5
2.0
2.5
3.0
3.5
4.0
4.5
min

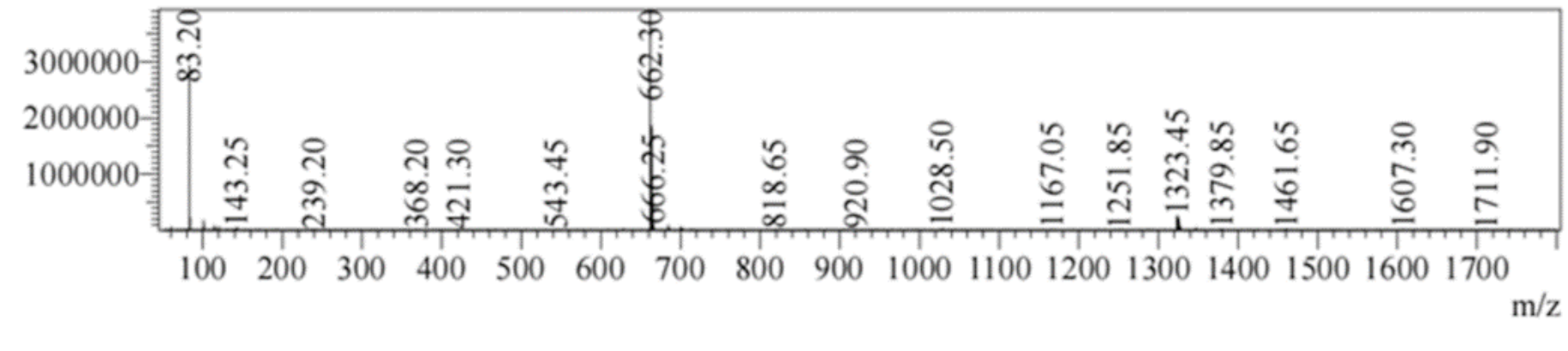
83.20
662.30
143.25
239.20
368.20
421.30
543.45
666.25
818.65
920.90
1028.50
1167.05
1251.85
1323.45
1379.85
1461.65
1607.30
1711.90
3000000
2000000
1000000
100
200
300
400
500
600
700
800
900
1000
1100
1200
1300
1400
1500
1600
1700
m/z

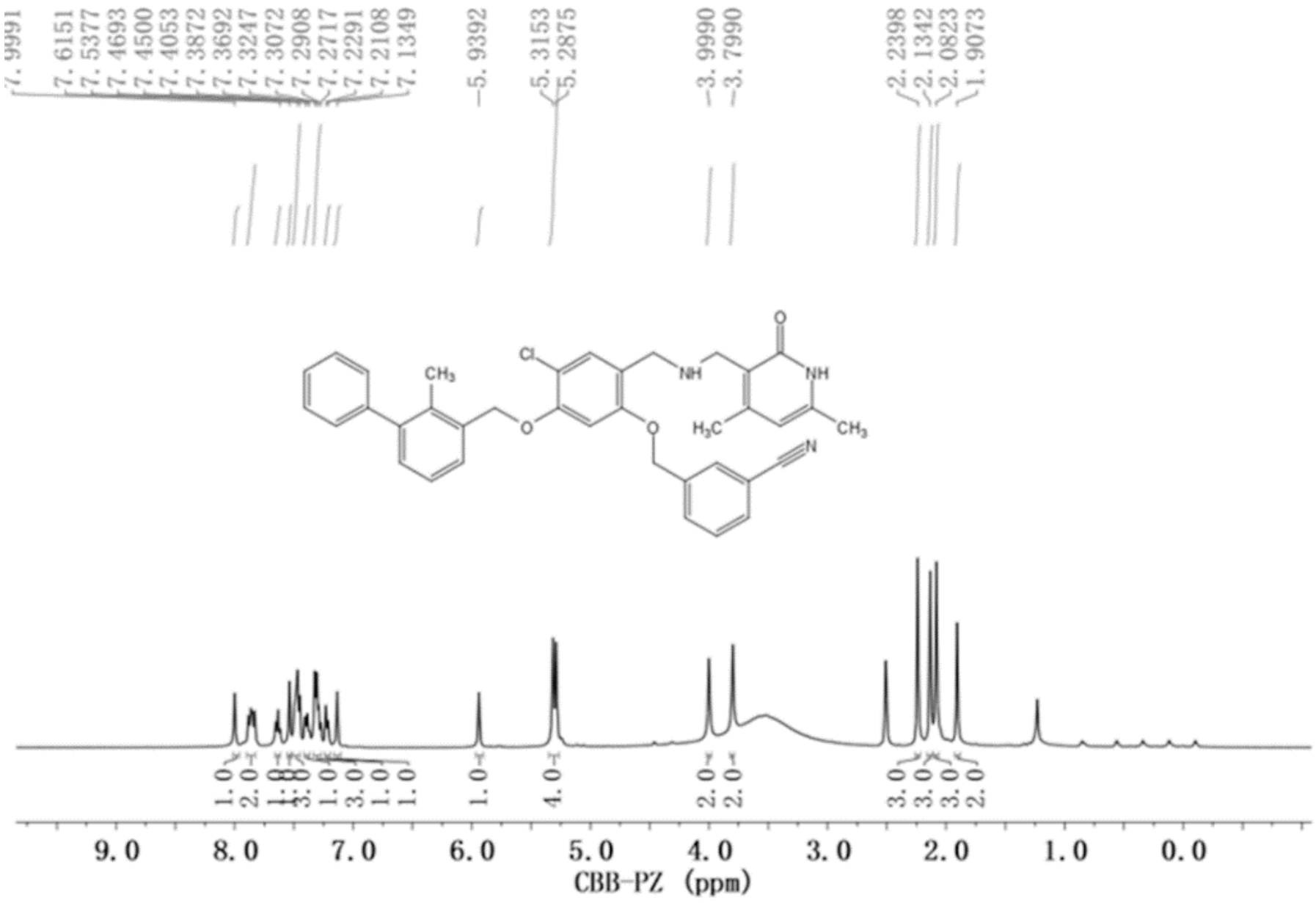
7.9991
7.6151
7.5377
7.4693
7.4500
7.4053
7.3872
7.3692
7.3247
7.3072
7.2908
7.2717
7.2291
7.2108
7.1349
5.9392
5.3153
5.2875
3.9990
3.7990
2.2398
2.1342
2.0823
1.9073
Cl
NH
O
NH
CH3
H3C
CH3
O
O
N
1.0
2.0
3.0
1.0
3.0
1.0
1.0
1.0
4.0
2.0
2.0
3.0
3.0
3.0
2.0
9.0
8.0
7.0
6.0
5.0
4.0
3.0
2.0
1.0
0.0
CBB-PZ (ppm)

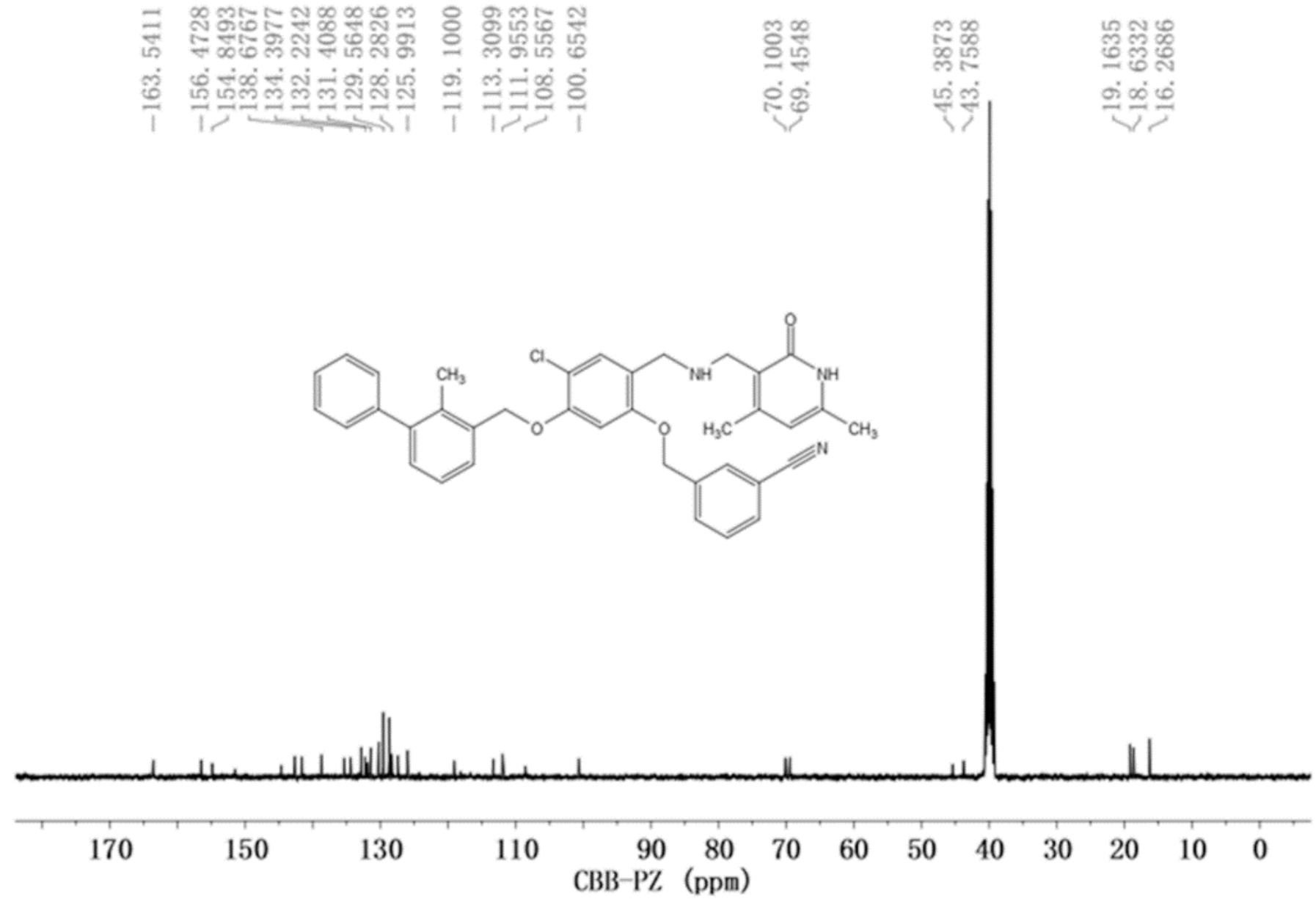
163.5411
156.4728
154.8493
138.6767
134.3977
132.2242
131.4088
129.5648
128.2826
125.9913
119.1000
113.3099
111.9553
108.5567
100.6542
70.1003
69.4548
45.3873
43.7588
19.1635
18.6332
16.2686
Cl
NH
O
NH
CH3
H3C
CH3
O
O
N
170
150
130
110
90
80
70
60
50
40
30
20
10
0
CBB-PZ (ppm)

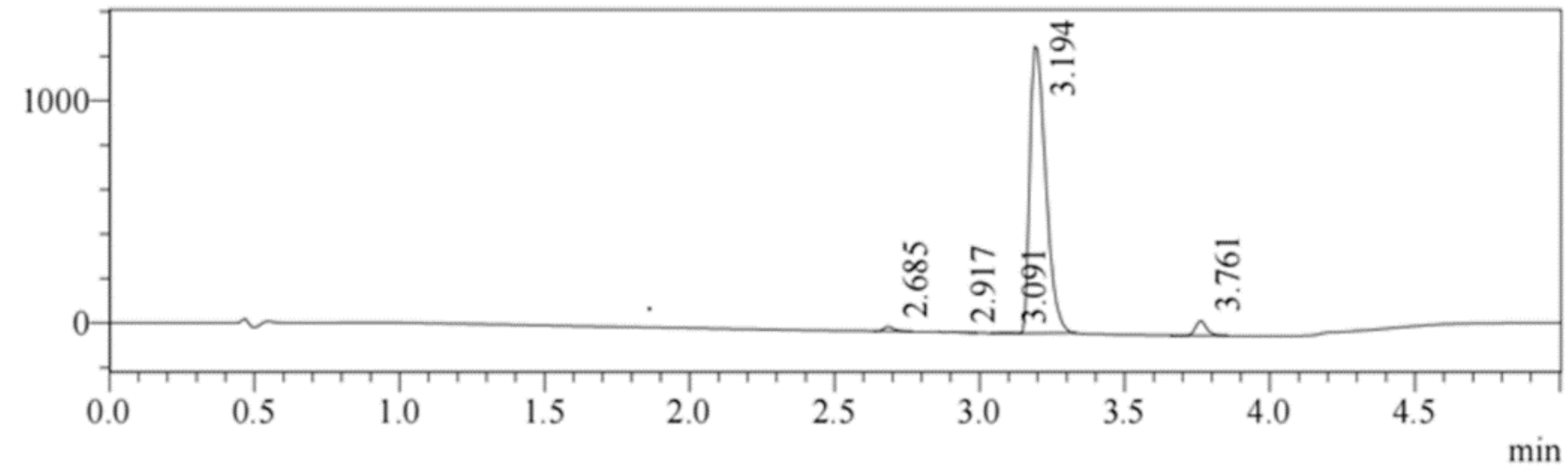
1000
0
3.194
2.685
2.917
3.091
3.761
0.0
0.5
1.0
1.5
2.0
2.5
3.0
3.5
4.0
4.5
min

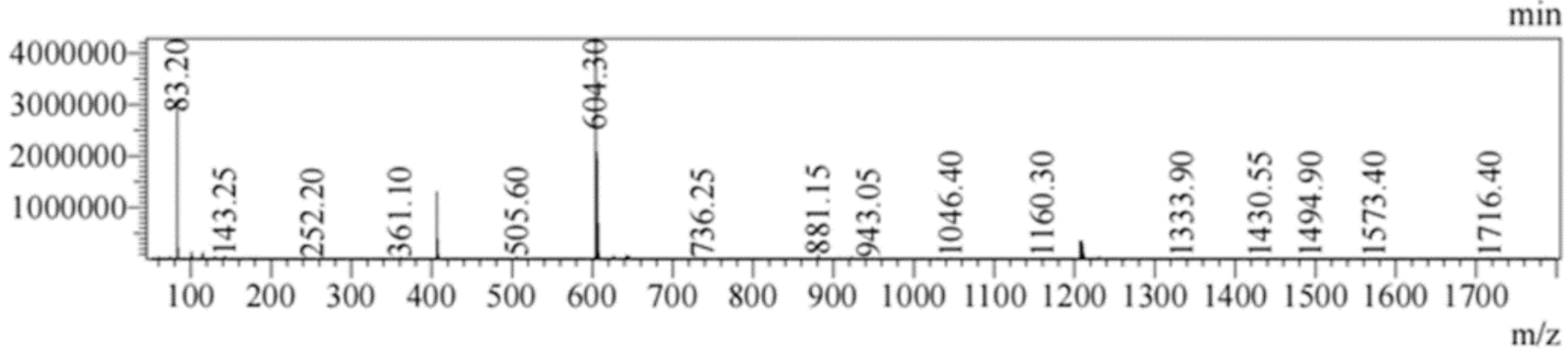
4000000
3000000
2000000
1000000
83.20
604.30
143.25
252.20
361.10
505.60
736.25
881.15
943.05
1046.40
1160.30
1333.90
1430.55
1494.90
1573.40
1716.40
100
200
300
400
500
600
700
800
900
1000
1100
1200
1300
1400
1500
1600
1700
m/z

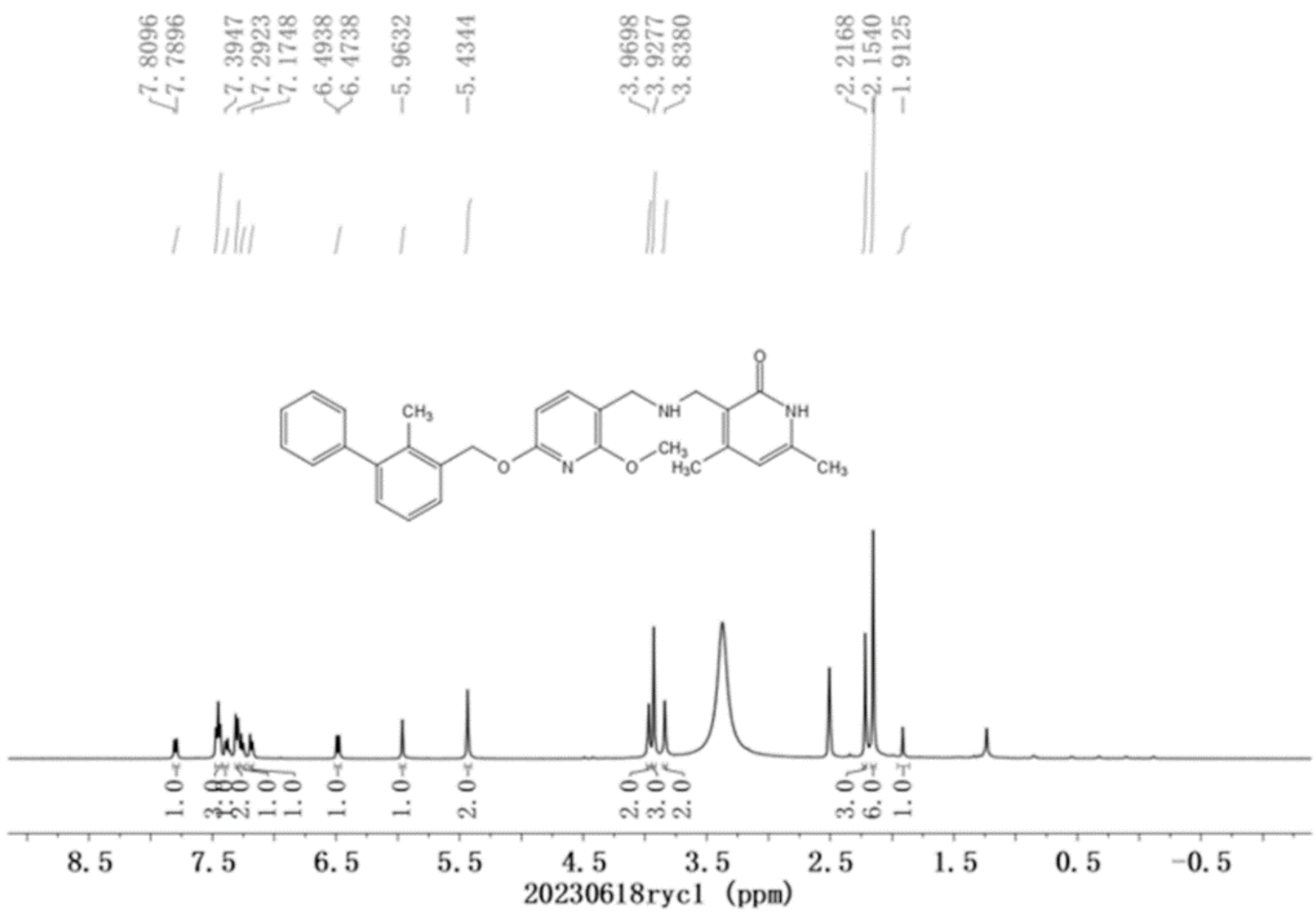
7.8096
7.7896
7.3947
7.2923
7.1748
6.4938
6.4738
5.9632
5.4344
3.9698
3.9277
3.8380
2.2168
2.1540
1.9125
CH3
NH
O
O
N
O
CH3
H3C
CH3
1.0
3.0
2.0
1.0
1.0
1.0
1.0
2.0
2.0
3.0
2.0
3.0
6.0
1.0
8.5
7.5
6.5
5.5
4.5
3.5
2.5
1.5
0.5
-0.5
20230618ryc1 (ppm)

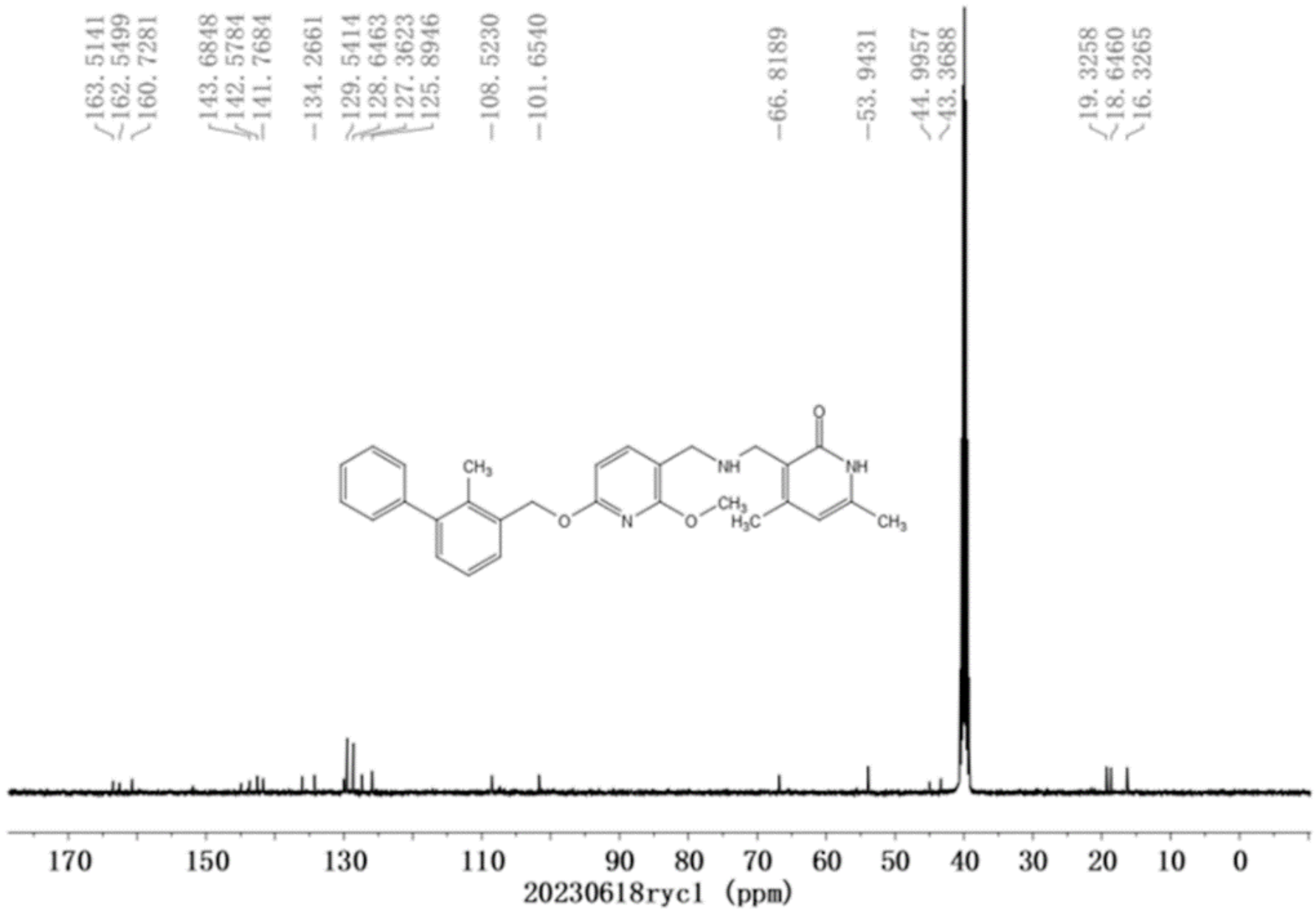
163.5141
162.5499
160.7281
143.6848
142.5784
141.7684
134.2661
129.5414
128.6463
127.3623
125.8946
108.5230
101.6540
66.8189
53.9431
44.9957
43.3688
19.3258
18.6460
16.3265
170
150
130
110
90
80
70
60
50
40
30
20
10
0
20230618ryc1 (ppm)

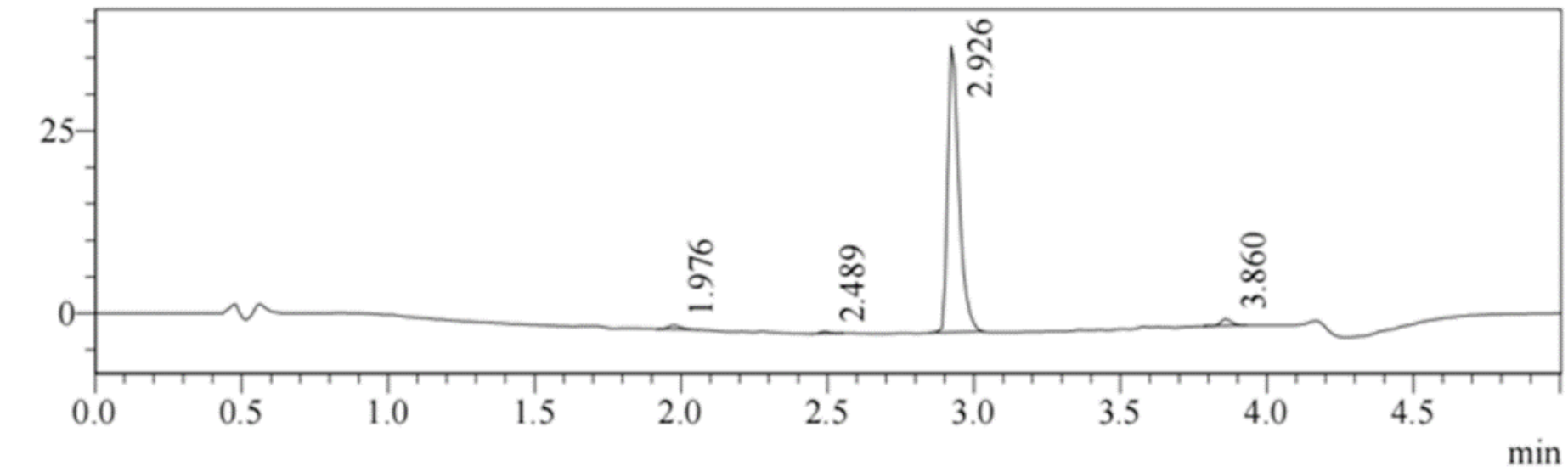
25
0
2.926
1.976
2.489
3.860
0.0
0.5
1.0
1.5
2.0
2.5
3.0
3.5
4.0
4.5
min

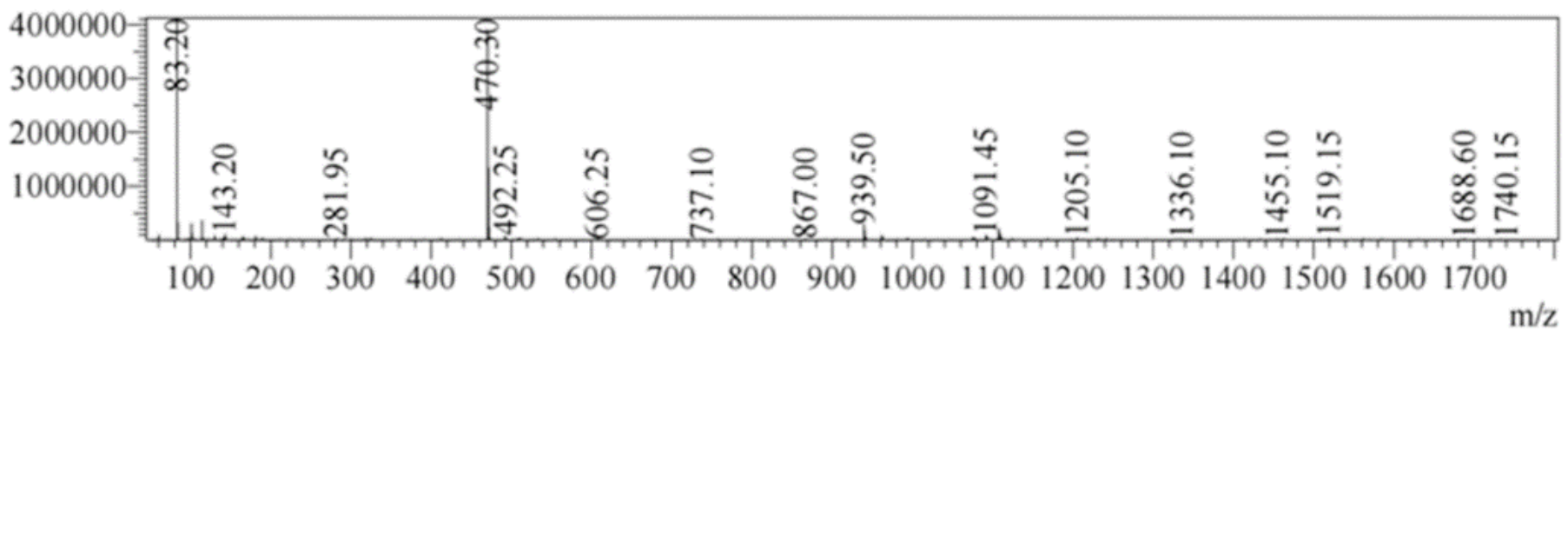
4000000
3000000
2000000
1000000
83.20
470.30
143.20
281.95
492.25
606.25
737.10
867.00
939.50
1091.45
1205.10
1336.10
1455.10
1519.15
1688.60
1740.15
100
200
300
400
500
600
700
800
900
1000
1100
1200
1300
1400
1500
1600
1700
m/z

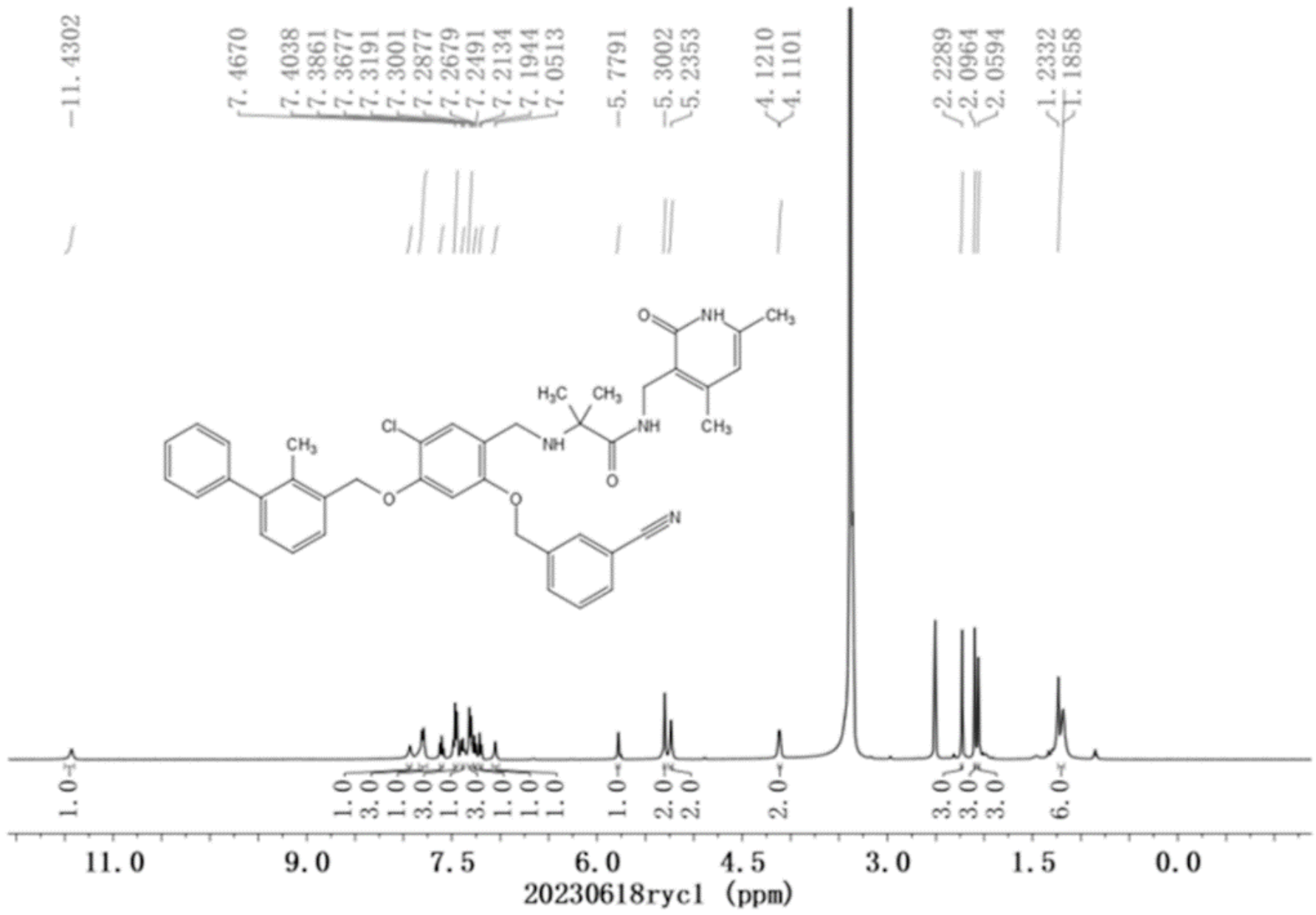
20230618ryc1 (ppm)

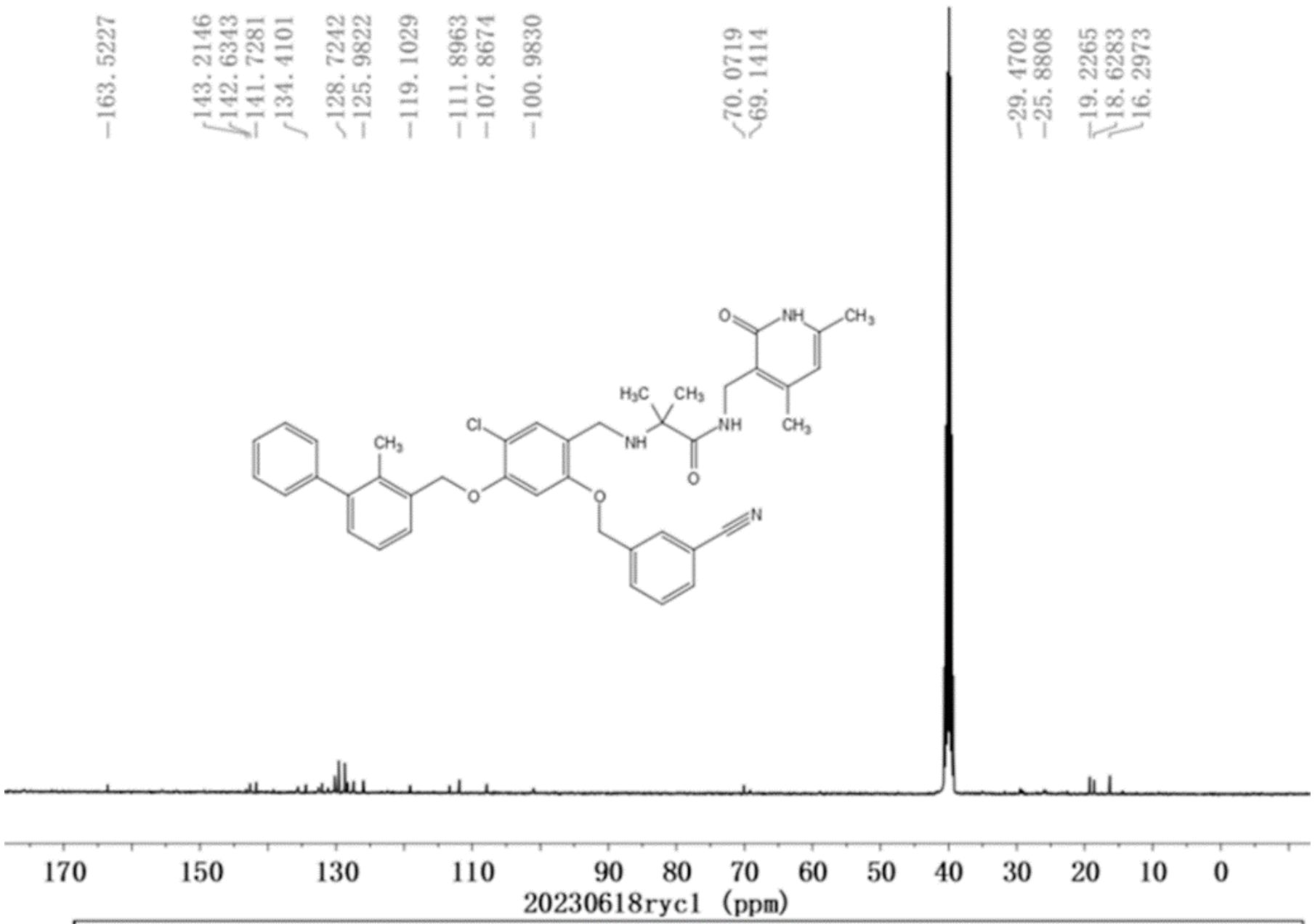
20230618ryc1 (ppm)

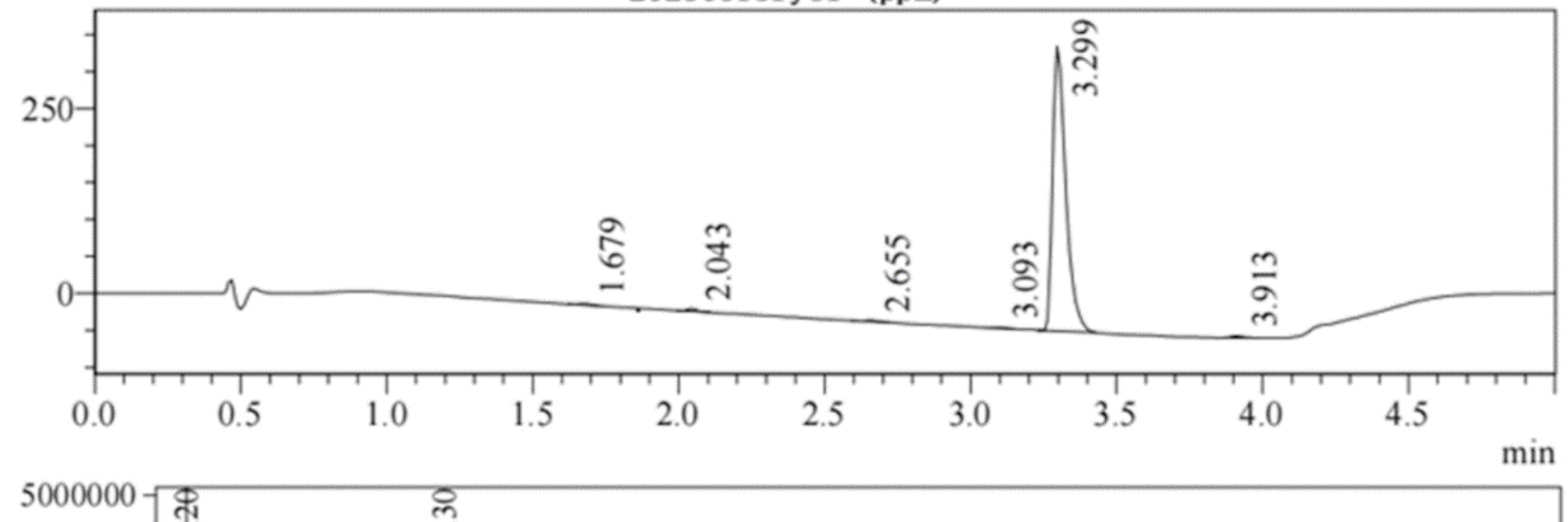

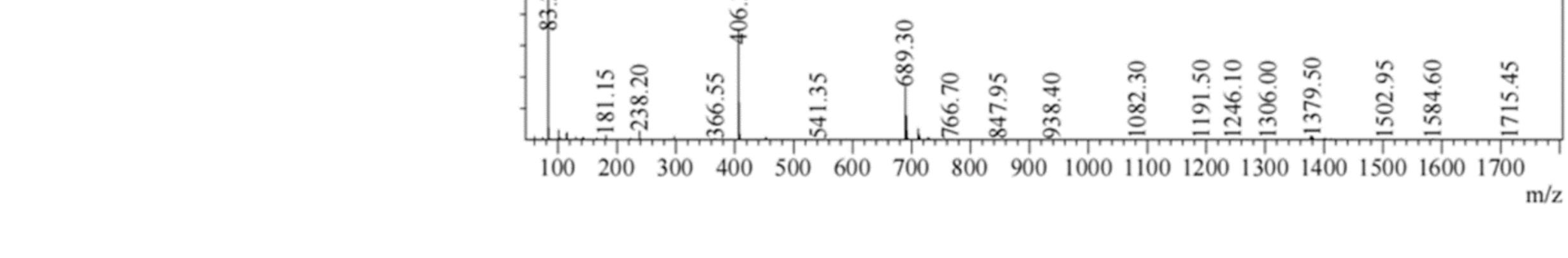

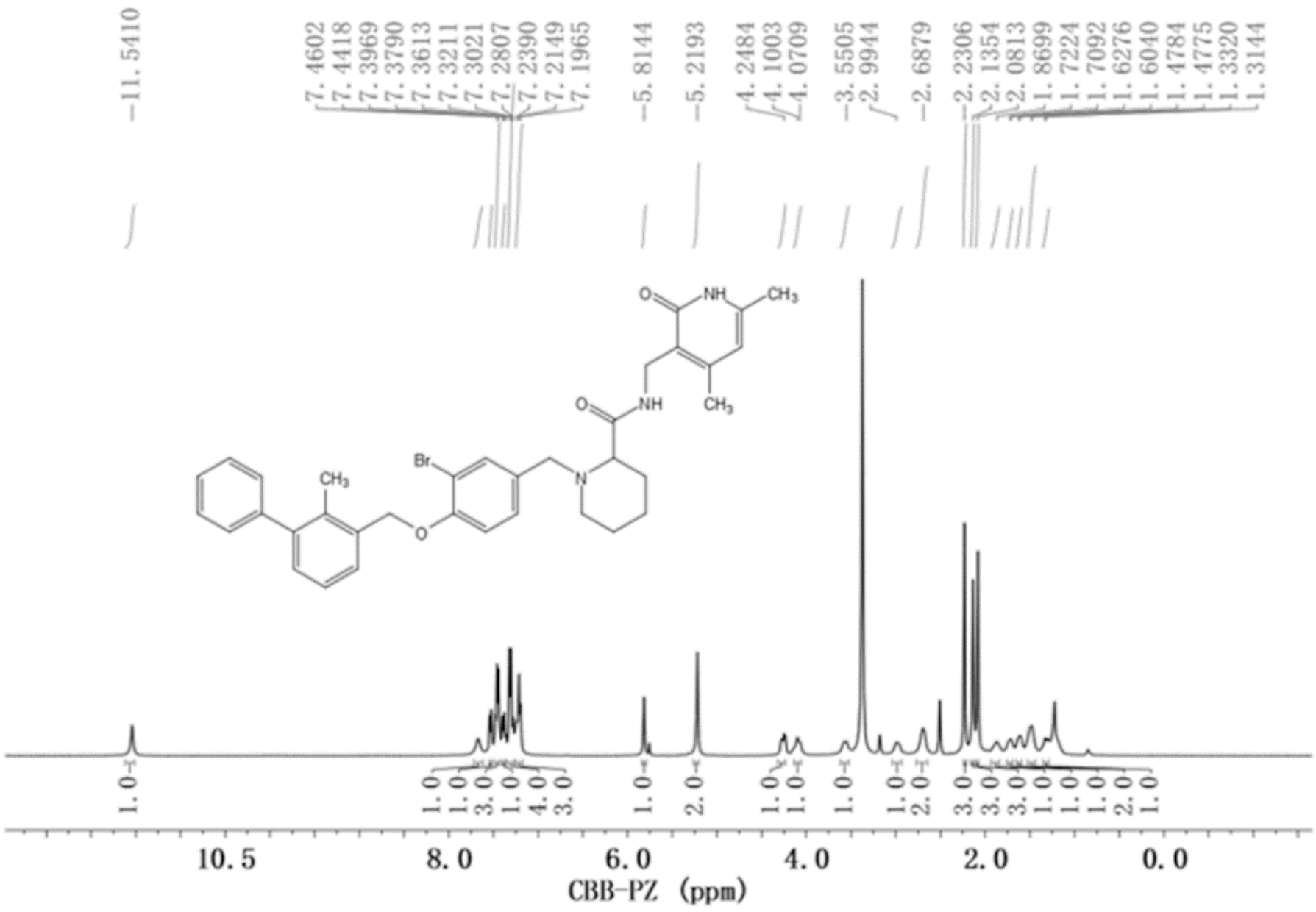
CBB-PZ (ppm)

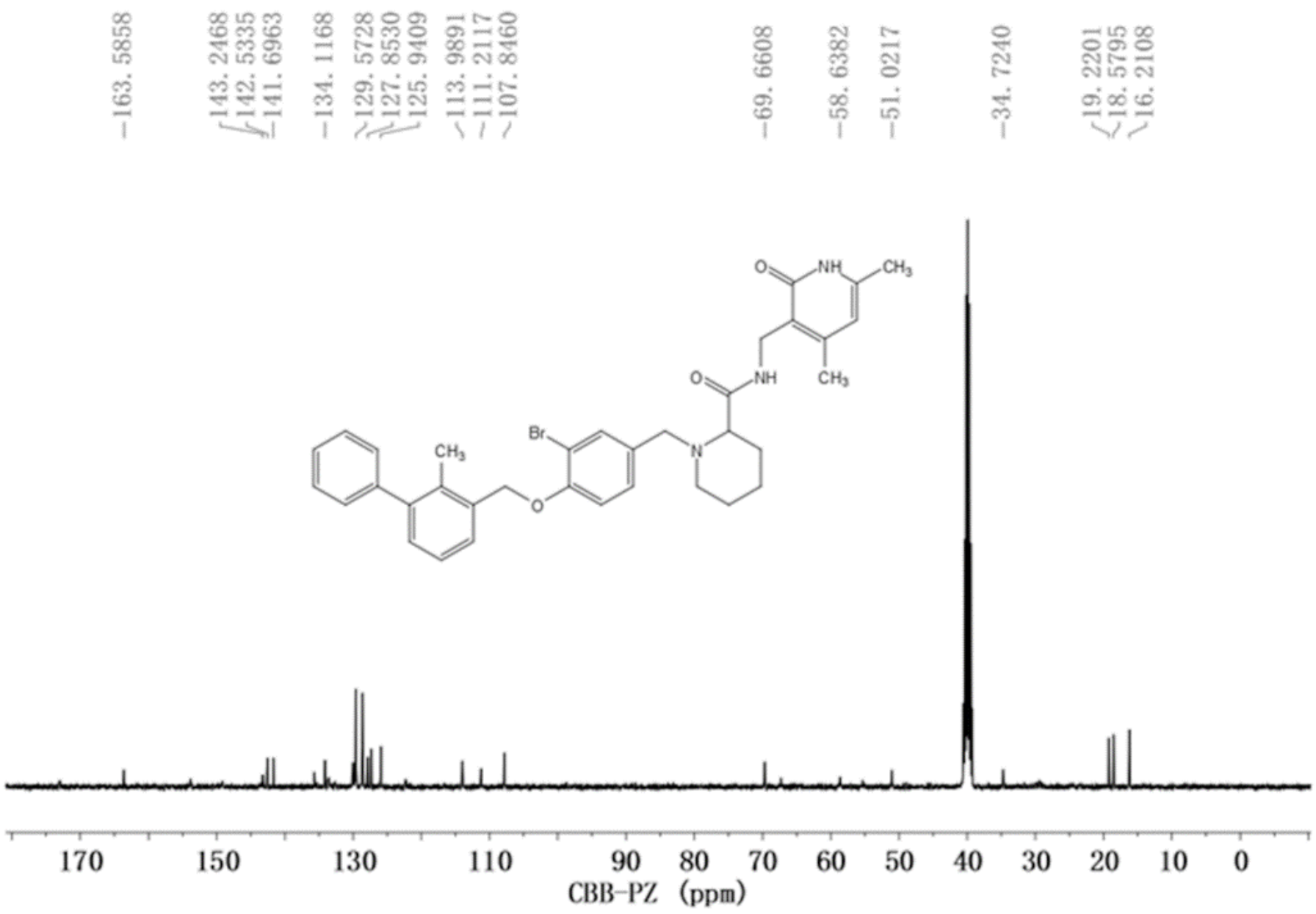
CBB-PZ (ppm)

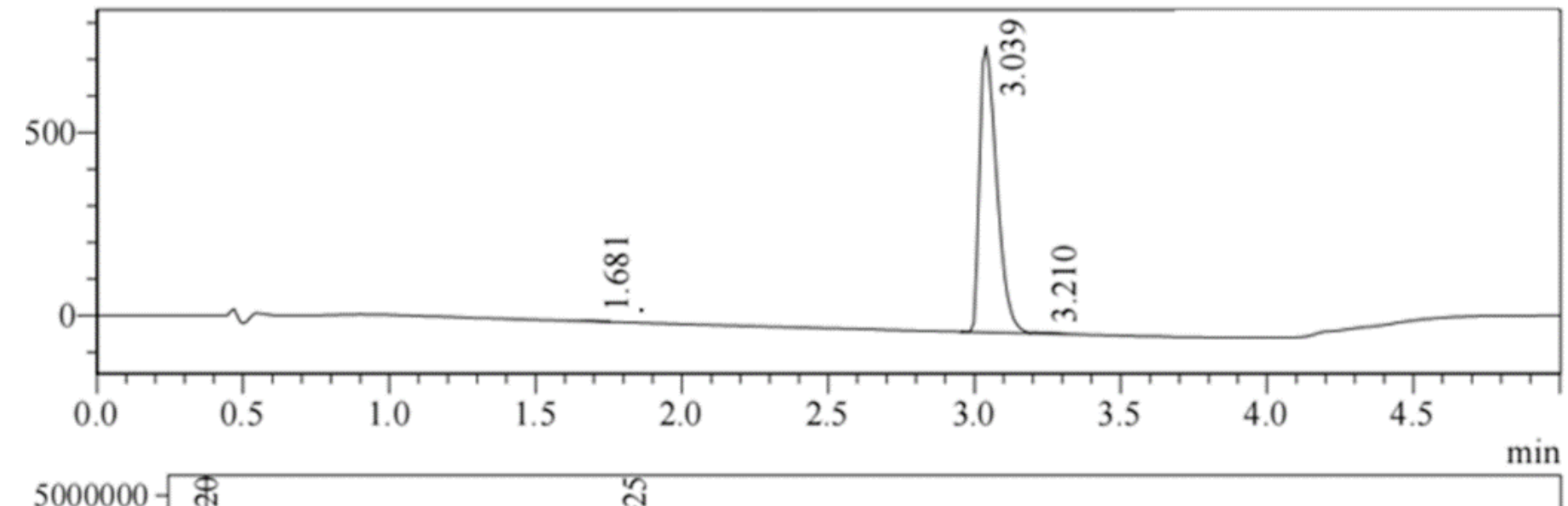
3.039
1.681
3.210
min

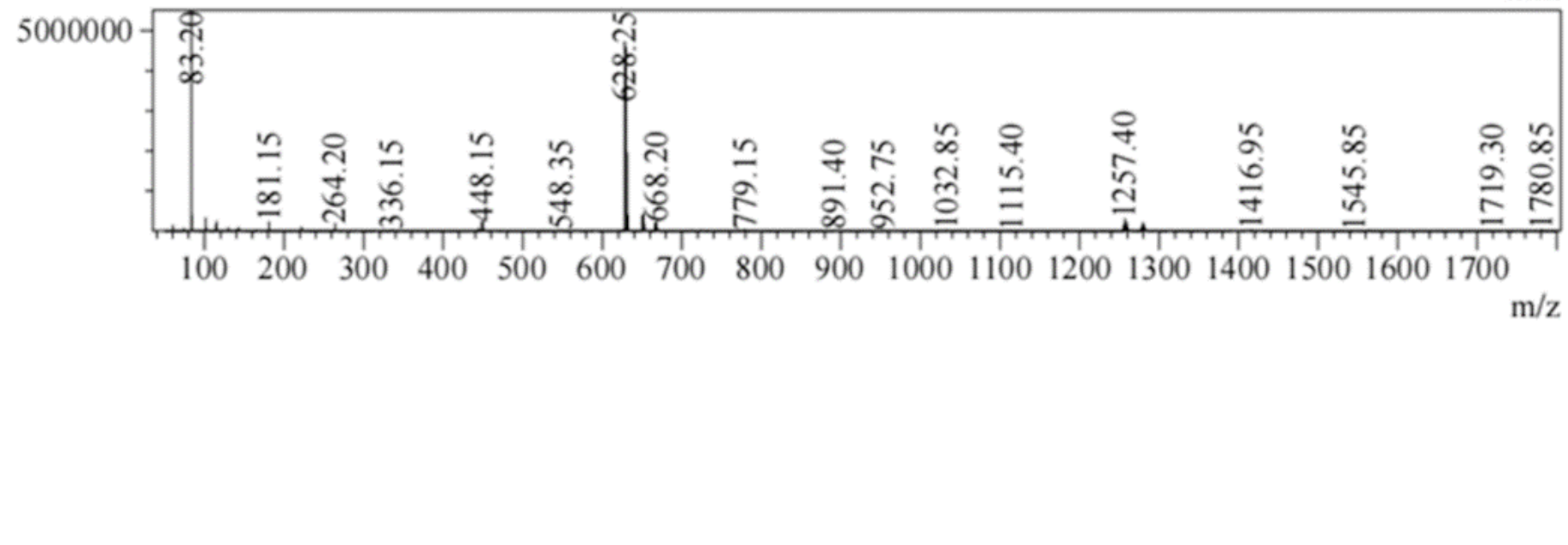
83.20
628.25
m/z
